# Excitatory Spinal Lhx9-Derived Interneurons Modulate Locomotor Frequency in Mice

**DOI:** 10.1523/JNEUROSCI.1607-23.2024

**Published:** 2024-03-04

**Authors:** Maëlle Bertho, Vanessa Caldeira, Li-Ju Hsu, Peter Löw, Lotta Borgius, Ole Kiehn

**Affiliations:** ^1^Department of Neuroscience, Karolinska Institutet, 17177 Stockholm, Sweden; ^2^Department of Neuroscience, University of Copenhagen, 2200 Copenhagen, Denmark

**Keywords:** central pattern generator, gene expression, motor control, rhythm, spinal neurons

## Abstract

Locomotion allows us to move and interact with our surroundings. Spinal networks that control locomotion produce rhythm and left–right and flexor–extensor coordination. Several glutamatergic populations, Shox2 non-V2a, Hb9-derived interneurons, and, recently, spinocerebellar neurons have been proposed to be involved in the mouse rhythm generating networks. These cells make up only a smaller fraction of the excitatory cells in the ventral spinal cord. Here, we set out to identify additional populations of excitatory spinal neurons that may be involved in rhythm generation or other functions in the locomotor network. We use RNA sequencing from glutamatergic, non-glutamatergic, and Shox2 cells in the neonatal mice from both sexes followed by differential gene expression analyses. These analyses identified transcription factors that are highly expressed by glutamatergic spinal neurons and differentially expressed between Shox2 neurons and glutamatergic neurons. From this latter category, we identified the Lhx9-derived neurons as having a restricted spinal expression pattern with no Shox2 neuron overlap. They are purely glutamatergic and ipsilaterally projecting. Ablation of the glutamatergic transmission or acute inactivation of the neuronal activity of Lhx9-derived neurons leads to a decrease in the frequency of locomotor-like activity without change in coordination pattern. Optogenetic activation of Lhx9-derived neurons promotes locomotor-like activity and modulates the frequency of the locomotor activity. Calcium activities of Lhx9-derived neurons show strong left–right out-of-phase rhythmicity during locomotor-like activity. Our study identifies a distinct population of spinal excitatory neurons that regulates the frequency of locomotor output with a suggested role in rhythm-generation in the mouse alongside other spinal populations.

## Significance Statement

Ipsilaterally projecting excitatory interneurons play a crucial role in the generation of locomotor rhythms in the vertebrate spinal cord. While the Shox2, Hb9, and spinocerebellar tract neurons are known components involved in this behavior they represent only a fraction of the entire excitatory population in the spinal cord. In this study, we identify additional excitatory spinal populations that could be essential contributors to the locomotor circuitry and shed light on a new population of ipsilaterally projecting excitatory neurons expressing the transcription factor Lhx9. Our findings show that this newly identified population has the capacity to modulate the locomotor frequency, suggesting a potential involvement in the rhythm generating network along other spinal neuronal populations.

## Introduction

Locomotor movements are complex motor actions that are essential for animals and humans to navigate through their environments. In vertebrates, the precise timing and pattern of locomotor activity involve spinal neuronal networks (Goulding, 2009; Kiehn, 2016; Grillner and El Manira, 2020). These circuits contain several interconnected populations of excitatory and inhibitory interneurons which produce the pattern and rhythmicity underlying locomotor movements. Defining the organizing principles of spinal motor circuitry depends on determining the diversity of the discrete interneuron subtypes that constitute it. The classification of spinal neurons into 11 cardinal groups — ventral, V0–V3, and dorsal, dI1–dI6 domains (Jessell, 2000; Goulding and Pfaff, 2005; Goulding, 2009) — and motor neurons has been instrumental in this endeavor. Targeted inactivation and/or activation of specific cardinal neuronal populations allowed a better understanding of the neuronal circuit organization underlying left–right (Lanuza et al., 2004; Quinlan and Kiehn, 2007; Crone et al., 2008, 2009; Zhang et al., 2008; Talpalar et al., 2013; Bellardita and Kiehn, 2015; Haque et al., 2018; Schnerwitzki et al., 2018) and flexor–extensor coordination (Zhang et al., 2008, 2015; Talpalar et al., 2011; Britz et al., 2015; Danner et al., 2019) as well as the rhythm generation of locomotor activity (Zhang et al., 2008; Dougherty et al., 2013; Ampatzis et al., 2014; Ljunggren et al., 2014; Caldeira et al., 2017; Song et al., 2020; Chalif et al., 2022). In mammals, these networks controlling hindlimb locomotion are found throughout the lumbar spinal cord with a rhythmogenic rostro-caudal gradient (Kjaerulff and Kiehn, 1996; Cowley and Schmidt, 1997; Kremer and Lev-Toy, 1997).

The principal feature of rhythm generating networks is to set and/or modulate the frequency of locomotion. In all vertebrates, ipsilaterally projecting excitatory interneurons are critical components of these networks (Dale and Roberts, 1985; Buchanan and Grillner, 1987; Kjaerulff and Kiehn, 1996; Grillner, 2003; Kiehn, 2006; Hägglund et al., 2010, 2013; Grillner and El Manira, 2020). In the zebrafish, last order V2a Chx10 neurons constitute the core of the rhythm generating circuits (Ampatzis et al., 2014; Ljunggren et al., 2014; Song et al., 2020). In the mouse, the V2a population (Chx10^+^) overlap with the excitatory Shox2 population resulting in the delineation of three distinctive groups within the ventral spinal cord: Shox2 non-V2a (also known as Shox2^+^-Chx10^−^), “pure” Chx10 (Shox2^−^-Chx10^+^), and Shox2^+^ V2a neurons (Shox2^+^-Chx10^+^) (Dougherty et al., 2013). The removal of the Chx10 or Shox2^+^ V2a population has distinct impacts on locomotor coordination: Chx10 elimination affects left–right alternation, while Shox2^+^ V2a neuron removal influences motor neuron burst amplitude modulation. Importantly, neither alteration has any discernible impacts on the frequency of locomotion (Dougherty et al., 2013). In contrast, the Shox2 non-V2a interneurons as well as the broader class of Hb9Cre-derived excitatory interneurons — which are non-overlapping with Shox2 non-V2a interneurons (Caldeira et al., 2017) — have been assigned a role in the rhythm generating in the mouse. However, in contrast to silencing all glutamatergic neurons (Hägglund et al., 2013) or the V2a neurons in zebrafish that completely blocks the rhythm, silencing Shox2 non-V2a or the broader class of Hb9Cre-derived excitatory neurons only partially abolished the rhythm which suggested that additional excitatory populations might contribute to the rhythm generation circuits in the mouse. Recently, the glutamatergic spinocerebellar tract neurons have also been shown to be implicated in rhythm generation circuits in the neonatal mouse spinal cord (Chalif et al., 2022). It is unknown whether the spinocerebellar tract neurons overlap with the two other groups of rhythm generating neurons. Moreover, these cell populations make up only a smaller fraction of all the excitatory cells in the ventral spinal cord. Here, we set out to identify additional populations of excitatory spinal neurons that may be involved in rhythm generation or in other aspects of locomotor performance.

For this, we first used RNA sequencing of glutamatergic, non-glutamatergic, and Shox2 cells in the ventral spinal cord where the locomotor network is localized followed by differential expression analysis of these populations. The differential expression analysis identified several transcription factors that were enriched in the different populations. In particular, we found that the transcription factor LIM homeobox 9 (Lhx9) was in the top of differentially expressed transcription factors enriched in spinal glutamatergic non-Shox2 neurons and that these cells form a distinct ipsilaterally projecting excitatory population in the lumbar spinal cord. The functional analysis of Lhx9-derived neurons revealed a role in modulating the frequency of the locomotor rhythm suggesting that these cells may additionally contribute to rhythm generation directly or indirectly in the mouse spinal cord.

## Materials and Methods

### Animals

All animal experiments and procedures followed the European guidelines for the care and use of laboratory animal and were approved by Stockholm Norra Djurförsöksetisk nämnd (permit number; N42/16, N139/13, N284/15 and 3644-2019), and Dyreforsøgstilsynet (permit: 2017-15-0201-01246 and 2022-15-0201-01187).

#### Mice line

The following transgenic lines of both sexes were used: *Vglut2-GFP* mice generated in our lab by Dr. Borgius using BAC recombination with an analogous strategy to the one described previously (Borgius et al., 2010); *Shox2Cre* mice kindly provided by Drs. Jessell and Zagoraiou (Dougherty et al., 2013); *Ai39* (B6;129S-Gt(ROSA)26Sor^tm39(CAG-hop/EYFP)Hze^/J, Jackson Laboratory, Strain #014539; here named *Rosa26-YFP*); *Vglut2^Flox/Flox^* mice kindly provided by Drs. Hnasko (Hnasko et al. (2010), previously described in Talpalar et al. (2011) and Caldeira et al. (2017)); *RC::FPD*i (B6;129S6-Gt(ROSA)26Sor^tm9(CAG-mCherry,-CHRM4*)Dym^/J, Jackson Laboratory, Strain #029040); *Hoxb8FlpO* mice generated in our lab by Drs. Borgius and Peter Löw (Allodi et al., 2021); *Ai32* (B6;129S-Gt(ROSA)26Sor^tm32(CAG-COP4*H134R/EYFP)Hze^/J, Jackson laboratory, Strain #012569, here named *ChR2-EYFP*); *Ai14* (B6.Cg-Gt(ROSA)26Sor^tm14(CAG-tdTomato)Hze^/J, Jackson Laboratory, Strain #007914, here named *Rosa26-tdTomato*); *Lhx9Cre^ERT2^* kindly provided by Dr. Lin., Rochester University (previously described in Balasubramanian et al. (2014)); *Ai148* (B6.Cg-Igs7^tm148.1(tetO-GCaMP6f,CAG-tTA2)Hze/J^, Jackson Laboratory, Strain #030328; here named *GCamp6f*). All breeding animals were kept under a 12 h light/dark cycle with access to food and water *ad libitum* (housing temperature: 23–24°C and humidity: 45–65%).

Briefly, for conditional deletion of Vglut2, first *Vglut2^Flox/Flox^* mice in which the loxP sites flanked the exon 2 of the Slc17a6 gene (which encodes for Vglut2) on both alleles were generated (Talpalar et al., 2011). In parallel, crosses of *Lhx9Cre^ERT2^ mice* with *Vglut2^Flox/+^* mice were made to obtain the double transgenic line, *Lhx9Cre^ERT2^;Vglut2^Flox/+^*. Finally, these double transgenic lines were crossed with the homozygous *Vglut2^Flox/Flox^* mice to generate *Lhx9Cre^ERT2^;Vglut2^Flox/Flox^* with conditional removal of Vglut2 on both alleles.

### Timed expression of Lhx9-derived cells

To obtain timed expression of Lhx9-derived cells we used a *Lhx9Cre^ERT2^* mice line where Cre expression is tamoxifen-dependent. Breeding pairs were placed together overnight and embryonic day 0.5 (E0.5) was defined as the morning of the separation. At specific embryonic stages (E10.5, E11.5, E13.5 or E14.5), pregnant females were given an oral gavage of tamoxifen (Stock solution: 5 mg/mL in corn oil; dose: 0.1mL/20g body weight) (#T5648, Sigma-Aldrich). Neonatal mice were delivered via cesarean section (c-section) on embryonic day E18.5. To keep the neonatal mice alive for several days during the electrophysiology experiments, surrogate mice were used. For tamoxifen induction at postnatal days 0 and 1 (P0–P1), tamoxifen was given by activating the suction reflex of the neonatal mice and consequently, were kept alive with surrogates for tissue extraction at P5.

### Cell dissociation and fluorescence activated cell sorting (FACS)

**Figure 1.**
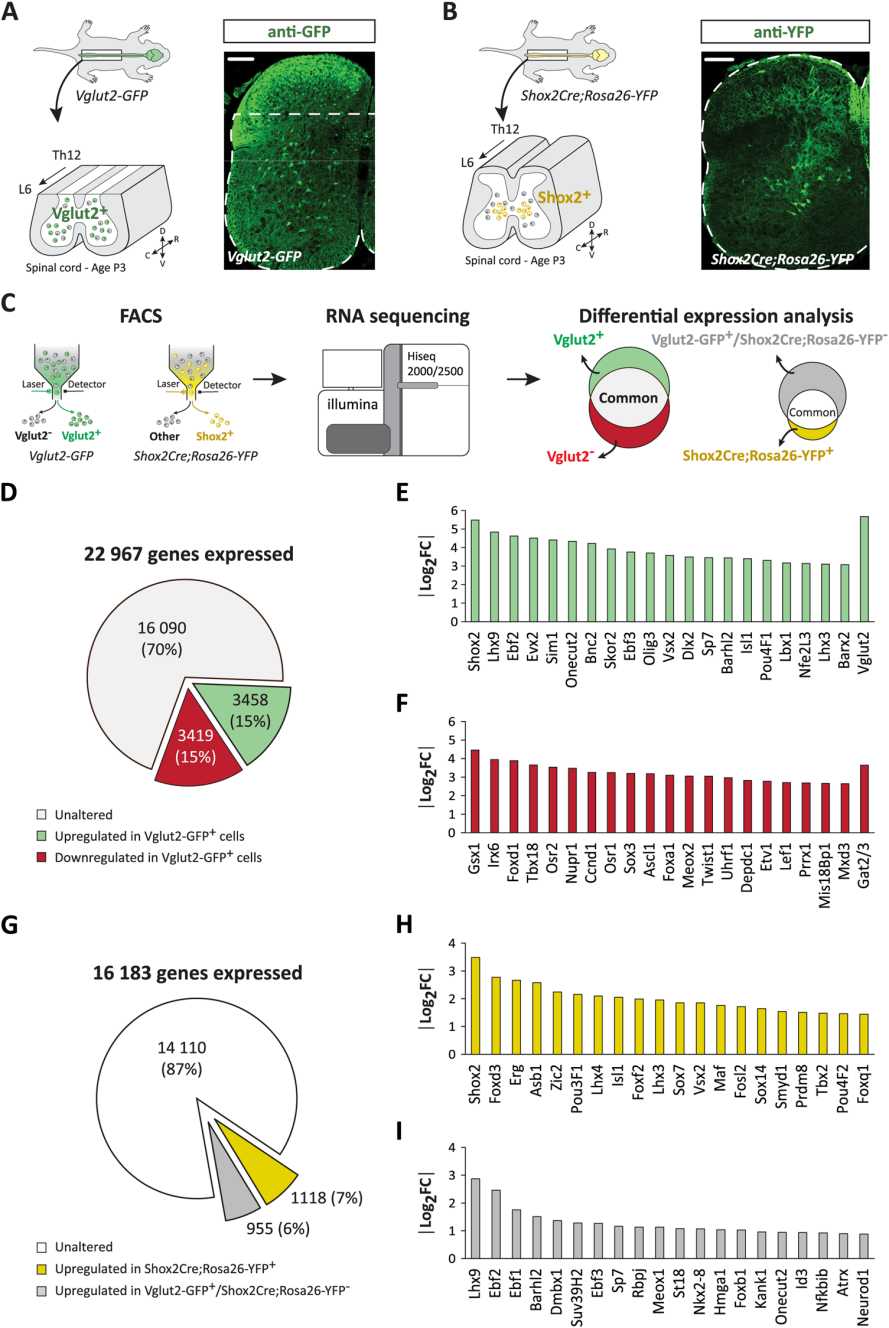
Genetic screen for excitatory molecular markers in the spinal cord. ***A,B***, Experimental schematic (*left panel*) and immunohistochemistry (*right panel*) showing the spatial distribution of the harvested GFP^+^ and YFP^+^ cells in the spinal cord (from thoracic segment 12 to lumbar segment 6) of *Vglut2-GFP* (lamina IV–X) (***A***) and *Shox2Cre;Rosa26-YFP* (***B***) mice (at P3), respectively. Dashed line on the section from the *Vglut2-GFP* mouse spinal cord mark the area from below which GFP spinal cells were isolated. Scale bars, 100 µm. D, dorsal; V, ventral; R, rostral, C, caudal. ***C***, Method outline and representation of differently expressed transcripts. FACS, fluorescence activated cells sorting; RNA, ribonucleic acid. ***D–F***, Pie chart of DESEq2 analysis. DESEq2 comparison of Vglut2-GFP^+^ and Vglut2-GFP^−^ transcriptomes revealed a total of 22,967 genes expressed (***D***), of which 3,458 were upregulated (red) and 3,419 down regulated (green) in Vglut2-GFP^+^ cells. Top 20 transcription factors upregulated (≥5.89-fold enrichment with *p*_adj_ ≤ 0.05) (including Vglut2 as a control) (***E***) and downregulated (≥4-fold downregulation with *p*_adj_ ≤ 0.05) (including with Gat2/3 as a control) (***F***) in Vglut2-GFP^+^ versus Vglut2-GFP^−^ spinal cells. ***G–I***, Pie chart of DESEq2 analysis. DESEq2 comparison of *Shox2Cre;Rosa26-YFP* and *Vglut2-GFP* transcriptomes revealed a total of 16,183 genes expressed in Shox2-YFP^+^ and Vglut2-GFP^+^ spinal cells (***G***), of which 1,118 were upregulated in Shox2-YFP^+^ cells (Shox2Cre;Rosa26-YFP^+^, yellow) and 955 were upregulated in Vglut2-GFP^+^ cells (Vglut2-GFP^+^/Shox2Cre;Rosa26-YFP^−^, gray). Top 20 transcription factors (all tissue) upregulated in Shox2^+^ spinal cells (≥2.87-fold enrichment with *p*_adj_ ≤ 0.05) (***H***) and upregulated in Vglut2-GFP^+^ spinal cells (***I***). Extended figures and data corresponding to this figure are included in Extended Data Figure 1-1 and 1-2 and Extended Data Table 1-1, 1-2, 1-3, and 1-4.

Spinal cords from newborn *Shox2Cre;Rosa26-YFP* and *Vglut2-GFP* mice at P3 were dissected in ice-cold (4°C) low Ca^2+^ Ringer's solution (in mM; 111.14 NaCl, 3.09 KCl, 11.10 glucose, 26 NaHCO_3_, 3.73 MgSO_4 _× 7H_2_O, 1.10 KH_2_PO_4_, 0.25 CaCl_2_) pH adjusted to 7.4 with 95% O_2_/5% CO_2_. The dorsal horn, lamina I–III, was removed from Vglut2-GFP spinal cords in order to guarantee that only intermediate and ventral GFP^+^ cells were isolated whereas intact spinal cords were used from *Shox2Cre;Rosa26-YFP* mice (Fig. 1*A,B*). Lumbar spinal segments — thoracic level 12 to lumbar level 6 (Th12-L6) — from individual mice were minced finely and subjected to a 15 min digestion at 37°C in a mix of Earle's Balanced Salt Solution (EBSS), DNAse I and papain according to the Papain Dissociation System manufacturer's instructions (Worthington Biochemical Corporation). Digested spinal cord tissue was passed through a cell strainer (35 µm nylon mesh, 12 × 75 mm style, BD Falcon), centrifuged (1,800 rpm) for 15 min at room temperature, and suspended in a mix of EBSS, albumin inhibitor (ovomucoid protease inhibitor and bovine serum albumin) and DNAse I according to manufacturer's recommendation (Worthington Biochemical Corporation). The cell suspension was passed through a discontinuous density gradient (albumin-inhibitor solution) and centrifuged (1,200 rpm) for 15 min at room temperature. The pellet of dissociated cells at the bottom of the tube was suspended in an ice-cold HBSS mix (1× HBSS, 1% Pen-Strep, 2% FBS, 20 mM D-glucose, 0.1% BSA, 0.001% DNAse I), and subsequently submitted for sorting.

**Figure 2.**
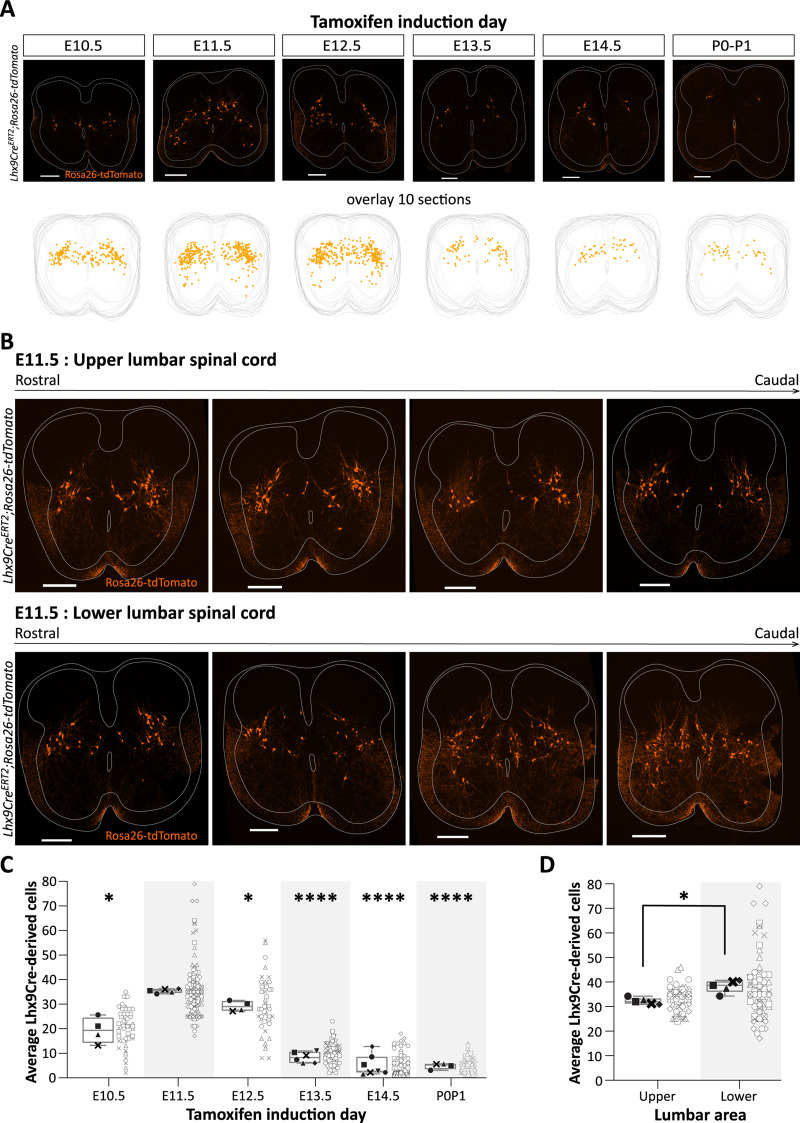
Spatiotemporal expression of Lhx9-derived neurons throughout the lumbar spinal cord. ***A***, Expression pattern of Lhx9-derived neurons at embryonic day E18.5 or postnatal day P5 after Cre-induction at different embryonic days (E10.5, E11.5, E12.5, E13.5, E14.5) or postnatal day P0/P1, respectively. *Upper panel*, Representative spinal cord section (20 µm thick) from each induction in *Lhx9Cre^ERT2^;Rosa26-tdTomato* neonatal mice (Lhx9-derived neurons in orange). Scale bar, 200 µm. *Lower panel*, Overlay of the position of Lhx9-derived neurons in 10 sections from each time point, each dot (orange) represents one Lhx9-derived neuron. ***B***, Expression pattern of Lhx9-derived neurons (in orange) in the lumbar spinal cord in *Lhx9Cre^ERT2^;Rosa26-tdTomato* neonatal mice after Cre-induction at E11.5. Scale bar, 200 µm. *Upper panel*, Distribution of Lhx9-derived neurons in the upper lumbar spinal cord (L1–L3, rostral to caudal). *Lower panel*, Distribution of Lhx9-derived neurons in the lower lumbar spinal cord (L4 to Sacral, rostral to caudal). ***C***, Quantification of Lhx9-derived cells at each tamoxifen induction day (*p = 0.0303 E11.5 vs E10.5, *p = 0.0168 E11.5 vs E12.5, ****p < 0.0001 for the rest, Brown–Forsythe and Welch ANOVA, B–F = 92.91_(5, 9.425)_ with *p* < 0.0001; *W* = 358.2_(5, 9.870)_ with *p* < 0.0001; Dunnett's multiple comparison to E11.5 time point). ***D***, Quantification of Lhx9-derived cells after induction at E11.5, upper lumbar (L1–L3) versus lower lumbar (L3–L6) (*p = 0.0255, two-tailed, paired *t* test, *t *= 3.473, df = 4). Graphs show min-to-max box plots with medians. Each marker (black) represents the mean of neurons per spinal cord with data from individual sections to the left (gray open dots).

10.1523/JNEUROSCI.1607-23.2024.f1-1 Figure 1-1 Section averaging for In Situ Sequencing (ISS) ISS on 29 sections from 5 spinal cords (P2). *Upper panel*: Description of spinal cord normalization. All spinal cords are normalized to the central canal (red X) and their maximal edge on each side (left, right, dorsal, ventral) to create a bounding box (square with red line) for each section for each spinal cord. This creates four areas, X_1_, X_2_ and Y_1_, Y_2_ which allows all cell positions to be normalized. This normalization allowed for an overlay of each section per transcript leading to the visualization of the local expression of each transcript within the spinal cord (see Figure 1-2). *Lower panel*: Sections from 5 different mice (5 to 4 sections for each animal) harvested at P2. Sections from the L2 spinal cord. Download Figure 1-1, TIF file.

10.1523/JNEUROSCI.1607-23.2024.f1-2 Figure 1-2 Spatial visualization of excitatory transcripts in the lumbar cord after ISS. A. Expression pattern of 16 excitatory transcripts in the lumbar cord (P2) after ISS. These transcripts have either a precise or a broad expression pattern. Shox2, Vsx2, Lhx3, Lhx4, Shox14, Islt1 and Zic2 represent the Shox2Cre;Rosa26-YFP^+^ population. The Vglut2-GFP^+^/Shox2Cre;Rosa26-YFP^-^ population is represented by Lhx9, Barhl2, Dmbx1, Neurod1, Onecut2 and St18 transcripts. Sim1 and Lbx1 represent other excitatory populations. Alpha values for the dots were 0.12 except for Slc17a6 which was 0.03. The n number represents the total number of transcripts found in the 29 sections from 5 different mice. Note that for all probes there is some background that leads to a spurious expression pattern. B. Overlay of the expression pattern of 4 excitatory transcripts: Barhl2 (yellow), Dmbx1 (dark grey), Lhx9 (magenta) and Neurod1 (light brown). These represent the Vglut2-GFP^+^/Shox2Cre;Rosa26-YFP^-^ population. All transcripts seem to have a unique expression pattern with little overlap with each other. The Lhx9 expression pattern of the transcript corresponds to the tdTomato expression seen in *Lhx9Cre^ERT2^;Rosa26-tdTomato* neonatal mice (see Figure 2). All probes present some background expression with outside the main expression area in the grey matter. Download Figure 1-2, TIF file.

10.1523/JNEUROSCI.1607-23.2024.t1-1 Table 1-1 **Differentially expressed transcription factors up-regulated in Vglut2-GFP^+^ cells (Vglut2-GFP^+^ *vs*. Vglut2-GFP^-^ analysis)** List of the differentially expressed transcription factors up-regulated in Vglut2-GFP^+^ cells. Download Table 1-1, DOCX file.

10.1523/JNEUROSCI.1607-23.2024.t1-2 Table 1-2 **Differentially expressed transcription factors down-regulated in Vglut2-GFP^+^ cells (Vglut2-GFP^+^ vs. Vglut2-GFP^-^ analysis)** List of the differentially expressed transcription factors down-regulated in Vglut2-GFP^+^ cells. Download Table 1-2, DOCX file.

10.1523/JNEUROSCI.1607-23.2024.t1-3 Table 1-3 **Differentially expressed transcription factors up-regulated in Shox2Cre;Rosa26-YFP^+^ cells (Vglut2-GFP^+^ *vs.* Shox2Cre;Rosa26-YFP^+^ analysis)** List of the differentially expressed transcription factors up-regulated in Shox2Cre;Rosa26-YFP^+^ cells. Download Table 1-3, DOCX file.

10.1523/JNEUROSCI.1607-23.2024.t1-4 Table 1-4 **Differentially expressed transcription factors up-regulated in Vglut2-GFP^+^/ Shox2Cre;Rosa26-YFP^-^ cells (Vglut2-GFP^+^ *vs.* Shox2Cre;Rosa26-YFP^+^ analysis)** List of the differentially expressed transcription factors up-regulated in Vglut2-GFP^+^/ Shox2Cre;Rosa26-YFP^-^ cells. Download Table 1-4, DOCX file.

GFP^+^, GFP^−^, and YFP^+^ cells were isolated by fluorescence activated cell sorting (FACS; 90 μM nozzle, 15–25 PSI, 1–2,000 EPS at 4°C) (FACSVantage Diva, Becton Dickinson; Karolinska Institutet core facility) from spinal cords dissociated from *Vglut2-GFP* and *Shox2Cre;Rosa26-YFP* mice, respectively. Sorted cells were collected in TRIsol LS reagent (Ambion, Life Technologies). Wild-type nonfluorescent littermate controls were used to set a collection gate, fluorescence spectra of collected cells, for each round of FACS. Success of collection was assessed by re-sorting of GFP^+^ and GFP^−^ cells.

### RNA extraction and cDNA amplification

The total RNA was isolated from flow sorted cells according to TRIsol LS reagent protocol's specifications (Ambion, Life Technologies). The RNA yield was measured on a Qubit Fluorometer (Qubit 2.0, Invitrogen) and a quality assessment was carried out on Agilent's 2200 TapeStation System (Agilent Technologies). RNA samples with RNA integrity number equivalent (RIN^e^) ≥8 were used for cDNA synthesis.

cDNA was synthesized from 16 ng of total RNA per sample using SMARTScribe RT technology (Chenchik et al., 1996) (SMARTer PCR cDNA Synthesis Kit, Clontech) and amplified over 20 cycles of long-distance PCR reaction (Advantage 2 PCR kit, Invitrogen). Amplified cDNA was purified using the NucleoSpin PCR Clean-up Kit (Macherey-Nagel) and eluted in NE buffer (5 mM Tris/HCl pH 8.5) according to manufacturer's instruction. Eluted cDNA was quantified on a Qubit Fluorometer (Qubit 2.0, Invitrogen) and submitted for library construction and sequencing at the Science for Life laboratory (SciLife).

### Library preparation and RNA-Seq

Library construction and sequencing were performed in the National Genomic Infrastructure (NGI) at the SciLife Lab. RNA-seq libraries were prepared with Illumina's TruSeq DNA PCR-Free (350 bp) for group 1 samples and TruSeq DNA (300 bp) library preparation kits on group 2 samples.

#### Group 1 samples

High-throughput RNA-seq was performed on 17 libraries (Vglut2-GFP^+^, *N* = 9 samples and Vglut2-GFP^−^, *N* = 8 samples; both collected from nine mice). All libraries were clustered on cBot and run with paired-end sequencing at 126 bp read length in one lane on the Illumina HiSeq 2500 System (HiSeq Control Software 2.2.58/RTA 1.18.64) using Illumina's Rapid High Output mode version 4 chemistry. Technical replicates were run three times under the same conditions.

#### Group 2 samples

A total of 12 cDNA samples (*Vglut2-GFP*, *N* = 7 samples collected from 14 mice and *Shox2Cre;Rosa26-YFP*, *N* = 5 samples collected from 10 mice) were submitted for library construction. All libraries were clustered on cBot and run with paired-end sequencing at 100 bp read length in two lanes, six libraries per lane, on the Illumina HiSeq 2000/2500 System using Illumina's Rapid High Output mode version 3 chemistry.

Sample demultiplexing and Bcl to Fastq conversion were performed using CASAVA bcl2Fastq v1.8.3 (group 1) and v1.8.2 (group 2). The quality scale used was Sanger/phred33/Illumina 1.8+.

### RNA-seq data analysis

Reads were mapped with Tophat/2.0.4 to the Mouse genome assembly, build GRCm38. BAM files from samples run on different lanes were merged with SAM tools. Merged BAM files were sorted, and duplicates removed using picard-tools/1.29. Gene counts were generated on Ensembl release 73 using HTSeq/0.6.1 (group 1) and HTSeq/0.5.1 (group 2) on BAM files with duplicates included.

Approximately 15 million uniquely mapped paired-end reads (14.46 ± 0.56 million) were obtained per sample in group 1, whereas 19 million uniquely mapped paired-end reads (18.71 ± 1.54 million) were obtained per sample in group 2.

### Differential expression analysis

To identify differentially expressed genes, we used a count-based statistical method, Bioconductor package DESEq2 (version 1.12.3) (Love et al., 2014), in the R statistical programming environment (R v3.3.1). The filter statistic in DESEq2 is the mean of normalized counts for a gene, while the test statistic is the *p*-value from the Wald test. Wald test *p*-values are adjusted for multiple testing using the procedure of Benjamini and Hochberg (1995).

Transcripts were considered expressed if they were assigned a valid *p*-value or adjusted *p*-value by DESEq2 (Love et al., 2014). According to DESEq2, transcripts were considered as not expressed if (a), within a row, all samples have zero counts; (b) a row contains a sample with an extreme count outlier, these outlier counts are detected by Cook's distance and can be refitted and replaced for conditions that contain seven or more replicates; and (c) a row is filtered by automatic independent filtering for having a low mean normalized count.

Transcripts were considered significantly differentially expressed if their adjusted *p*-value (*p*_adj_) were lower than 0.05 and their absolute log_2_ fold change (FC) higher than 0.59 (*p*_adj_ ≤ 0.05 and |log_2_FC| ≥ 0.59).

### Ingenuity pathway analysis (IPA)

The list of differentially expressed genes along with their relative *p*_adj_ and log_2_FC values were uploaded into QIAGEN's Ingenuity Pathway Analysis (IPA) software (http://www.ingenuity.com/), filtered for mice (species), in order to identify the most significant analysis-ready molecules associated with the dataset.

### In situ sequencing (ISS) for transcripts

Five spinal cords from newborn (age P2) wild-type mice were dissected in ice-cold (4°C) low Ca^2+^ Ringer's solution which was gassed with 95% O_2_/5% CO_2_. The spinal cords were quickly frozen in OCT (#4583, Sakura Tissue-Tek Europe) after carefully removing all Ringer solution. The tissues were cut in 10 µm thick sections from the lumbar (L) spinal cord (around L2) and kept at −80°C until further processed. To generate RNA expression profiles, we used the in situ sequencing (ISS) method as described by Ke et al. (2013) (https://doi-org.proxy.kib.ki.se/10.1038/nmeth.2563) on 29 sections from five different spinal cords. Probes for 16 genes were designed by Cartana AB. All assigned spots [analyzed as in Qian et al. (2020)] of the different genes for the different sections were normalized to the central canal (CC) as origo and a bounding box around the spinal cord (Fig. 1-1).

### Tissue processing and immunohistochemistry

#### Tissue processing

After the c-section, *Lhx9Cre^ERT2^;Rosa26-tdTomato* pups were checked for Cre-recombinase using a fluorescence lamp. Spinal cords and brains from embryonic stage E18.5 to P5 mice were first extracted in ice-cold (4°C) low Ca^2+^ Ringer's solution gassed with 95% O_2_/5% CO_2_. Then, spinal cords were fixed in 4% paraformaldehyde (PFA) overnight at 4°C and cryoprotected the next day in 30% sucrose in phosphate-buffered saline (PBS) overnight at 4°C. Next, the tissue was embedded in Tissue-Tek O.C.T compound (#4583, Sakura Finitek Europe) and preserved at −80°C for at least one night. Embedded tissues were sectioned with a cryostat (20 μm thickness) and mounted on SuperFrost Plus slides (ThermoFisher Scientific) or Epredia SuperFrost Plus Adhesion slides (#J1800AMNZ, Fisher Scientific). All slides were stored at −80°C until use.

#### Immunohistochemistry

For immunohistochemistry, sections were rehydrated for 3 × 15 min in PBS-0.5% Triton-X100 or PBS-0.1% Tween-20 (PBS-T) at room temperature (RT) and, then, blocked for 1 h in 1% normal goat serum and 1% normal donkey serum in PBS-T at RT. Sections were incubated with the primary antibody (prepared in blocking solution), overnight at 4°C. The following primary antibodies (1:1,000 µl) were used: rabbit polyclonal anti-RFP (#600-401-379S, Rockland); Living Colors DsRed Polyclonal Antibody (#632496, Takara); chicken anti-GFP (#ab13970, Abcam). Sections were washed for 3 × 15 min with PBS-T at RT and then incubated with the appropriate secondary antibodies (prepared in PBS-T) for 1 h at RT. The used secondary antibodies were: Cy3 AffiniPure Goat Anti-Rabbit IgG (H + L) (1:300; #111-165-003, Jackson ImmunoResearch); Alexa Fluor 488 anti-chicken (1:500; #A11039, Invitrogen); Alexa Fluor 568 anti-rabbit (1:200; #A10042, Invitrogen). Finally, slides were washed 3 × 15 min with PBS-T and either counterstained with NeuroTrace (1:200, 435/455 Blue Fluorescent Nissl Stain (#N21479) or 640/660 Deep-Red Fluorescent Nissl Stain (#N21483), ThermoFisher Scientific) and mounted in Prolong Diamond mounting media (#P3691, ThermoFisher Scientific) or mounted directly in Vectashield Plus Antifade mounting medium with DAPI (#H-2000, Vector Laboratories).

### RNAscope in situ hybridization

*Lhx9Cre^ERT2^;Rosa26-tdTomato* and *Lhx9Cre^ERT2^;Vglut2^Flx/Flx^* neonatal mice (E14.5 or E18.5) were sacrificed by decapitation. Spinal cords were dissected in ice-cold (4°C) low-Ca^2+^ Ringer's solution and immediately postfixed in 4% PFA, 4 h at RT (E14.5) or overnight at 4°C (E18.5). The following day, samples were cryoprotected, overnight at 4°C in 30% sucrose in PBS. Next, the tissue was embedded in Tissue-Tek O.C.T compound (#4583, Sakura Finitek Europe) and preserved at −80°C for at least one night. Transverse sections of the spinal cord were cut (16 µm) and collected using a cryostat and Superfrost Plus slides, respectively. The RNAscope Multiplex Fluorescent Reagent Kit v2 (Advanced Cell Diagnostics (ACD) Bio-techne) was used to perform the RNAscope in situ hybridization as well as the HybEZ oven for incubations. Slides were stored overnight at 4°C in 5× Saline Sodium Citrate after probes hybridization. The RNAscope in situ hybridization protocol was adapted to allow immunohistochemistry afterwards. The probes (all from ACD Bio-techne) used were: Vglut2 (Slc17a6-C2, catalog #319171-C2); VIAAT (Slc32a1-C1, catalog #319191); Chx10 (Vsx2-C1, catalog #438341); Shox2 (Shox2-O1-C3, catalog #554291-C3); Girk1 (Kncj3-C1, catalog #523951-C1); Girk2 (Kncj6-C3, catalog #472321-C3); Cre (CRE-C1, catalog #312281). To visualize the probes, Opal 520 and Opal 690 fluorophores (1:1,500, Akoya Biosciences) were used. After performing the RNAscope in situ hybridization, immunohistochemistry was performed using rabbit polyclonal anti-RFP (#600-401-379S, Rockland) as primary antibody, Alexa Fluor 568 anti-rabbit (#A10042, Invitrogen) as secondary antibody and counterstained with NeuroTrace 435/455 Blue Fluorescent Nissl Stain (#N21479, ThermoFisher Scientific) (see Immunohistochemistry section for protocol).

### Retrograde labeling

Spinal cord from *Lhx9Cre^ERT2^;Rosa26-tdTomato* pups (E18.5) were extracted in cold (4°C) low-Ca^2+^ Ringer's solution and an unilateral cut on the ventral side of the cord was made either at the lumbar level 2 (L2) or at L4 (Stokke et al., 2002). Neurons were then labeled retrogradely by applying crystals of Biotin-dextran-amines (Dextran, Biotin 3,000 MW Lysine Fixable, #D7135, Invitrogen) into the previously made cut. Next, the preparations were incubated in an oxygenated (95% O_2_/5% CO_2_) normal Ringer's solution (in mM, 111.14 NaCl, 3.09 KCl, 11.10 glucose, 26 NaHCO_3_, 1.26 MgSO_4 _× 7H_2_O, 1.10 KH_2_PO_4_, 1.85 CaCl_2_) at room temperature for 8–16 h before immersion in 4% PFA (see Tissue processing protocol). For immunochemistry, in addition to the primary antibody (rabbit anti-RFP), samples were incubated with Streptavidin-Alexa Fluor 488 conjugate (1:300; #S11223, Invitrogen).

### Imaging, cells count and normalization for cell visualization

Imaging of the entire transverse or hemi sections were done using a confocal microscope (LSM 800 or LSM 900, Zeiss) either with a 10× or a 20× objective. For zoomed images, a 20× objective was used accompanied by a *z*-stack acquisition to allow better quantification. All cells were manually counted for the entire transverse or hemi section using a cell-counter plugging in ImageJ or Fiji. Shapes of the spinal cords were also manually extracted using the cell-counter plugging in ImageJ. All cells were normalized to the CC and plotted using a custom-made script using the python language to assess their spatial location.

### Electrophysiology experiments

#### Dissection

Mice (P0–P3) were decapitated and spinal cords were extracted in ice-cold (4°C) and oxygenated (95% O_2_/5% CO_2_) low-Ca^2+^ Ringer's solution. After dissection, the free spinal cords were transferred to the recording chamber (ventral side up) and mounted on a Zeiss Axioskop 2 microscope equipped with fluorescent filters. Normal Ringer's solution was applied at a rate of approximately 4 mL/min.

#### Drugs

N-methyl-D-aspartate (NMDA; 2–3, 6 or 9–10 µM) was used in combination with 5-hydroxytryptamine (5-HT; 8 µM) or 5-HT only (3 or 8 µM). The variable concentration of NMDA allowed to induce locomotor-activity at different frequencies. For acute inhibition experiments, clozapine N-oxide (CNO, Merck) was first dissolved in 0.9% Saline to obtain a stock solution of 1 mg/mL and then diluted in a normal Ringer's solution mixed with NMDA and 5HT to obtain a final concentration of 10 µM.

#### Recording of locomotor activity

Suction electrodes were placed on the left and right side of the lumbar (L) spinal cord at the level of segment 2 (L2) and 5 (L5). L2 activity corresponds to flexor while L5 activity corresponds to extensor activity (Kjaerulff and Kiehn, 1996). The signals were band-passed filtered (100Hz–1 kHz), amplified 10,000-fold and sampled at 10 kHz using pClamp software (Clampex v.10, Molecular Devices). For all experiments, recordings were done for at least 15–30 min after the initial burst of drug-induced activity in order to reach a stable locomotor activity using NMDA and 5-HT. For acute inactivation experiments, CNO was added to the Ringer solution when stable locomotor activity was obtained and was perfused for a duration of 15–25 min. All recordings were performed at RT (20–22°C). In most cases, the experimenter was blind to the genotype that was determined postexperimentally.

### Optogenetic experiments: light stimulation

For acute activation of Lhx9-derived neurons, spinal cord from *Lhx9Cre^ERT2^;ChR2-EYFP* pups (P0–P3) were used. Broad stimulation of the cord was performed using a fluorescence light source (Zeiss HXP 120) which was first filtered with a band-pass filter (excitation: 470–490 nm; emission: 520–560 nm; blue light) and then passed through a 2.5× objective (Zeiss microscope) to target the ventral lumbar cord (L1–L6). Light stimulation was 30 s. For local stimulation of the lumbar cord (either L1/L2 or L5/L6), a 473 nm laser system (UGA-40; Rapp Optoelectronic) was used. This system delivered a blue light at an intensity up to 30 mW/mm^2^ and was directed at the preparation using an optical fiber (200 μm core, 0.22 NA, Thorlabs) (Hägglund et al. (2013)). Light stimulation was 20 s. Two to three trials were tested for each preparation and for each stimulation.

### Analysis of locomotor activity

Analyses were done from recordings >25 min (min) after the initial burst of drug-induced activity except for the acute silencing experiments (see below). For chronic silencing experiments, analyses were done using a 5 min window between 25 and 30 min. For optogenetic experiments, analyses were performed with a 20 s window for the baseline and immediately after the light was switched off (post-light). For the light stimulation, the window duration used for analyses varied (either 20 s or 30 s) depending on the light stimulation protocol (see above). For acute silencing experiments, locomotor frequency was averaged over 2 min windows to obtain frequency bins over time. Baseline frequency was determined for the last 4 min before CNO application and compared to the last 4 min during CNO application before washout (>15–20 min after the start of each condition (baseline, CNO); 4 min window). Analyses were conducted either manually using Spike2 software (v7.20, Cambridge Electronic Design) or using a custom-made script implemented in the Python programming language to find the peaks, valleys, beginning and end of each burst. Main parameters of the locomotor activity (frequency, burst duration, duty cycle, amplitude) and their coefficient of variation were calculated using either Excel or a custom-made script implemented in the Python programming language. In general, flexor (L2) ventral roots were used to calculate all locomotor parameters except for the lower lumbar stimulation in the optogenetic experiments where extensor (L5) ventral roots were used. The average frequency was defined as the number of burst found divided by the duration of the analyzed window; the burst duration was defined as the time difference between the end of the burst and the beginning of the burst; the cycle period was defined as 1 divided by the frequency; the duty cycle was defined as the burst duration divided by the cycle period; the amplitude as the difference between the peak amplitude and the valley amplitude for each burst. For each parameter, the coefficient of variation was calculated by dividing the standard deviation of the sample for the parameter by the mean of the parameter. And finally, for each parameter, the mean per preparation was plotted. For optogenetic experiments, the data plotted represents the mean of two to three trials per spinal cord.

The phase relationship between left–right and flexor–extensor of the ventral roots were analyzed using the SpinalCore software (Mor and Lev-Tov, 2007) and mean angle and circular plots were generated using a custom-made script implemented in the Python programming language.

### Calcium imaging recording and analysis

For visualizing activity in Lhx9-derived cells, spinal cord from *Lhx9Cre^ERT2^;Gcamp6f* pups (P0–P3) were used. Spinal cords were transversally cut at the level of L2/L3 or the level L4/L5 and mounted in the recording chamber with a 90° angle to face the objective (Hsu et al. (2023)). A 10× objective was used to record calcium-activity of the entire transverse section. Suction electrodes were used to record the ventral roots activity on both side of the spinal cord at the same level as the cut when possible or at a more rostral lumbar level than the transverse cut. A fluorescence light source (Zeiss HXP 120) with a band pass filter (excitation: 470–490 nm; emission: 520–560 nm; blue light) was used to shine light onto the transverse section. Activity-dependent changes in fluorescence were detected using a digital CMOS camera (PCO edge 5.5, Germany) at 10 frames/s and stored directly on the computer. Calcium data was collected with Camware (PCO). All recordings were done (duration: 3 min) at least 25 min after the initial burst of drug-induced activity. Changes in fluorescence were extracted offline using the image processing software, ImageJ. First, a normalization of the imaged recording field was performed. In brief, the four edges of the spinal cord should meet the border of the field with the CC at the midline to 500 × 400 pixels. Then, changes in fluorescence intensity over time for the normalized field were converted to Δ*F* = *F_t_* − *F*_0_ where *F_t_* is the fluorescence at any specific time *t* and *F*_0_ is the baseline fluorescence (averaged value of last 100 frames). We generated 500 grid regions of interests (ROIs) that cover the entire normalized field. Δ*F* variations over time were extracted for each ROI, normalized by the maximum Δ*F* value of all ROIs. For analyzing the oscillation strength of the rhythmic Ca^2+^ activity for each of the ROIs, a low-pass filter was used and an adjustment of the baseline of the Ca^2+^ traces was performed. Then an autocorrelation analysis was carried out by pClamp software (Clampfit v10.7, Molecular Devices), to evaluate the degree of regularity of rhythmicity. An autocorrelation of +1 represents a perfect positive correlation, while an autocorrelation of negative 1 represents a perfect negative correlation. The oscillation index was calculated as the value of the peak to the trough correlation coefficient. This value reflects the consistency and strength of the oscillatory activity. The larger the oscillation index, the stronger the rhythm is. To normalize the index for reconstructing the physical map of the oscillation index, we set the maximum value of 500 grids as the maximum for both the right (1–250 grid) and left (251–500 grid) halves of the spinal cord. Oscillation index maps were generated for each individual preparation, and an averaged map was subsequently generated to assess group data. The ROI with the highest oscillation index of the averaged map was defined as the reference ROI, which was used in cross-correlation analysis (Clampfit v10.7, Molecular Devices). Cross-correlation between the individual ROIs and the reference ROI oscillation was used to reveal the phase relationship between individual ROIs and the reference ROI. The phase value of individual ROIs was calculated, and the value was presented by the locomotor cycle. Phase maps were generated for individual preparations and then averaged for quantification purposes.

### Statistics

All data were analyzed with GraphPad Prism (v9.5.1), except for the circular plots analyses which were done with the Python package pycircstat (https://github.com/circstat/pycircstat).

For all graphs (except circular plots), normal distribution was checked using the Shapiro–Wilk normality test. If all groups under different conditions (i.e., different NMDA concentrations) followed the normal distribution then a parametric test was used. If one of the groups did not follow the normal distribution, a nonparametric test was used.

For two-group comparisons, two-tailed Student's *t* test (paired or unpaired as appropriate) was used. For unpaired *t* test, when normality was met, Welch's correction was used (the *t* distribution value (*t*) and the degree of freedom (df) were reported) and when normality was not met, Mann–Whitney *U* test was used. For paired *t* test, when normality was met, a normal paired *t* test was used (*t* and df were reported). When normality was not met, Wilcoxon's matched-pairs signed rank test was used (the sum of signed ranks (*W*) was reported).

For three-group or more comparisons, one-way ANOVA or one-way ANOVA repeated measures (RM) was used when appropriate. For one-way ANOVA, when normality was met, Welch's (W) and Brown–Forsythe (B–F) corrections were used, and multiple comparisons were made with Dunnett's. For one-way ANOVA-RM, when normality was met, Geisser–Greenhouse correction was used followed by multiple comparisons with Turkey's test.

All means ± SD presented in the text were rounded to two decimals. All graphs represent either mean with ± SD or min-to-max boxplots (box extends from the 25th to 75th percentiles, line in the middle represents the median).

Circular statistics represent the phase relationship between ipsilateral flexor (L2)-extensor (L5) and between left and right side of the spinal cord. In general, the mean angle represents the preferred phase of the activity, and the length of the vector (*r*) indicates the concentration of phase values around the mean. If the phase values are highly concentrated around the mean phases, there is a strong coupling between left–right or flexor–extensor. To test the strength of the coupling (uniformity) within the same group, Rayleigh's test was performed (using *r* values of each preparation) and coupling was considered significant if *p *< 0.05. To compare two or more groups, Watson-William's test was used, and statistical difference is reached if *p *< 0.05.

The mean angle for each preparation and the grand mean angle (overall mean) were plotted using circular plots where 0° correspond to a synchronous locomotor-like activity as compared to 180° which corresponds to an alternating locomotor-like activity either between ipsilateral flexor–extensor or left–right side of the lumbar cord.

## Code accessibility

Codes used for analysis and/or statistics are available from the corresponding author upon reasonable request.

## Results

### Transcriptional analysis of spinal glutamatergic neurons

To identify new molecular markers for excitatory subpopulations involved in the locomotor network we extracted samples from the ventral-most part of the spinal cord where the locomotor network is located (Fig. 1*A,B*). We first compared the transcriptome between spinal glutamatergic and non-glutamatergic cells. The glutamatergic and non-glutamatergic cells were defined by the presence or absence of GFP expression in *Vglut2-GFP* mice (Fig. 1*C*). Vglut2 is the main vesicular glutamate transporter in the spinal cord (Oliveira et al., 2003; Landry et al., 2004; Gezelius et al., 2006; Wallén-Mackenzie et al., 2006) and is expressed in locomotor-related interneurons in the spinal cord (Kiehn et al., 2008; Hägglund et al., 2010, 2013). We then made stepwise differential expression analyses of the transcriptome of the different ventral excitatory subpopulations. We hypothesized that a comparison of the transcriptome of the main glutamatergic population in the ventral spinal cord with one of the glutamatergic subpopulations of neurons that has been linked to rhythm generation could reveal molecular markers that fractionate the parental Vglut2 population and thereby shed some light on putative candidates for rhythm generation while excluding generic excitatory markers. Therefore, we compared the transcriptome between glutamatergic cells as defined by GFP expression in *Vglut2-GFP* mice at postnatal day 3 (P3) and Shox2 interneurons as defined by YFP expression in *Shox2Cre;Rosa26-YFP* mice (P3) (Fig. 1*A–C*). The Shox2 population contains rhythm generating neurons that are negative for Chx10 and neurons that modulate the burst amplitude which are positive for Chx10. However, the comparison to all the Vglut2^+^ neurons, should potentially identify new population(s) of rhythm generating neurons.

All viable GFP^+^ and YFP^+^ cells were collected by fluorescence activated cell sorting (FACS) from postnatal day 3 (P3) lumbar spinal cords (from thoracic segment 12 to lumbar segment 6) dissociated from *Vglut2-GFP* and *Shox2Cre;Rosa26-YFP* mice, respectively (Fig. 1*C*). We isolated an equivalent number of non-glutamatergic cells (GFP^−^) from the *Vglut2-GFP* spinal cords (35,770 GFP^+^ vs 38,117 GFP^−^). On average, GFP^+^ cells corresponded to less than 10% (35,770/427,866) of all viable cells collected from *Vglut2-GFP* spinal cords, whereas YFP^+^ cells corresponded to less than 1% (4,580/558,524) of all viable cells collected from each *Shox2Cre;Rosa26-YFP* spinal cord. RNA sequencing was performed on mRNA isolated from sorted GFP^+^, GFP^−^ and YFP^+^ cells (Fig. 1*C* and Materials and Methods). We used DESEq2 (Love et al., 2014) on normalized mean raw read counts in order to identify genes that were differentially expressed between cells (Fig. 1*C*). Genes were considered differentially expressed if their adjusted *p*-value (*p*_adj_) was lower than 0.05 and their absolute log2 fold change (FC) was higher than 0.59 (|log2FC Vglut2-GFP^+^ vs Vglut2-GFP^−^| ≥ 0.59; |log2FC Vglut2-GFP vs Shox2Cre;Rosa26-YFP| ≥ 0.59; *p*_adj_ ≤ 0.05).

#### Transcripts up- and downregulated in Vglut2-GFP^+^ cells

The differential expression analysis using DESEq2 analysis between glutamatergic and non-glutamatergic cells revealed that of the 22,967 transcripts expressed in Vglut2-GFP^+^ spinal cells, 3,458 (15%) transcripts were upregulated (log2FC ≥ 0.59; *p*_adj_ ≤ 0.05), and 3,419 (15%) transcripts were downregulated (log2FC ≤ −0.59; *p*_adj_ ≤ 0.05) in Vglut2-GFP^+^ cells as compared to the Vglut2-GFP^−^ cells (Fig. 1*D*). Ingenuity Pathway Analysis (IPA) returned 8,264 analysis-ready molecules as the most biologically relevant molecules. As an internal validation of our analysis, we first verified that vesicular glutamate transporter 2 (Vglut2) was the main upregulated neurotransmitter transporter and that the GABA transporter 2/3 (Gat2/3) was the main downregulated transporter in Vglut2-GFP^+^ cells (Fig. 1*E,F*).

We focused our analysis on transcription factors (TFs) because of their roles in specifying neuronal subtypes in the spinal cord (Jessell, 2000; Goulding and Pfaff, 2005).

#### Differential expression of transcription factors

From the 450 differentially expressed TFs identified with IPA, 185 were upregulated (Extended Data Table 1-1), and 265 were downregulated (Extended Data Table 1-2) in Vglut2-GFP^+^ cells.

Amongst the top 20 TFs upregulated in Vglut2-GFP^+^ cells, we find transcripts for transcription factors that are known to be expressed in ventral spinal neurons (Fig. 1*E* and Extended Data Table 1-1; Vglut2 is included in the plot as a control of cells being glutamatergic). The TFs that are upregulated are Sim1, Evx2, Lhx3, Vsx2, Isl1, Lbx1, and Shox2 (Fig. 1*E*). Sim1 is the marker for V3 neurons, which comprise a major group of excitatory commissural and ipsilateral neurons in the ventral spinal cord (Zhang et al., 2008; Borowska et al., 2013, 2015; Chopek et al., 2018; Danner et al., 2019). Evx2, along with Evx1, determines the glutamatergic fate of the commissural spinal V0_V_ neurons (Moran-Rivard et al., 2001; Juárez-Morales et al., 2017) which are involved in left–right coordination (Talpalar et al., 2013; Bellardita and Kiehn, 2015). Vsx2 (Chx10) and Lhx3 delineated, the V2a neurons (Crone et al., 2008, 2009; Dougherty and Kiehn, 2010; Zhong et al., 2010, 2011; Talpalar et al., 2013) that are ipsilaterally projecting glutamatergic neurons (Al-Mosawie et al., 2007; Lundfald et al., 2007). In addition to ventral-domain derived neurons, we also found markers for dorsal-domain derived glutamatergic neurons that settle in the ventral spinal cord (Fig. 1*E*). Isl1 is expressed in neurons that are dI3 derived but define a class of glutamatergic premotor interneurons in the medioventral spinal cord (Liem et al., 1997; Helms and Johnson, 2003; Bui et al., 2013). Lbx1 (Fig. 1*E*) and Lmx1b (Extended Data Table 1-1) glutamatergic neurons that are derived from the dI5 domain and that settle both in the deep dorsal and the intermediate area (Mizuguchi et al., 2006; Lu et al., 2015; Hilinski et al., 2016). Finally, Shox2 was the top differentially expressed TF upregulated in Vglut2-GFP^+^ cells (Fig. 1*E*). Shox2 cells are derived from the ventral domain V2 as well as dorsal domain dI3 and dI5 (Dougherty et al., 2013; Ha and Dougherty, 2018). Other TFs that were found among the top 20 TFs are Lhx9, Ebf2, Onecut2, Bnc2, Skor2, Ebf3, Olig3, Dlx2, Sp7, Barhl2, Pou4F1, Nfe2L3, and Barx2.

Amongst the top 20 TFs downregulated in Vglut2-GFP^+^ (Fig. 1*F*; Gat2/3 included as an internal control), we identified among others, TFs involved in vascular development such as Meox2 (Douville et al., 2011), and in establishing a mesenchymal cell phenotype such as Twist1 (Katoh and Katoh, 2009). Gata2/3, markers for the ventral inhibitory interneuron group V2b, were also identified as one of the downregulated transcripts in Vglut2-GFP^+^ cells (Zhang et al., 2015; Extended Data Table 1-2). Additionally, we found genes reported to act as both glial and neuronal progenitors such as Ascl1, which together with Gsx1 has also been linked to neuronal fate determination (Mizuguchi et al., 2006; Vue et al., 2014).

#### Transcripts differentially expressed between Vglut2-GFP^+^ and Shox2-YFP^+^ spinal cells

We next compared the transcriptomic profile of Vglut2-GFP^+^ and Shox2-YFP^+^ spinal cells. To ensure the purity of the analysis, transcripts that were identified as downregulated in glutamatergic cells by the Vglut2-GFP^+^ versus Vglut2-GFP^−^ analysis were pre-emptively subtracted from the Vglut2-GFP^+^ versus Shox2Cre;Rosa26-YFP^+^ differential analysis. This new differential analysis defines two populations, Shox2Cre;Rosa26-YFP^+^ cells and Vglut2-GFP^+^/Shox2Cre;Rosa26-YFP^−^ cells (Fig. 1*G*).

The DESEq2 analysis revealed that of the 16,183 genes expressed in Shox2Cre;Rosa26-YFP^+^ and in Vglut2-GFP^+^ spinal cells, 1,118 (7%) were enriched in Shox2Cre;Rosa26-YFP^+^ cells and 955 (6%) were enriched in Vglut2-GFP^+^/Shox2Cre;Rosa26-YFP^−^ cells (Fig. 1*G*).

Of the 2,073 differentially expressed transcripts, IPA identified 1,042 of them as biologically significant analysis-ready molecules for the mouse. Among these we focused further on differentially expressed TFs. From the 109 differentially expressed TFs identified with IPA, 62 of them were upregulated in Shox2Cre;Rosa26-YFP^+^ cells (Fig. 1*H* and Extended Data Table 1-3), and 47 of them were upregulated in Vglut2-GFP^+^/Shox2Cre;Rosa26-YFP^−^ cells (Fig. 1*I* and Extended Data Table 1-4).

Shox2 was the main differentially expressed TF upregulated in Shox2Cre;Rosa26-YFP^+^ cells, a clear indicator that we have successfully isolated the Shox2 neuron population (Fig. 1*H*). Moreover, amongst the top 20 TFs enriched in Shox2Cre;Rosa26-YFP^+^ cells, we also identified markers that are known to define subgroups of Shox2 neurons namely Isl1, Lhx3, Vsx2 (Chx10), Sox14 (Crone et al., 2008; Dougherty et al., 2013), and Maf (Delile et al., 2019; Fig. 1*H*). The remaining TFs found enriched in Shox2Cre;Rosa26-YFP^+^ cells are Foxd3, Erg, Asb1, Zic2, Pou3F1, Lhx4, Foxf2, Sox7, Fosl2, Smyd1, Prdm8, Tbx2, Pou4F2, and Foxq1 (Fig. 1*H*).

In the Vglut2-GFP^+^/Shox2Cre;Rosa26-YFP^−^ spinal cells, we found that Lhx9 is the main differentially upregulated TF followed by other TFs such as Ebf2, Ebf1, Barhl2, Dmbx1, Suv39H2, Ebf3, Sp7, Rbpj, Meox1, St18, Nkx2-8, Hmga1, Foxb1, Kank1, Onecut2, Id3, Nfkbib, Atrx, and Neurod1 (Fig. 1*I*).

Collectively these data identify TFs that appear to fractionate the Shox2Cre;Rosa26-YFP^+^ and Vglut2-GFP^+^/Shox2Cre;Rosa26-YFP^−^ cell populations.

To further divide these transcripts into subpopulations, we investigated their expression pattern in the spinal cord at P2. For this, we used ISS (Ke et al., 2013) experiments for transcripts of the Shox2Cre;Rosa26-YFP^+^ population (Shox2, Vsx2, Lhx3, Lhx4, Shox14, Isl1, Zic2), the Vglut2-GFP^+^/Shox2Cre;Rosa26-YFP^−^ population (Lhx9, Barhl2, Dmbx1, Neurod1, Onecut2, St18) and other excitatory populations (Sim1, Lbx1) (Extended Data Fig. 1-2A). These analyses showed restricted patterns of expression for several of the transcripts while others have a more widespread pattern. Particularly, it showed that Dmbx1, Neurod1, Bahrl2 and Lhx9 have a localized expression in the intermediate part of the spinal cord where locomotor networks are localized (Fig. 1–2*B*).

Given that the Lhx9 transcript was found to be highly expressed in both differential expression analysis (Vglut2-GFP^+^ vs Vglut2-GFP^−^ and the Vglut2-GFP^+^ vs Shox2Cre;Rosa26-YFP^+^), we set out to characterize the role of the Lhx9-expressing cells in the spinal locomotor network.

### Lhx9-derived neurons depict an excitatory population with ipsilateral axonal projections

To functionally characterize cells expressing Lhx9, we first investigated the temporal and spatial expression of Lhx9-derived cells in the lumbar spinal cord. By crossing a conditional *Lhx9Cre^ERT2^* mouse line (Balasubramanian et al., 2014) with the *Rosa26-tdTomato* reporter mouse line, Lhx9-recombined cells were labeled and defined by the presence of Rosa26-tdTomato expression. Tamoxifen (Stock solution: 5 mg/mL; dose: 0.1mL/20g body weight) was given orally in pregnant mice on days corresponding to different embryonic time points (Embryonic day, E10.5–E14.5) to induce labeling of Lhx9-derived neurons (Fig. 2*A*) and the expression pattern was determined at E18.5. In a few mice, tamoxifen was given orally at postnatal day 0 and 1 (P0/P1) and the expression pattern was determined at P5. Lhx9-derived cells were highly expressed after induction at embryonic day E11.5, while induction at earlier or later time points lead to labeling of fewer neurons postnatally [mean cell number (mean ± SD for all); E10.5 = 19.40 ± 5.26, *N* = 4 spinal cords, *n* = 19/10/14/10 sections per spinal cord; E11.5 = 35.44 ± 0.83, *N* = 5, *n* = 32/23/40/20/20; E12.5 = 29.18 ± 2.04, *N* = 4, *n* = 12/12/11/11; E13.5 = 8.36 ± 2.14, *N* = 6, *n* = 29/28/27/41/40/40; E14.5 = 5.07 ± 4.18, *N* = 7, *n* = 28/25/26/24/12/12/12; P0P1 = 4.83 ± 1.21, *N* = 4, *n* = 28/28/33/33] (Fig. 2*A,C*). The Lhx9-derived cells were distributed throughout the entire length of the lumbar spinal cord (Fig. 2*B*) and were colocalized with the neuronal marker NeuN [97.56 ± 1.73% (mean ± SD), *n* = 9 sections]. These neurons were present laterally in the dorsal part of the intermediate lamina of the lumbar spinal cord and extending into the deep dorsal horn (Fig. 2*B*) as well as around the CC in the lower part of the lumbar spinal cord (Fig. 2*B*, lower panel). The tdTomato expression pattern overlaps visually with the Lhx9-expression pattern (Extended Data Fig. 1-1, 1-2). There was minor difference in the number of Lhx9-derived neurons expressed in the upper (segment 1–3) versus lower (segment 3–6) lumbar spinal cord [mean cell number (mean ± SD for all), upper lumbar spinal cord = 32.23 ± 1.37 (*N* = 5 spinal cords, *n* = 14/11/21/9/9 sections per spinal cord versus lower lumbar spinal cord = 38.26 ± 2.55 (*N* = 5, *n* = 18/12/19/11/11)] (*p = 0.0255, two-tailed, paired *t* test, *t *= 3.473, df = 4) (Fig. 2*D*). Since the peak of Lhx9-derived neurons appeared by Cre induction at E11.5, we used this time point for all functional studies.

**Figure 3.**
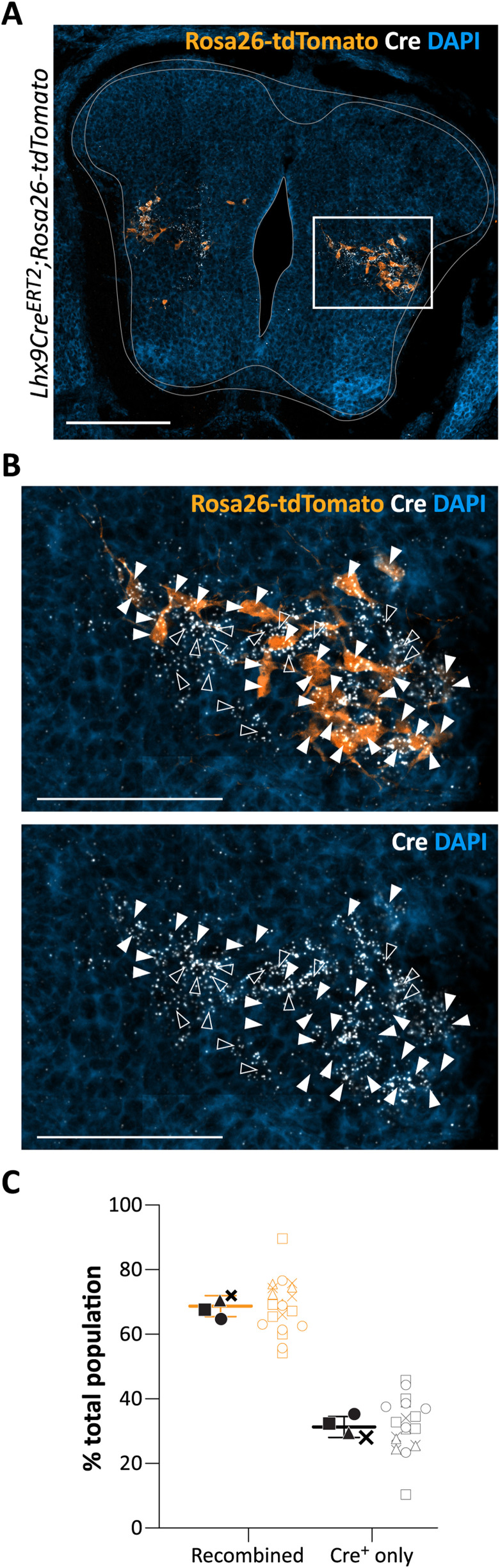
Quantification of the level of recombination in *Lhx9Cre^ERT2^;Rosa26-tdTomato mice*. ***A***, Transverse section at the lumbar level showing RNAscope in situ hybridization for Cre (in white) and immunolabeling of Lhx9-derived neurons (in orange) and DAPI (in blue) in spinal cords from *Lhx9Cre^ERT2^;Rosa26-tdTomato* E14.5 embryos. ***B***, Enlargement of the white boxed area in (***A***). Scale bar for the transverse section, 200 µm; magnified boxed, 100 µm. Filled white arrows point to the recombined cells (tdTomato^+^). Empty white arrows point to cells which are not recombined (corresponding to tdTomato^−^/Cre^+^ cells). ***C***, Quantification (in percent of total population) of recombined cells (tdT^+^) and nonrecombined cells (Cre^+^ only) in hemi sections of the lumbar spinal cord from *Lhx9Cre^ERT2^;Rosa26-tdTomato* E14.5 embryos. Graph represent the means per animal to the right with data from individual sections to the left.

To evaluate the level of recombination created by a single induction of tamoxifen at E11.5, we used RNAscope in situ hybridization to visualize the Cre in Lhx9-derived cell combined with immunohistochemistry against tdTomato in tissue harvested at embryonic day 14.5 (E14.5). Cells that have recombined express tdTomato (tdT^+^) (some of these cells showed low level of Cre possible as consequence of Lhx9 downregulation early in development) while nonrecombined cells are characterized by Cre expression alone. Like at E18.5, we found that the majority of the tdTomato^+^ cells are located in the lateral intermediate area with a smaller population of cells close to the CC (Fig. 3*A,B*). The quantification of Cre and tdTomato cells showed that on average 68.70 ± 3.24% (mean ± SD) of the total population recombined (*N* = 4 spinal cords, *n* = 6/6/5/4 sections per spinal cord) (Fig. 3*C*). A single dose of tamoxifen therefore led to recombination in the majority although not all Lhx9Cre cells.

**Figure 4.**
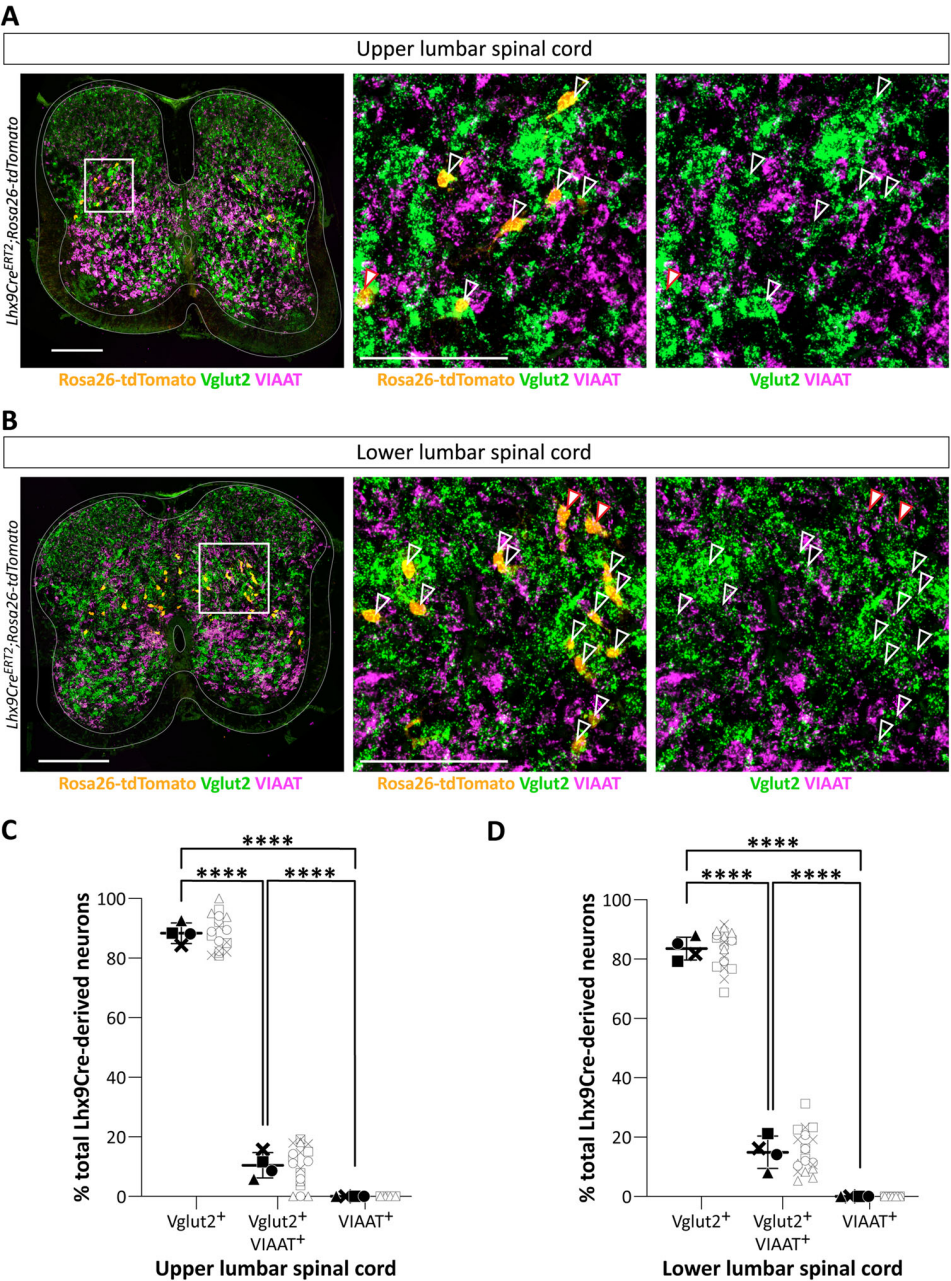
Lhx9-derived neurons are excitatory. ***A,B***, Transverse section of the lumbar spinal cord at the upper lumbar (***A***) or at the lower lumber level (***B***) showing RNAscope in situ hybridization for Vglut2 (*Slc17a6*, in green) and VIAAT (*Slc32a1*, in magenta) and immunolabeling of Lhx9-derived neurons (in orange) in *Lhx9Cre^ERT2^;Rosa26-tdTomato* neonatal mice (E18.5). Rightmost pictures are magnifications of the white boxed areas. Scale bar for the full transverse section, 200 µm; magnified box, 100 µm. Empty white arrows point to Lhx9-derived neurons that are colocalized with Vglut2. Filled white arrows with red line point to Lhx9-derived neurons that are colocalized with Vglut2 and VIAAT. ***C,D***, Quantification (in percent of total Lhx9-derived neurons) of entire transverse sections in the upper lumbar (***C***) and lower lumbar (***D***) (for both graph, ****p < 0.0001, One-way RM ANOVA with Geisser–Greenhouse correction (upper lumbar spinal cord, *F*_(1,035, 18,64) _= 1,149 with *p *< 0.0001; lower lumbar spinal cord, *F*_(1,025, 19,47) _= 936.2 with *p *< 0.0001), Turkey's multiple comparison). For all graphs, mean ± SD. Each marker (black) represents the mean % of neurons per spinal cord with data from individual sections to the left (gray open dots).

We next went on to determine the neurotransmitter phenotype of the Lhx9-derived neurons (Fig. 4). For this, we used *Lhx9Cre^ERT2^;Rosa26tdTomato* neonatal mice and performed RNAscope in situ hybridization followed by an immunohistochemistry against tdTomato to label Lhx9-derived neurons.

For transmitter phenotyping, we used the Vglut2 (*Slc17a6* probe) to label glutamatergic neuron and the vesicular amino acid transport (VIAAT; *Slc32a1* probe) to label inhibitory neurons expressing either γ-aminobutyric (GABA) and/or glycine (Fig. 4*A,B*). We found that the majority of tdTomato^+^ neurons express only Vglut2 while a small fraction of this population co-express Vglut2 and VIAAT in the lumbar cord [(mean ± SD for all), upper lumbar: Vglut2^+ ^= 88.11 ± 5.87% and Vglut2^+^/VIAAT^+ ^= 10.70 ± 6.70%, *N* = 4 spinal cords, *n* = 5/4/5/5 sections from each spinal cord; lower lumber: Vglut2^+ ^= 83.54 ± 6.29% and Vglut2^+^/VIAAT^+ ^= 14.89 ± 6.89%, *N* = 4, *n* = 5/5/5/5] (Fig. 4*C,D*). None of these neurons express only VIAAT (Fig. 4*C,D*). These data confirm that the Lhx9-derived neurons are all excitatory.

**Figure 5.**
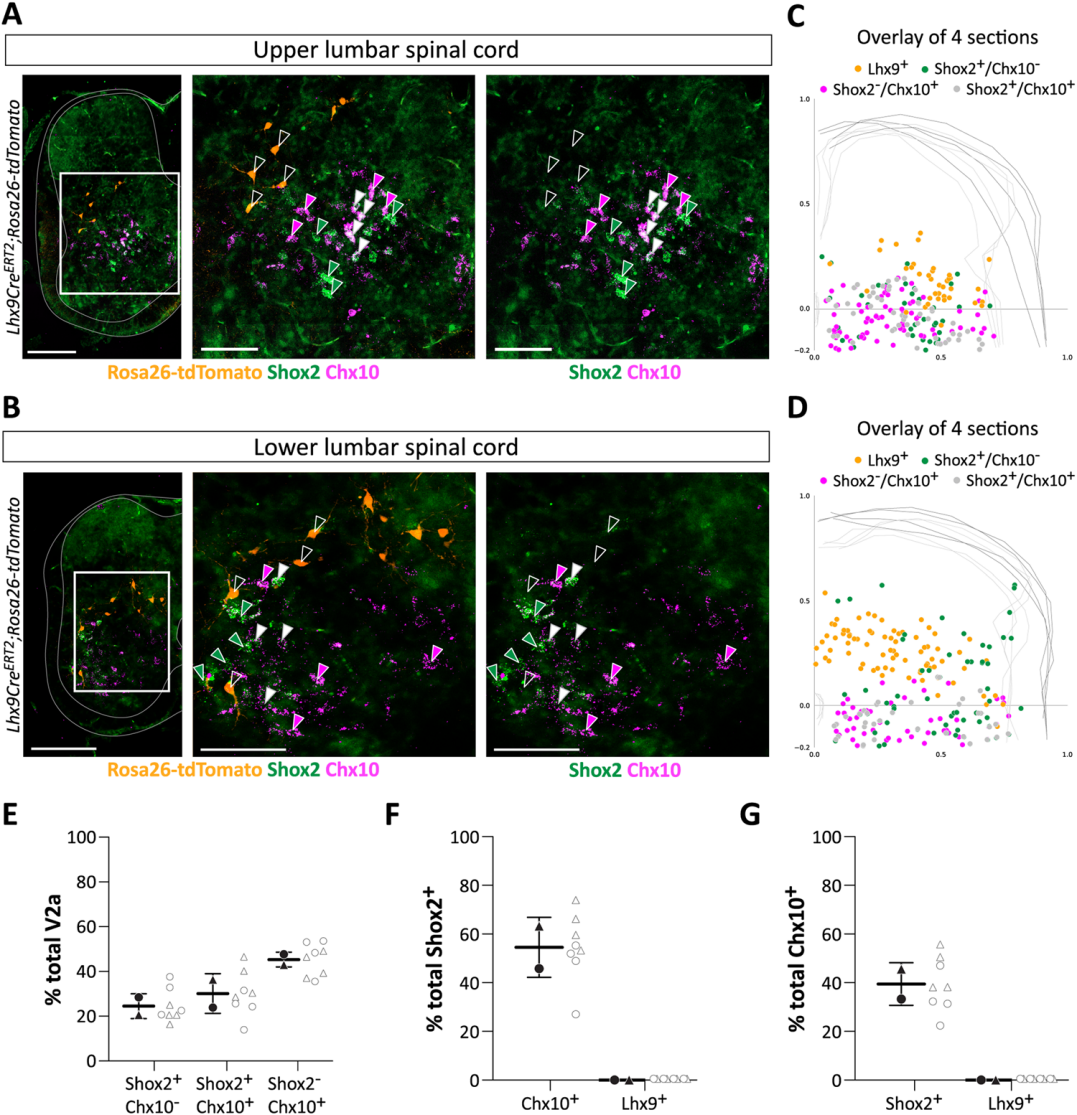
Lhx9-derived neurons define a molecularly distinct population in the lumbar cord. ***A,B***, Images of a hemi-section from the upper lumbar (***A***) and lower lumbar (***B***) spinal cord showing Shox2 (*Shox2*, in green), Chx10 (*Vsx2*, in magenta) and Lhx9-derived neurons (in orange) visualized by combination RNAscope in situ hybridization (Shox2 and Chx10) and immunohistochemistry (Lhx9) in *Lhx9Cre^ERT2^;Rosa26-tdTomato* neonatal mice (E18.5). Rightmost pictures are enlargement of the white boxed areas. Scale bar for the hemi-sections, 200 µm; magnified boxed, 100 µm. Empty white arrows point to the location of four Lhx9-derived neurons, filled green arrows point to the location of four Shox2^+^/Chx10^−^ cells, filled magenta arrows point to the location of four Shox2^−^/Chx10^+^ cells and filled white arrows point to the location of four Shox2^+^/Chx10^+^ cells. ***C,D***, Spatial distribution and visualization (overlay of 4 sections) of Lhx9-derived neurons (orange), Shox2^+^/Chx10^−^ neurons (green), Shox2^−^/Chx10^+^ neurons (magenta) and Shox2^+^/Chx10^+^ neurons (gray) in the upper (***C***) and lower (***D***) lumbar spinal cord. One dot represents one cell. ***E***, Percentage wise distribution of Shox2^+^/Chx10^−^, Shox2^−^/Chx10^+^ or Shox2^+^/Chx10^+^. ***F***, Percent of Chx10^+^ cells or Lhx9-derived neurons that co-express Shox2^+^. ***G***, Percent of Shox2^+^ cells or Lhx9-derived neurons that co-express Chx10^+^. For all graphs, mean ± SD. Each marker (black) represents the mean % of neurons per spinal cord with data from individual sections to the left (gray open dots).

To determine the relationship of Lhx9-derived neurons to other groups of excitatory spinal populations involved in the locomotor network, we used probes labeling Shox2 neurons (*Shox2* probe) or Chx10 neurons (*Vsx2* probe) (Fig. 5). Previous studies have shown that Shox2 and Chx10 markers delineate three populations of neurons in the spinal cord: Chx10 only (Shox2^−^/Chx10^+^), Shox2^+^ and Chx10^+^, and Shox2^+^ only (Shox2^+^/Chx10^−^) neurons with essential roles in the locomotor network (Crone et al., 2008, 2009; Dougherty et al., 2013; Ha and Dougherty, 2018). We find that of all neurons with these two makers, 45.33 ± 3.32% (mean ± SD) were Chx10 only (Shox2^−^/Chx10^+^), 30.14 ± 8.87% (mean ± SD) were both Shox2^+^/Chx10^+^, and 24.53 ± 5.54% (mean ± SD) were Shox2^+^ only (Shox2^+^/Chx10^−^) (Fig. 5*E*; *N* = 2, *n* = 4/4). We also found that 54.55 ± 12.36% (mean ± SD) of Shox2^+^ neurons co-expressed the Chx10 transcript and 39.46 ± 8.72% (mean ± SD) of Chx10^+^ neurons expressed the Shox2 transcript (Fig. 5*F,G*) which corroborates with previous studies (Dougherty et al., 2013). More importantly, we did not find any overlap between Lhx9-derived neurons and Chx10^+^ neurons or Shox2^+^ neurons (Fig. 5*F,G*) even though their spatial distributions are in proximity to each other (Fig. 5*A–D*). The combined set of data shows that Lhx9-derived neurons define an excitatory population in the spinal locomotor region distinct from these previously characterized excitatory populations.

**Figure 6.**
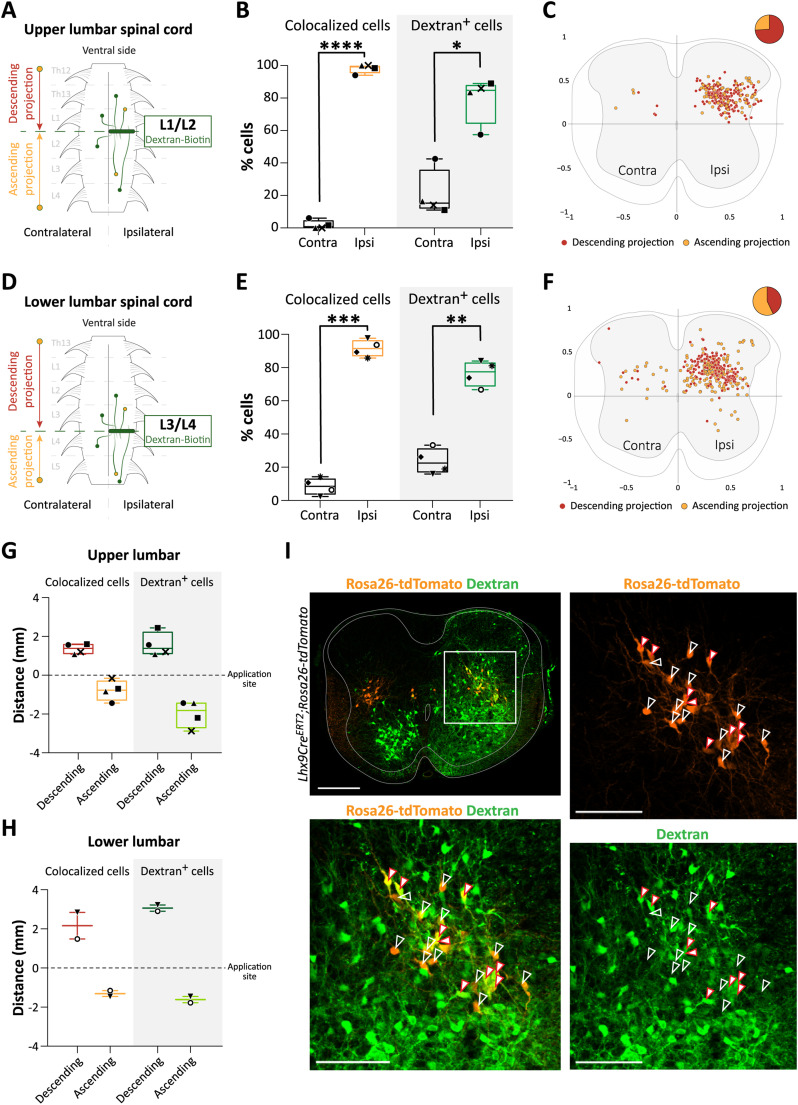
Lhx9-derived neurons are ipsilaterally projecting with a mix of ascending and descending projections. ***A***, Schematic of *Lhx9Cre^ERT2^;Rosa26-tdTomato* spinal cord (E18.5) showing the unilateral application of the retrograde marker (Dextran-Biotin, 3,000 MW) in the upper lumbar spinal cord (L1/L2). The schematic indicates Lhx9-derived neurons (yellow) and non-Lhx9-derived cells (green) that are retrogradely labeled rostral (descending projection) and caudal (ascending projection) to the application site. ***B***, Quantification (in percent) of cells that colocalize Lhx9 and Dextran (*left panel*) and Dextran^+^ cells that are Lhx9^−^ (*right panel*), ipsilateral (ipsi) and contralateral (contra) to the application site (****p < 0.0001 (*t *= 33.93, df = 3), *p = 0.028 (*t *= 3.996, df = 3), two-tailed, paired *t* test for all). ***C***, Spatial distribution of descending and ascending projecting Lhx9-derived neurons labeled from the upper lumbar spinal cord. One dot represents a cell that colocalized Lhx9 and Dextran, descending projecting neurons (dark orange) or ascending projecting neurons (light orange). Inset shows the proportion in percentage of descending (73.95%, dark orange) versus ascending (26.05%, light orange) projecting neurons. ***D***, Schematic of *Lhx9Cre^ERT2^;Rosa26-tdTomato* spinal cord (E18.5) showing the unilateral application of the retrograde marker in the lower lumbar spinal cord (L3/L4). ***E***, Quantification (in percent) of cells that colocalize Lhx9^+^ and Dextran (*left panel*) and Dextran^+^ cells that are Lhx9^−^ (*right panel*), ipsilaterally (ipsi) and contralaterally (contra) to the application site (***p = 0.0005 (*t *= 16.10, df = 3), **p = 0.0065 (*t *= 6.807, df = 3), two-tailed, paired *t* test for all). ***F***, Spatial distribution of descending (dark orange) and ascending (light orange) projecting Lhx9-derived neurons labeled from the lower lumbar spinal cord. Inset shows the proportion in percentage of descending (42.98%, dark orange) versus ascending (57.02%, light orange) projecting neurons. ***G,H***, Quantification of the maximum distance (in mm) from the application site where colocalized cells can be found for the descending and ascending projections in the upper lumbar (***G***) and in the lower lumbar (***H***) spinal cord. ***I***, Image of an entire transverse spinal section (E18.5) after Dextran-biotin application (top left picture) and enlargement of the white boxed area (bottom left and rightmost pictures). Lhx9-derived neurons in orange and back-labeled non-Lhx9Cre cells in green. Filled white arrows with red line point to colabeled cells (Lhx9^+^/Dextran^+^, expressing the color orange in the merged picture (bottom left)). Empty white arrows point to Lhx9-derived neurons that are not colabeled with Dextran (that have ascending axons or axons ending rostral to the dye application). Scale, 200 µm for the entire section (top left), 100 µm for the magnified boxed area (bottom left and rightmost pictures). Graphs show min-to-max box plot with medians. Each marker (black) represents either the mean of neurons per spinal cord or the maximum distance from the application site per spinal cord. The same marker represents the same spinal cord between graphs.

To determine the projection pattern of Lhx9-derived neurons, we performed a unilateral application of a retrograde marker (Dextran-Biotin, 3,000 MW) either in the upper lumbar (level 1-2, L1/L2) or in lower lumbar (level 3–4, L3/L4) spinal cord (E18.5; Fig. 6*A,D,I*). We found that, in both conditions, Lhx9-derived neurons are almost exclusively ipsilaterally projecting [(mean ± SD for all), upper lumbar spinal cord: ipsilateral = 98.10 ± 2.83% vs contralateral = 1.90 ± 2.83%, *N* = 4 spinal cords, *n* = 28/26/79/73 sections per animal; lower lumbar: ipsilateral = 91.56 ± 5.16% vs contralateral = 8.44 ± 5.16%, *N* = 4, *n* = 17/16/102/92] (Fig. 6*B,E*). Lhx9-derived neurons labeled from L1/L2 were mainly descending (73.95%) and found rostral to the application site while a smaller fraction of them were ascending from lumbar levels located more caudally (26.05%) (Fig. 6*C*). Lhx9-derived neurons labeled from L3/L4 were almost equally distributed between descending projections (42.98%) from more rostral levels and ascending projections (57.02%) from more caudal levels (Fig. 6*F*). The revealed projections are mainly local within the lumbar cord as colocalized (Lhx9^+^ Dextran^+^) neurons were found within 2–3 segments from the application site. However, in the lower lumbar, Lhx9-derived neurons had longer projections than in the upper lumbar cord [mean distance (in mm) from the cut (mean ± SD for all): upper lumbar, descending = 1.36 ± 0.26 vs ascending = 0.79 ± 0.53, *N* = 4 spinal cords; lower lumbar, descending = 2.16 ± 0.26 vs ascending = 1.31 ± 0.21, *N* = 2] (Fig. 6*G,H*). This was not due to inefficiency of the labeling since Dextran^+^/Lhx9^−^ cells were found further away both rostral and caudal to the application site (Fig. 6*G,H*). In addition, Dextran^+^/Lhx9^−^ cells were found contralateral to the application sites (upper lumbar: *N* = 4 spinal cords, *n* = 28/26/79/73 sections per spinal cord; lower lumbar *N* = 4, *n* = 17/16/102/92) (Fig. 6*B,E,I*).

Altogether these data show that Lhx9-derived neurons are distributed along the lumbar spinal cord, are mainly glutamatergic and ipsilaterally projecting along the cord. They also depict a neuronal population which does not overlap with several of the main populations involved in the locomotor network, the V2a and the Shox2^+^/Chx10^−^ neurons. Lhx9-derived neurons in the locomotor region possess all the hallmark of neurons that might involve the excitatory components of the spinal locomotor network, including the rhythm generation circuitries. Therefore, in the next step, we investigated the functional role of this population by using electrophysiology experiments.

### Chronic silencing the synaptic signaling of excitatory Lhx9-derived neurons reduces the frequency of locomotor-like activity

As a first step to determine the functional role of Lhx9-derived neurons, we performed locomotor experiments in spinal cords isolated from early postnatal mice (P0–P3) after chronically ablating the excitatory synaptic transmission from the targeted cells. For this we used a genetic approach that selectively targets the glutamatergic transmission of the Lhx9-derived neurons. By crossing the *Lhx9Cre^ERT2^* mice with mice carrying a conditional floxed *Vglut2* allele *(Vglut2^Flx/Flx^*) we aimed at creating a Cre-dependent loss of Vglut2 in Lhx9-derived neurons. This approach has been shown in previous work from our lab and others to efficiently block action potential mediated synaptic transmission from the affected neurons (Talpalar et al., 2011; Bui et al., 2013; Dougherty et al., 2013; Caldeira et al., 2017). In correspondence with the level of recombination we found in the *Lhx9Cre^ERT2^;Rosa26-tdTomato* neonatal mice, the cross of *Lhx9Cre^ERT2^;Vglut2^FlxFlx^* led to deletion of Vglut2 transcripts (*Slc17a6* probe) in 62.30 ± 3% (mean ± SD) of the recombined cells while it was expressed in 37.70 ± 3% (mean ± SD) (*N* = 3 spinal cords, *n* = 5/4/4 sections per spinal cord).

**Figure 7.**
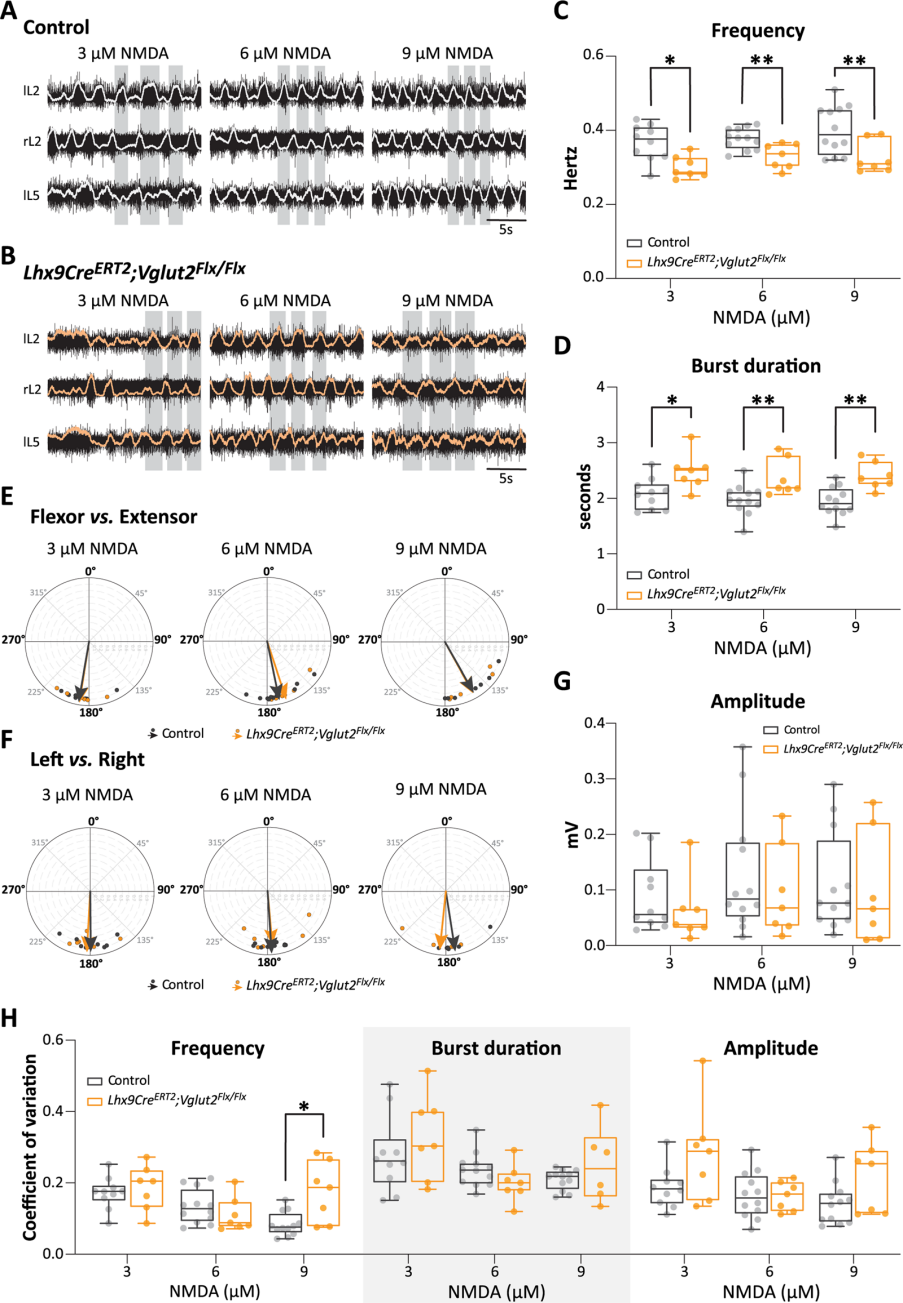
Chronic silencing of Lhx9-derived neurons reduces the frequency of the locomotor-like activity in the lumbar spinal cord. ***A,B***, Example of locomotor-like activity in littermate controls (***A***) and *Lhx9Cre^ERT2^;Vglut2^Flx/Flx^* (***B***) mice (raw ventral root recordings in black, rectified in white and orange, respectively) in 8 µM 5-HT and different NMDA concentrations (3, 6, and 9 µM). All recordings were done between P0 and P3. r, right and l, left. ***C***, Quantification of the frequency at each NMDA concentration (3 µM, *p = 0.0105, 6 µM, **p = 0.0077, 9 µM, **p = 0.0091, two-tailed, Mann–Whitney's test for all). ***D***, Quantification of the burst duration at each NMDA concentration (3 µM, *p = 0.025, 6 µM, **p = 0.0098, 9 µM, **p = 0.0018, two-tailed, Mann–Whitney's test for all). ***E,F***, Circular plots representing the phase relationship between ipsilateral flexor–extensor (L2 vs L5) in (***E***) and left–right side ventral roots in (***F***) of littermate controls and *Lhx9Cre^ERT2^;Vglut2^Flx/Flx^* mice. Each dot represents the mean angle for individual spinal cord, arrow represents the mean vector for each group. The mean angle is not significantly different between littermate controls and *Lhx9Cre^ERT2^;Vglut2^Flx/Flx^* mice for all measurements (flexor–extensor (***E***), 3 µM, *p* = 0.94, 6 µM, *p* = 0.52 and 9 µM, *p* = 0.96, Watson-William's test. Left–right (***F***), 3 µM, *p* = 0.73, 6 µM, *p* = 0.91 and 9 µM, *p* = 0.28, Watson-William's test) ***G***, Quantification of the amplitude at each NMDA concentration (3 µM, *p* = 0.3148, 6 µM, *p* = 0.6504, 9 µM, *p* = 0.5358, two-tailed, Mann–Whitney's for all) ***H***, Coefficient of variation for the main locomotor parameters (*p = 0.029, two-tailed, unpaired *t* test with Welch's correction, *t *= 2.73, df = 7.12). For all graphs, min-to-max box plot with medians. Controls in gray/dark gray and *Lhx9Cre^ERT2^;Vglut2^Flx/Flx^* in orange/dark orange. Each dot represents an individual spinal cord.

We induced locomotor-like activity in the isolated lumbar spinal cord (P0–P3) by bath application of n-methyl-D-aspartate (NMDA; 3, 6 or 9 µM) in combination with 5-hydroxytryptamine (5-HT; 8 µM). We used different concentrations of NMDA to cover a range of locomotor frequencies (Talpalar and Kiehn, 2010). The locomotor-like activities were recorded from the lumbar (L) flexor (L2) and extensor (L5) related roots on both sides of the cord. We were still able to induce locomotor activity in *Lhx9Cre^ERT2^;Vglut2^Flx/Flx^* spinal cords (*N* = 7 spinal cords) (Fig. 7*A,B*). However, the locomotor frequencies in these preparations were 18.2%, 12.4% and 18.5% lower than of controls (*N* = 10, 12 and 12) at all NMDA concentrations (3, 6 and 9 µM) (Fig. 7*C*). These changes were significant for all drug concentrations (3 µM: *p = 0.0105, 6 µM: **p = 0.0077 and 9 µM: **p = 0.0091, two-tailed, Mann–Whitney's test for all). The reduction in frequencies in *Lhx9Cre^ERT2^;Vglut2^Flx/Flx^* mice compared to littermate controls was accompanied by a significant increase in burst duration (Fig. 7*D*) (3 µM: *p = 0.025, 6 µM: **p = 0.0098, and 9 µM: **p = 0.0018, two-tailed, Mann–Whitney's test for all) between these two groups. However, there were no changes in either the burst amplitude or the duty cycle (Fig. 7*G,H*). We also observed an increased variability of the frequency at higher drug concentration (9 µM: *p = 0.029, two-tailed, unpaired *t* test with Welch's correction, *t *= 2.73, df = 7.12) (Fig. 7*H*). Notably, the chronic deletion did not affect the coordination between ipsilateral flexor–extensor (L2 vs L5) and corresponding left–right ventral root activity (left L2 and right L2 or left L5 and right L5). They remained alternation with phase values around 180° both in *Lhx9Cre^ERT2^;Vglut2^Flx/Flx^* mice and littermate controls for all drug concentrations (3, 6 and 9 µM NMDA, respectively, for flexor–extensor, controls: *N* = 8/11/7 and *Lhx9Cre^ERT2^;Vglut2^Flx/Flx^*: *N* = 7/7/5; for left–right, controls: *N* = 9/12/7 and *Lhx9Cre^ERT2^;Vglut2^Flx/Flx^*: *N* = 6/6/5) (Fig. 7*E,F*) with no difference between the littermate controls and *Lhx9Cre^ERT2^;Vglut2^Flx/Flx^* spinal cords (for flexor–extensor: 3 µM: *p *= 0.94, 6 µM: *p *= 0.52 and 9 µM: *p *= 0.96, Watson-William's test. For the left–right: 3 µM: *p *= 0.73, 6 µM: *p *= 0.91 and 9 µM: *p *= 0.28, Watson-William's test).

These findings indicate that chronically eliminating glutamatergic output from Lhx9-derived neurons leads to a decrease in the frequency of locomotor-like activity. Since the Vglut2 removal affected just over 60% of these neurons, it's plausible that completely removing them might reveal a more pronounced effect.

### Acute silencing of Lhx9-derived neurons decreases frequency of locomotor-like activity

**Figure 8.**
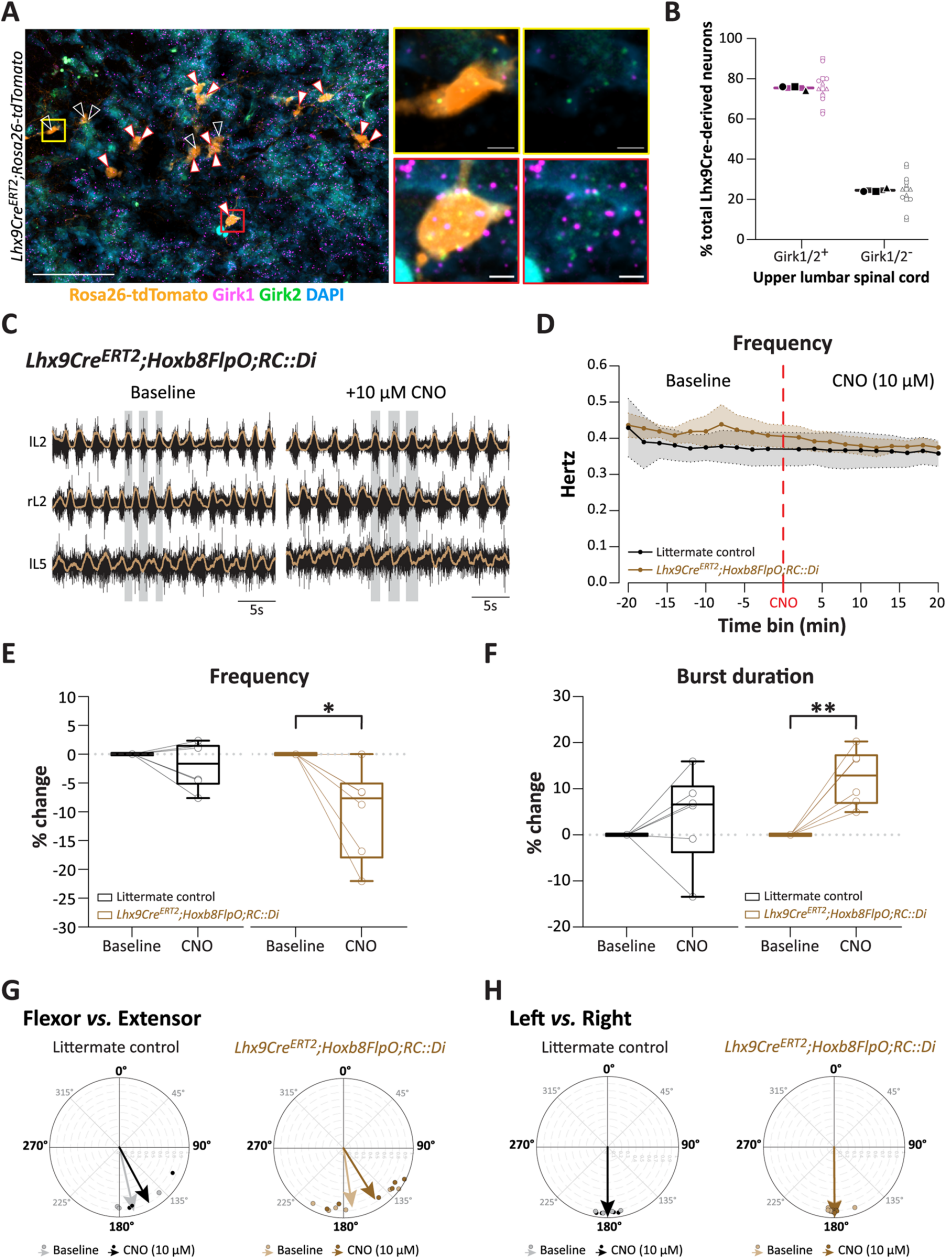
Acute silencing of Lhx9-derived neurons decreases the frequency of locomotor-like activity. ***A***, Images from the upper lumbar spinal cord showing Girk1 (in magenta), Girk2 (in green) and Lhx9-derived neurons (in orange) visualized by combining RNAscope in situ hybridization (Girk1 and Girk2) and immunohistochemistry (Lhx9) in *Lhx9Cre^ERT2^;Rosa26-tdTomato* neonatal mice (E18.5). Rightmost pictures are enlargement of the yellow (cell negative for Girk1/2) and red (cell positive for Girk1/2) boxed areas. Empty white arrows point to Lhx9-derived neurons that are negative for either Girk1 and Girk2. Filled white arrows with red line point to Lhx9-derived neurons that are positive for either Girk1 and Girk2. Scale bar for, 100 µm; magnified boxes, 5 µm. ***B***, Percent of total Lhx9-derived neurons expressing either Girk1/2 (in magenta) or none of the markers (in black) (*N* = 3 spinal cords, *n* = 5 sections/spinal cord). Graph representing mean ± SD. Each marker (black) represents the mean % of neurons per spinal cord with data from individual section to the left (open dots in magenta or gray). ***C***, Ventral root recordings on the left (l) and right (r) side at L2 and L5 in *Lhx9Cre^ERT2^;Hoxb8FlpO;RC::Di* neonatal mice before (baseline) and during incubation with CNO (10 µM). ***D***, Locomotor frequency (Hz) plotted over time (min) for littermate control (black, *N* = 6) and *Lhx9Cre^ERT2^;Hoxb8FlpO;RC::Di* (brown, *N* = 6) neonatal mice. Graph representing mean (bold line) ± SD (shaded area) with a 2 min bin. ***E***, Quantification of the frequency for littermate control (*N* = 6; left panel, black) and *Lhx9Cre^ERT2^;Hoxb8FlpO;RC::Di* mice (*N* = 6; right panel, dark brown) as a % change compare to baseline (littermate control, *p *= 0.2951 (*t *= 1.169, df = 5) and *Lhx9Cre^ERT2^;Hoxb8FlpO;RC::Di* mice *p = 0.0264 (*t *= 3.116, df = 5), two-tailed, paired *t* test for all). ***F***, Quantification of the burst duration for littermate control (*N* = 6; left panel, black) and *Lhx9Cre^ERT2^;Hoxb8FlpO;RC::Di* mice (*N* = 6; right panel, brown) as a % change compare to baseline (littermate control, *p *= 0.3783 (*t *= 0.9663, df = 5) and *Lhx9Cre^ERT2^;Hoxb8FlpO;RC::Di *mice, **p = 0.0042 (*t *= 4.985, df = 5), two-tailed, paired *t* test for all). For ***E,F***, Min-to-max box plot withe medians. Littermate controls in gray/dark gray and *Lhx9Cre^ERT2^;Hoxb8FlpO;RC::Di* in light brown/dark brown. Each dot represents an individual spinal cord. ***G,H***, Circular plots representing the phase relationship between ipsilateral flexor–extensor (L2 vs L5) in (***G***) and left–right side ventral roots in (***H***) of littermate controls (baseline in light gray, CNO condition in black) and *Lhx9Cre^ERT2^;Hoxb8FlpO;RC::Di* mice (baseline in light brown, CNO condition in dark brown). Each dot represents the mean angle for individual spinal cord while the arrow represents the mean vector for each condition (baseline vs CNO). The mean angle is not significantly different between baseline and CNO condition for the littermate controls and *Lhx9Cre^ERT2^;Hoxb8FlpO;RC::Di* mice (flexor–extensor (***G***), littermate control (*N* = 3), *p *= 0.5373 and *Lhx9Cre^ERT2^;Hoxb8FlpO;RC::Di* mice (*N* = 6), *p *= 0.2607, Watson-William's test. Left–right (***H***), littermate control (*N* = 4), *p *= 0.9676 and *Lhx9Cre^ERT2^;Hoxb8FlpO;RC::Di* mice (*N* = 5), *p *= 0.9643, Watson-William's test).

To further substantiate the frequency modulating role of Lhx9-derived neurons, we performed acute silencing experiments using inhibitory DREADDs (Roth, 2016). For this, we used the double conditional *RC::FPDi* mouse line that allows targeting expression of CNO-activated Gi-coupled human M4 muscarinic receptors (hM4Di) after Cre and flp recombinase. We performed a triple cross where we first crossed *Hoxb8FlpO* mice with *RC::FPDi* mice. Hoxb8 is expressed from the cervical to the tip of the spinal cord (Talpalar et al., 2013; Allodi et al., 2021) allowing us to specifically target the cord. We then crossed *Hoxb8FlpO;RC::PDi* mice with *Lhx9Cre^ERT2^* mice to obtain *Lhx9Cre^ERT2^;Hoxb8FlpO;RC::Di* neonatal mice for experiments (P0–P3). This should lead to a specific expression of hM4Di receptor in Lhx9-derived neurons in the spinal cord. hM4Di is activated by clozapine-N-oxide (CNO) and obtains its action through a G protein-gated inwardly rectifying potassium channels (GIRKs). Therefore, CNO action is dependent on the intrinsic expression of GIRK channels in Lhx9-derived neurons. To verify the expression of GIRK channels (Girk1 and Girk2), we performed RNAscope in situ hybridization with Girk1/2 probes followed by immunohistochemistry against tdTomato (Fig. 8*A*). We found that 70% of Lhx9-derived neurons express either Girk1 or Girk2 while 30% of the neurons do not express neither Girk1 nor Girk2 (*N* = 3 spinal cords, *n* = 5 sections/spinal cord) (Fig. 8*B*). CNO is therefore expected to only affect a subpopulation of Lhx9-derived neurons.

After reaching a stable baseline locomotor activity, CNO (10 µM) was added to the Ringer containing the locomotor drug solution (6.5 µM NMDA and 8 µM 5-HT) (Fig. 8*C,D*). In *Lhx9Cre^ERT2^;Hoxb8FlpO;RC::Di* mice (P0-P3; *N* = 6), CNO application caused a significant decrease of the locomotor frequency compare to baseline which developed over 15–25 min (average decrease 10.12% with a maximum of 22%; *p = 0.0264, two-tailed, paired *t* test, *t *= 3.116, df = 5) (Fig. 8*E*, right panel). The frequency change was accompanied by a significantly increase (12.5% on average) in the ventral root burst duration (**p = 0.0042, two-tailed, paired *t* test, *t *= 4.985, df = 5) (Fig. 8*F*, right panel), while the reduction of the frequency did not affect the alternation between ipsilateral flexor–extensor (L2 vs L5; *N* = 6) or the left–right ventral root activity (left L2 and right L2; *N* = 5) (Fig. 8*G,H*, right panel for both). In littermate controls (*WT;RC::FPDi*; *N* = 6), CNO caused a slight but insignificant decrease in the frequency (average reduction of 1.9%) (*p *= 0.2951, two-tailed, paired *t* test, *t *= 1.169, df = 5) (Fig. 8*E*, left panel), variable change in burst duration (on average a 4% increase; *p *= 0.3783, two-tailed, paired *t* test, *t *= 0.9663, df = 5) (Fig. 8*F*, left panel), and no change in flexor–extensor and left–right coordination (Fig. 8*G,H*, left panel for both).

Altogether, the data on chronic and acute silencing of Lhx9-derived neurons suggest that they play a role in the spinal locomotor network by modulating the frequency of locomotor-like activity.

### Optogenetic activation of Lhx9-derived neurons modulates the frequency of locomotor-like activity

**Figure 9.**
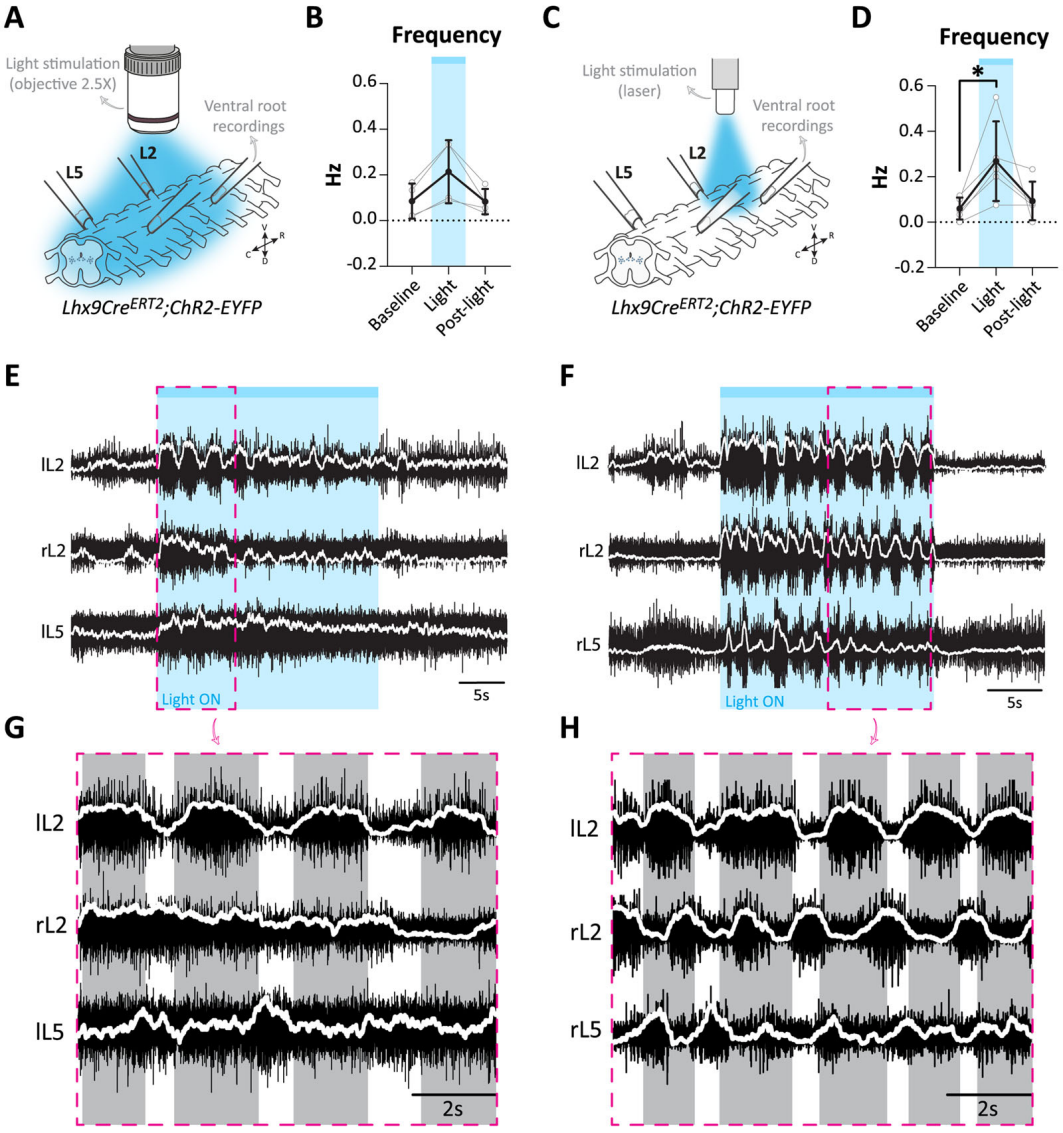
Optogenetic activation of Lhx9-derived neurons can initiate locomotor bursts. ***A***, Schematic of experimental setup. Light was delivered with a 2.5× objective to target the full length of the lumbar spinal cord (L1–L6). Ventral root recordings on the left (l) and right (r) side at L2 and L5 in *Lhx9Cre^ERT2^;ChR2-EYFP* neonatal mice (P0–P3). ***B***, Quantification of the locomotor-like frequency during baseline, during light stimulation and the post-light period (*p *= 0.1250 baseline vs light (*W* = 10), *p *> 0.9999 baseline vs post-light (*W* = 0), two-tailed, Wilcoxon matched-pairs signed rank test) (*N* = 4 spinal cords, *n* = 9 trials). ***C***, Schematic of experimental setup with light stimulation of the upper lumbar spinal cord (L1/L2) in *Lhx9Cre^ERT2^;ChR2-EYFP* neonatal mice (P0–P3). ***D***, Quantification of locomotor-like frequency during baseline, light and post-light (*p = 0.0309 baseline vs light (*t *= 3.266, df = 4), *p* = 0.4141 baseline vs post-light (*t *= 0.9105, df = 4), two-tailed, paired *t* test) (*N* = 5, *n* = 13). ***E–H***, Example of ventral roots recording during the full lumbar stimulation (***E***) and the upper lumbar stimulation (***F***) with magnified areas (pink dash line) in (***G,H***) respectively (raw recording in black and rectified recording in white). D, dorsal; V, ventral; R, rostral; C, caudal. For all graphs, gray lines and dots represent the mean for each spinal cord (mean of 2–3 trials); black lines and dots represent the overall mean ± SD. For all ventral roots recording, blue shaded area is indicating when the light is ON. All recordings were done between P0 and P3.

To evaluate if Lhx9-derived neurons contribute to activation of locomotor-like activity or to frequency modulation of locomotion, we crossed *ChR2-EYFP* mice with *Lhx9Cre^ERT2^* mice to obtain expression of channelrhodopsin-2 (ChR2) in Lhx9^+^ neurons. For light-activation of Lhx9-derived neurons, we used continuous broad light stimulation targeting the entire lumbar region from L1 to L6 (duration: 30 s) (Fig. 9*A*) or local light stimulation targeting 1–2 segment(s) either L1/L2 or L5/L6 (duration: 20 s) (Fig. 9*C*).

On a background of low doses of 5-HT (3 or 8 µM) — which does not itself induce locomotor-like activity but increases the spinal neuron activity — stimulation of L1–L6 segments induced locomotor bouts lasting for the time of stimulation. The frequency of bursting in the locomotor bouts was clearly above baseline values [frequency (mean ± SD for all), baseline = 0.085 ± 0.077 Hz vs light = 0.21 ± 0.14 Hz; *N* = 4 spinal cords, *n* = 9 trials in total] (Fig. 9*B*).

Since there is a known rostro-caudal gradient in rhythmogenic potential in the lumbar spinal cord (Cazalets et al., 1995; Kjaerulff and Kiehn, 1996; Cowley and Schmidt, 1997; Kremer and Lev-Toy, 1997), we next examined the effect of light stimulation applied locally to the upper lumbar spinal cord (L1–L2). This stimulation reliably induced locomotor-like activity from baseline condition with a much stronger frequency modulation than broad L1–L6 stimulation [mean light = 0.27 ± 0.18 Hz (mean ± SD); *N* = 5 spinal cords, *n* = 13 trials in total] (Fig. 9*D*). At light-onset, both broad (L1–L6) and local (L1–L2) stimulation elicited locomotor-like activity almost instantaneously which terminated abruptly at light-offset (post-light) (Fig. 9*E–H*). The locomotor-like activity induced by light activation of Lhx9-derived neurons resulted in a pattern activity with ipsilateral flexor–extensor (L2 vs L5) and left–right alternation (rL2 vs lL2) (Fig. 9*E–H*).

These experiments show that activation of Lhx9-derived neurons can initiate locomotor-like activity from a non-rhythmic state. We next set out to evaluate if the stimulation also could modulate on-going activity.

**Figure 10.**
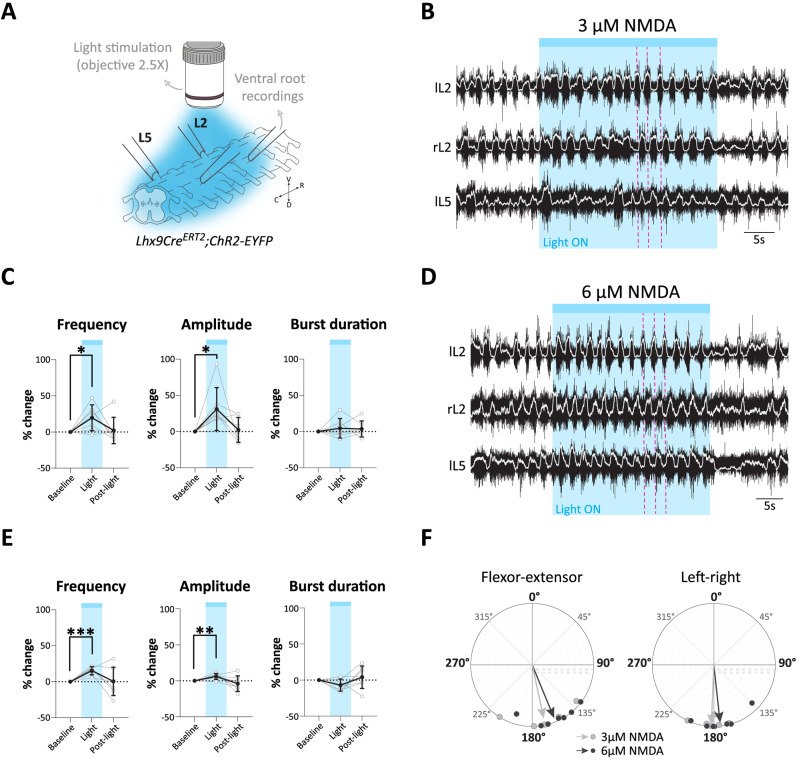
Broad activation of Lhx9-derived neurons during on-going locomotor-like activity. ***A***, Schematic of the experimental setup for broad stimulation. ***B***, Example of rhythmic activity before, during and after L1–L6 lumbar stimulation (30 s) - 3 µM NMDA and 8 µM 5-HT. ***C***, Quantification of the frequency (*left panel*), burst amplitude (*middle panel*) and burst duration (*right panel*) of locomotor-like activity as % change from the baseline (*Left panel*, frequency, *p = 0.0469 baseline vs light (*W* = 24.00), *p *= 0.5 baseline vs post-light (*W* = −7). *Middle panel*, amplitude, *p = 0.0156 baseline vs light (*W* = 28), *p *= 0.9375 baseline vs post-light (*W* = 2). For both graphs, two-tailed, Wilcoxon's matched-pairs signed rank test. *Right panel*, burst duration, *p *= 0.4140 baseline vs light (*t *= 0.8773, df = 6), *p *= 0.4223 baseline vs post-light (*t *= 0.8611, df = 6), two-tailed, paired *t* test) (*N* = 7 spinal cords, *n* = 18 trials in total). ***D***, Example of rhythmic activity before, during and after broad lumbar stimulation (30 s) - 6 µM NMDA and 8 µM 5-HT. ***E***, Quantification of frequency (*left panel*), burst amplitude (*middle panel*), and burst duration (*right panel*) of locomotor-like activity (broad lumbar stimulation; 6 µM NMDA and 8 µM 5-HT) (*Left panel*, frequency, ***p = 0.0004 baseline vs light (*t *=7.106, df = 6) and *p *= 0.9481 baseline vs post-light (*t *= 0.06781, df = 6). *Middle panel*, amplitude, **p = 0.0071 baseline vs light (*t *= 4.006, df = 6) and *p *= 0.3726 baseline vs post-light (*t *= 0.9632, df = 6). *Right panel*, burst duration, *p* = 0.0710 baseline vs light (*t *= 2.191, df = 6), *p* = 0.5234 baseline vs post-light (*t* = 0.6775, df = 6). For all graphs, two-tailed, paired *t* test) (*N* = 7, *n* = 15). ***F***, Circular plots representing the phase relationship between ipsilateral flexor–extensor (FE) (L2 vs L5) (*left panel*) and left–right (LR) side of the cord (*right panel*) during L1–L6 lumbar stimulation in 3 µM NMDA (light gray; FE, *N* = 5, *n* = 13; LR, *N* = 7, *n* = 18) and 6 µM NMDA (dark gray; FE, *N* = 7, *n* = 15; LR, *N* = 7, *n* = 15). For all graphs, gray lines and dots represent the mean for each spinal cord (mean of 2–3 trials); black lines and dots represent the overall mean ± SD. For all ventral roots recording, blue shaded area is indicating when the light is ON. All recordings were done between P0 and P3. D, dorsal; V, ventral; R, rostral; C, caudal.

For this we used either broad (L1–L6) or local (L1/L2 or L5/L6) stimulation of the Lhx9-derived neurons. At relatively low locomotor-like frequencies (3 µM NMDA; *N* = 7 spinal cords, *n* = 18 trials in total), broad stimulation of Lhx9-derived neurons (Fig. 10*A,B*) led to a visible and statistically significant increase of the frequency (*p = 0.0469 baseline vs light, two-tailed, Wilcoxon matched-pairs signed rank test, *W* = 24) and the burst amplitude (*p = 0.0156, two-tailed, Wilcoxon matched-pairs signed rank test, *W* = 28) (Fig. 10*C*). There were no changes in the burst duration (Fig. 10*C*, right panel). At higher locomotor-like frequencies (6 µM of NMDA; *N* = 7, *n* = 15), broad stimulation also leads to a significant increase of the frequency and the burst amplitude (respectively, ***p = 0.0004 (*t *= 7.106, df = 6) and *p = 0.0071 (*t *= 4.006, df = 6), for baseline vs light, two-tailed, paired *t* test) (Fig. 10*D,E*) with a slight reduction of the burst duration (Fig. 10*E*, right panel).

**Figure 11.**
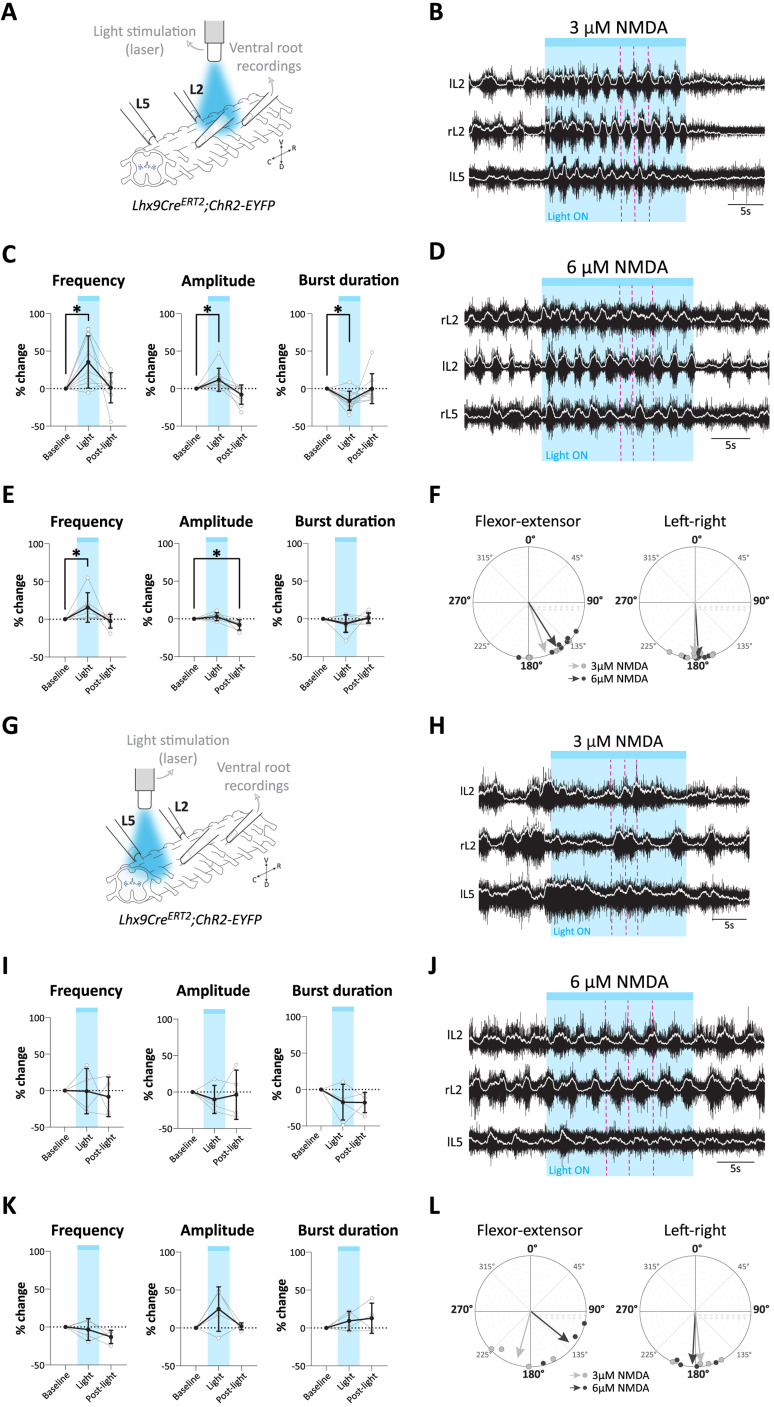
Local activation of Lhx9-derived neurons during on-going locomotor-like activity. ***A***, Schematic of the experimental setup showing local light stimulation (L1/L2). ***B***, Example of rhythmic activity before, during and after local stimulation (L1/L2; 20 s) in 3 µM NMDA and 8 µM 5-HT. ***C***, Quantification of frequency of locomotor-like activity (*left panel*), burst amplitude (*middle panel*), and burst duration (*right panel*) (local stimulation (L1/L2); 3 µM NMDA and 8 µM 5-HT) (*Left panel*, frequency, *p = 0.0159 baseline vs light (*t *= 3.046, df = 8), *p *= 0.8713 baseline vs post-light (*t *= 0.1673, df = 8), two-tailed, paired *t* test. *Middle panel*, amplitude, *p = 0.0195 baseline vs light (*W* = 39), *p *= 0.4258 baseline vs post-light (*W* = −15). *Right panel*, burst duration, *p = 0.0117 baseline vs light (*W* = −41), *p* = 0.3594 baseline vs post-light (*W* = −17). For both graphs, two-tailed, Wilcoxon's matched-pairs signed rank test) (*N* = 9, *n* = 25). ***D***, Example of rhythmic activity before, during and after local stimulation (L1/L2; 20 s) in 6 µM NMDA and 8 µM 5-HT. ***E***, Quantification of frequency (*left panel*), burst amplitude (*middle panel*), and burst duration (*right panel*) of locomotor-like activity (local stimulation (L1/L2); 6 µM NMDA and 8 µM 5-HT) (*Left panel*, frequency, *p = 0.0156 baseline vs light (*W* = 28), *p *> 0.9999 baseline vs post-light (*W* = −1), two-tailed, Wilcoxon's matched-pairs signed rank test. *Middle panel*, amplitude, *p *= 0.1941 baseline vs light (*t *= 1.462, df = 6), *p = 0.0210 baseline vs post-light (*t *= 3.103, df = 6). *Right panel*, burst duration, *p* = 0.2039 baseline vs light (*t *= 1.425, df = 6), *p* = 0.6664 baseline vs post-light (*t *= 0.4531, df = 6). For both graphs, two-tailed, paired *t* test) (*N* = 7, *n* = 18). ***F***, Circular plots representing the phase relationship between ipsilateral FE (L2 vs L5) (*left panel*) and LR side of the cord (*right panel*) during local stimulation (L1/L2) in 3 µM NMDA (light gray; FE, *N* = 6, *n* = 15; LR, *N* = 8, *n* = 23) and 6 µM NMDA (dark gray; FE, *N* = 7, *n* = 15; LR, *N* = 6, *n* = 16). ***G***, Schematic of the experimental setup for local light stimulation of the lower lumbar cord (L5/L6). ***H***, Example of rhythmic activity before, during and after local stimulation (L5/L6; 20 s) in 3 µM NMDA and 8 µM 5-HT. ***I***, Quantification of frequency (*left panel*), burst amplitude (*right panel*), and burst duration (*middle panel*) of the locomotor-like activity recorded at L5 (local stimulation (L5/L6); 3 µM NMDA and 8 µM 5-HT) (*Left panel*, frequency, *p *= 0.9603 baseline vs light (*t *= 0.05398, df = 3), *p *= 0.5780 baseline vs post-light (*t *= 0.6220, df = 3). *Middle panel*, amplitude, *p *= 0.3688 baseline vs light (*t *= 1.055, df = 3), *p *= 0.8425 (*t *= 0.2164, df = 3) baseline vs post-light. *Right panel*, burst duration, *p* = 0.2522 baseline vs light (*t *= 1.414, df = 3), *p* = 0.0820 baseline vs post-light (*t *= 2.577, df = 3). For all graphs, two-tailed, paired *t* test) (*N* = 4, *n* = 9). ***J***, Example of rhythmic activity before, during and after local stimulation (L5/L6) in 6 µM NMDA and 8 µM 5-HT. ***K***, Quantification of frequency (*left panel*), burst amplitude (*middle panel*), and burst duration (*right panel*) of locomotor-like activity (local stimulation (L5/L6); 6 µM NMDA and 8 µM 5-HT) (*Left panel*, frequency, *p *= 0.6694 baseline vs light (*t *= 0.4716, df = 3), *p *= 0.0590 baseline vs post-light (*t *= 2.972, df = 3), two-tailed, paired *t* test. *Middle panel*, amplitude, *p *= 0.25 baseline vs light (*W* = 8), *p *= 0.6250 baseline vs post-light (*W* = 4), two-tailed, Wilcoxon's matched-pairs signed rank test. *Right panel*, burst duration, *p* = 0.2430 baseline vs light (*t *= 1.450, df = 3), *p* = 0.2867 baseline vs post-light (*t *= 1.293, df = 3), two-tailed, paired *t* test) (*N* = 4, *n* = 8). ***L***, Circular plots representing the phase relationship between ipsilateral FE (L2 vs L5) (*left panel*) and LR side of the cord (*right panel*) during local stimulation (L5/L6) in 3 µM NMDA (light gray; FE, *N* = 4, *n* = 7; LR, *N* = 4, *n* = 9) and 6 µM NMDA (dark gray; FE, *N* = 3, *n* = 6; LR, *N* = 4, *n* = 8). For all graphs, gray lines and dots represent the mean for each spinal cord (mean of 2–3 trials); black lines and dots represent the overall mean ± SD. For all ventral roots recording, blue shaded area is indicating when the light is ON. All recordings were done between P0 and P3. D, dorsal; V, ventral; R, rostral; C, caudal.

The changes in frequencies were more pronounced when stimulating the Lhx9-derived neurons in the upper lumbar cord (L1/L2) (at 3 µM NMDA: *N* = 9, *n* = 25 and at 6 µM NMDA: *N* = 7, *n* = 18) (Fig. 11*A–C* (left panel), Fig. 11*D,E* (left panel)). This was also the case for the increase in burst amplitude (Fig. 11*C*, middle panel) and the decrease of the burst duration (Fig. 11*C*, right panel) at 3 µM NMDA. However, at 6 µM NMDA, the burst amplitude was decreased after light activation of Lhx9-derived neurons (Fig. 11*E*, middle panel) and a slight reduction in burst duration (Fig. 11*E*, right panel).

Since the broad stimulation was less efficient than the local stimulation of the L1/L2, we also tested local stimulation of lower lumbar cord (L5/L6). Interestingly, local stimulation of Lhx9-derived neurons in the lower lumbar cord at any drug-induced locomotor-like frequency did not induce a visible effect on the locomotor-like frequency, the burst amplitude, or the burst duration (at 3 µM NMDA, *N* = 4, *n* = 9 and at 6 µM NMDA, *N* = 4, *n* = 8) (Fig. 11*G–L*).

As when locomotor activity was increased from a non-rhythmic baseline condition, the change of frequency of the on-going locomotor-like activity during light stimulation was accompanied by flexor–extensor alternation (broad stimulation (L1–L6), 3 µM: *N* = 5, *n* = 13 and 6 µM: *N* = 7, *n* = 15; local stimulation (L1/L2), 3 µM: *N* = 6, *n* = 15 and 6 µM: *N* = 7, *n* = 15; local stimulation (L5/L6), 3 µM: *N* = 4, *n* = 7 and 6 µM: *N* = 3, *n* = 6)) as well as left–right alternation (broad stimulation (L1–L6), 3 µM: *N* = 7, *n* = 18 and 6 µM: *N* = 7, *n* = 15; local stimulation (L1/L2), 3 µM: *N* = 8, *n* = 23 and 6 µM: *N* = 6, *n* = 16; local stimulation (L5/L6), 3 µM: *N* = 4, *n* = 9 and 6 µM: *N* = 4, *n* = 8) (Figs. 10*F*, 11*F,L*).

In summary the optogenetic experiments show that activation of Lhx9-derived neurons can induce locomotor-like activity from a non-rhythmic baseline condition and significantly increase the frequency of the on-going locomotor-like activity with preserved left–right and flexor–extensor alternation.

### Lhx9-derived neurons in the upper lumbar spinal cord are rhythmically active during locomotor-like activity

**Figure 12.**
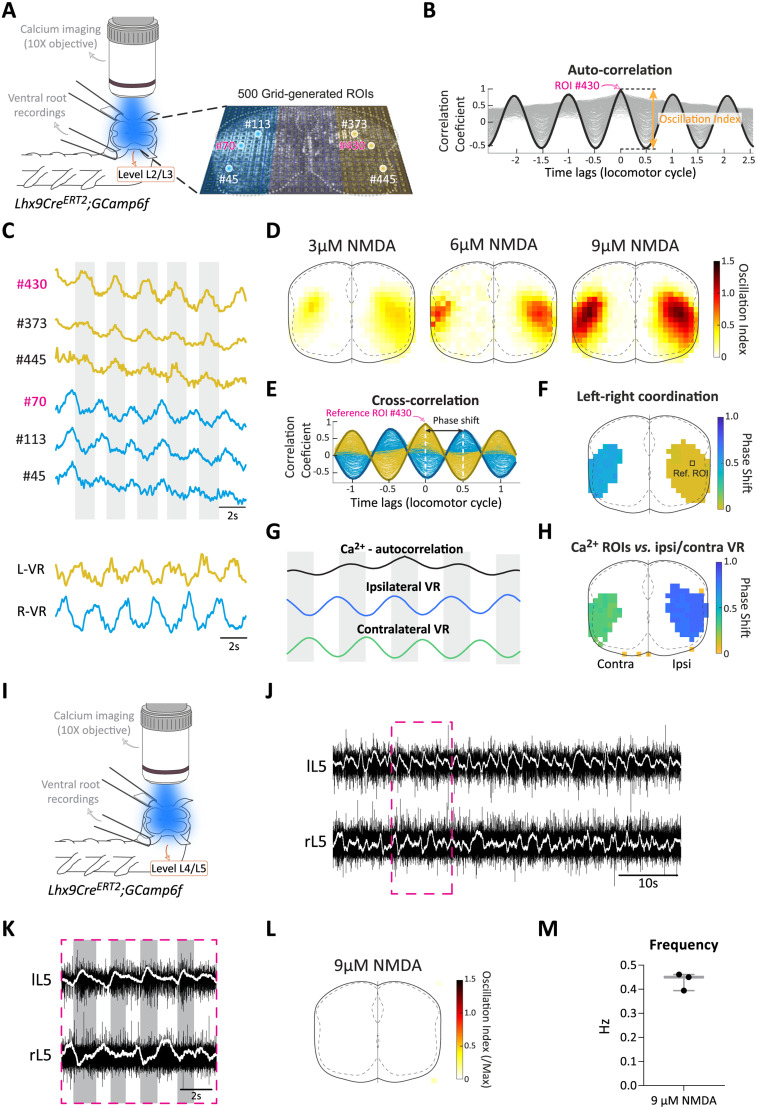
Lhx9-derived neurons in the upper lumbar cord are rhythmically active during on-going locomotor activity with higher calcium-activity at higher frequencies. ***A***, *Left panel*, Experimental setup for calcium (Ca^2+^) imaging in the upper lumbar in *Lhx9Cre^ERT2^;Gcamp6f* neonatal mice (P0–P3). *Right panel*, Frame of the Ca^2+^ signal (fluorescence transients, ΔF) where the field is divided into 500-grid-generated ROIs. Blue shaded area represents the left side of the lumbar cord and yellow shaded area represents the right side of the lumbar cord. Numbers in white and corresponding dots represent areas where the Ca^2+^ activity is relatively weak compared to the numbers in magenta and corresponding dots where Ca^2+^ is relatively high. Magenta numbers correspond to the location of the calcium signal. ***B***, *Upper panel*, Examples of Ca^2+^ signals (9 µM NMDA) extracted from individual grid-ROIs (see (***A***) for location of the traces) where the traces with a magenta number represent signals with the relative higher Ca^2+^ activity. Yellow traces represent the activity of the right side of the cord while blue traces represent the activity on the left side. *Lower panel*, Rectified version of the ventral root (VR) recordings in the left (L) and right (R) side of the cord at the level of the cut (9 µM NMDA). ***C***, Autocorrelation analysis of the Ca^2+^ signal in individual ROIs. Gray lines indicate traces for individual ROIs/ROI #430. Black line indicates the mean. ***D***, Heatmap representing the mean Ca^2+^ activity of the individual grid-ROIs with different oscillation index at different NMDA concentrations (3 µM, *N* = 3 spinal cords; 6 µM, *N* = 5; 9 µM, *N* = 5). ***E***, Cross-correlation analysis showing the phase relationship between left (blue) and right (yellow) side of the cord. Thin blue and yellow lines represent traces for individual ROIs/ROI#430 and thicker blue and yellow lines represent the mean. ***F***, Heatmap showing the phase relationship between the left (blue) and right (yellow) side of the upper lumbar cord (9 µM NMDA). The phase analysis shows a strict alternation between the left and right side with 1 representing the out-of-phase and 0 representing in-phase as compared to the reference ROI (ref ROI #430). ***G***, A representative map of phase shift between one individual Ca^2+^ ROIs versus ipsilateral and contralateral ventral root (VR). ***H***, A representative map of phase shift between 500 Ca^2+^ ROIs versus ipsilateral (ipsi) and contralateral (contra) ventral roots. ***I***, Experimental setup for calcium (Ca^2+^) imaging in the lower lumbar (L4/L5) in *Lhx9Cre^ERT2^;Gcamp6f* neonatal mice (P0–P3). ***J***, Example of ventral root recordings on the left (l) and right (r) side at L5 (9 µM NMDA + 8 µM 5-HT). ***K***, Magnified boxed area (pink dash line) of the raw recording. ***L***, Heatmap representing the mean Ca^2+^ activity in the lower lumbar spinal cord (L4) (*N* = 3). There is no rhythmic Ca^2+^ activity detected, therefore the heatmap will show as “blank/empty” heatmap. ***M***, Quantification of the frequency in 9 µM NMDA to show that the drugs induce rhythmicity.

Given that the Lhx9Cre neurons may be involved in modulating the frequency of the rhythm, we eventually also set out to determine the Lhx9-derived neuronal activity during locomotor-like activity. For this, we crossed *Lhx9Cre^ERT2^* mice with homozygous *Gcamp6f* mice which lead to the expression of the Ca^2+^ indicator in Lhx9Cre neurons. To directly visualize the Ca^2+^ activity in the cord (P0–P3), we performed a coronal cut at the level L2/L3 and bent the cord at a 90-degree angle to face the 10× microscope objective (Fig. 12*A*, left panel) which allowed us to the visualize and record the Ca^2+^ activity in the entire transverse section (Fig. 12*A*, right panel). Motor activity was monitored by ventral root recordings. To induce different locomotor-like frequency, we used NMDA concentrations of 3 µM, 6 µM and 9 µM) with a fixed-concentration of 5-HT (8 µM) [(mean ± SD for all); 3 µM = 0.21 ± 0.025 Hz (*N* = 3), 6 µM = 0.34 ± 0.012 Hz (*N* = 5), 9 µM = 0.43 ± 0.027 Hz (*N* = 5)].

We employed a set of 500 grid-based regions of interest (grid-ROIs) that covered the entire transverse section of the spinal cord (Fig. 12*A*, right panel). From these ROIs, we extracted the fluorescent transient (Δ*F*) of each individual ROI. The ROIs with the highest change in Ca^2+^ activities (ROIs with magenta number) extend from the dorsolateral part of the intermediate lamina into the deep dorsal horn where the Lhx9-derived neuronal cell bodies are positioned in the lumbar cord (Fig. 12*C*) (#70, #430), which is similar to the expression pattern results (Fig. 2*B*). Rhythmic Ca^2+^ signal with a weaker intensity was also seen outside the area of Lhx9-derived neuron cell body position. These signals are likely corresponding to activity in Cre-derived terminals fields (e.g., close to the motor neuron polls in the ventral horn (#445, #45) or dendrites of Lhx9-derived neurons (#113, #373)).

To correlate the rhythmic activity of individual grid-ROIs, we conducted an autocorrelation analysis (Fig. 12*B*). This analysis provided an oscillatory index, represented by the peak to trough correlation coefficient, for each ROI (Fig. 12*B*). Notably, there was a strong positive correlation between the frequency of locomotor-like activity and the oscillation index (Fig. 12*D,E*). In addition, the area of peak rhythmically active ROIs increased in size-intensity as the frequency increased, also in what appears to be the terminal areas (Fig. 12*D*). These frequency-dependent changes indicate stronger activation of already activated neurons and/or involvement of a larger number of Lhx9-derived neurons at higher frequencies.

When the Ca^2+^ activity is correlated with the ventral root activity on the same side, it appears that the active ROIs (activity in the Lhx9-derived neurons) on one side of the cord are mostly in-phase with the ipsilateral roots and out-of-phase with the contralateral roots (Fig. 12*G,H*).

To investigate the phase relationship between the left and right side of the cord, we performed a cross-correlation analysis of ROIs (Fig. 12*E*). This analysis showed that activity in the ROIs on one side of the cord, at the peak of their activity, are in strict alternation (phase shifts of 0.5) to ROIs on the other side of the cord (Fig. 12*F* and Movie 1).

10.1523/JNEUROSCI.1607-23.2024.video1 Movie 1 Calcium imaging of Lhx9-derived cells in the upper lumbar spinal cord Calcium imaging of GCamp6f-Lhx9 labeled cells (*upper panel*) and activity of the corresponding ventral roots (*lower panel*) recorded in the upper lumbar (L2) during locomotor-like activity induced by 8 µM 5HT and 9 µM NMDA in the neonatal mouse. Download Movie 1, MP4 file.

Notably, when we looked at the Ca^2+^ activity in the lower lumbar cord (L4) at 9 µM NMDA (*N* = 3), Lhx9-derived neurons did not seem to be rhythmically active at all as the oscillation index is next-to-zero (Fig. 12*I–M*). This finding indicates that Lhx9-derived neurons contribute rhythmically to flexor-related activity in the upper as upper lumbar cord, but with little contribution to extensor rhythmicity in the lower lumbar cord.

In summary, these results show that Lhx9-derived neurons are rhythmically active in a frequency-dependent manner but only in the upper lumbar cord during drug-induced locomotor activity and that the activity is correlated to activity in the ipsilateral root and are strictly alternating between left and right side of the cord.

## Discussion

This work adds to the molecular characterization of spinal glutamatergic neurons. Our results characterize a distinct group of ipsilaterally projecting excitatory spinal population, expressing the transcription factor Lhx9, which appears to play a role in modulating the frequency of locomotion and consequently suggesting a collaborative role in the rhythm generation circuits with other neuronal populations in the spinal cord.

Our study employed RNA sequencing and differential expression analysis to characterize molecular markers for spinal glutamatergic neurons. This approach was formulated to ensure that we captured markers in a subpopulation of glutamatergic neurons that may contribute to rhythm generation. For this we first evaluated transcriptomes in Vglut2-GFP^+^ cells — after transcripts from non-Vglut2 cells were subtracted — and then made a differential expression analysis with the Shox2Cre reporter cells. The detection of expected cohort-specific genes for ventrally located spinal glutamatergic neurons in the Vglut2-GFP^+^ cells like, Sim1, Evx2, Lhx3, Vsx2, Isl1, Lbx1, and Shox2 as well as other transcripts found in glutamatergic cells in the spinal cord (Hayashi et al., 2018; Rosenberg et al., 2018; Sathyamurthy et al., 2018; Baek et al., 2019; Russ et al., 2021) indicate that our method is specific enough to define glutamatergic cell population. Moreover, the Shox2 population transcript also reflects well-known genes detected in Shox2^+^ cells in the spinal cord (Dougherty et al., 2013; Hayashi et al., 2018; Russ et al., 2021). We therefore believe that our differential expression analysis identifies a broad population of glutamatergic cells (Vglut2-GFP^+^/Shox2Cre;Rosa26-YFP^−^) in the spinal cord characterized by a range of differentially upregulated TFs such as Lhx9, Ebf2, Ebf1, BarhL2, Dmbx1, Suv39H2, Ebf3, Sp7, Rbpj, Meox1, St18, Nkx2-8, Hmga1, Foxb1, Kank1, Onecut2, Id3, Nfkbib, Atrx, and Neurod1. Among these, Lhx9 is the most upregulated TF in the Vglut2-GFP^+^/Shox2Cre;Rosa26-YFP^−^ population and additionally when we performed anatomical expression analysis, we found a restricted expression pattern of Lhx9 together with Dmbx1, Neurod1, and Bahrl2 in the motor area of the spinal cord. In a first attempt we therefore here focused on Lhx9-derived cells.

Lhx9 has previously been shown to be expressed by dorsally derived dl1 neurons (Wilson et al., 2008; Ding et al., 2012). dl1 neurons give rise to two subgroups of neurons in the spinal cord during development based on the level of expression of Lhx2 and Lhx9: the contralaterally (mostly expressing Lhx2) and the ipsilaterally projecting dl1 neurons (expressing Lhx9) (Wilson et al., 2008; Ding et al., 2012). Here, we show that Lhx9-derived neurons are all glutamatergic and have almost exclusively ipsilateral and local projections composed of descending and ascending axons in the lumbar spinal cord. Notably, this population does not overlap with major populations of ventral located excitatory spinal neurons like the Shox2 and Chx10 neurons. Moreover, the glutamatergic Hb9Cre-derived neurons are scattered in the ventral horn (Caldeira et al., 2017) with little regional overlap with the Lhx9-derived neurons. Finally, the VSCT neurons are in the most ventral part of the spinal cord close to the motor neurons (Chalif et al., 2022) in a position that exclude an overlap with Lhx9 neurons. The Lhx9 neurons, therefore, define a distinct glutamatergic population in the lumbar spinal cord whose physiological role in the network has not been previously evaluated.

A main finding is that Lhx9 cells regulate the locomotor frequency. Thus, chronic silencing of the excitatory output from Lhx9-derived neurons or acute silencing of Lhx9-derived neurons reduce the frequency of drug-induced locomotor-like activity. In contrast, silencing had no effect on patterning, including left–right and flexor–extensor alternations, unlike what has been described for the manipulation of excitatory V2a (Chx10^+^/Shox2^−^ and Chx10^+^/Shox2^+^), V0 or V3 neurons (Crone et al., 2008, 2009; Talpalar et al., 2013; Zhang et al., 2015). Additionally, optogenetic activation of Lhx9-derived neurons induces rhythmic locomotor-like activity from a non-rhythmic baseline condition and increases the frequency of the drug-induced locomotor-like activity. With these features the Lhx9 population is fulfilling the main criteria of being part of the rhythm generating circuits which is that a selective reduction in their number should reduce the frequency of the on-going locomotor rhythm, and their activation should be able to influence the locomotor frequency by establishing and/or regulating it without affecting flexor and extensor coordination (Grillner and Jessell, 2009; Hägglund et al., 2010, 2013; Roberts et al., 2010; Dougherty et al., 2013; Ampatzis et al., 2014; McLean and Dougherty, 2015; Kiehn, 2016; Haque and Gosgnach, 2019). Alternatively, rather than contributing to rhythm generating circuits per se the Lhx9 cells could be providing an external drive to the rhythm generating circuits and thereby being able to modulate the frequency bidirectionally. To distinguish between these roles for the Lhx9-derived cells — as frequency modulator or rhythm generating cells — are in itself challenging with activation and inactivation experiments. The fact that we were unable to evoke rhythmic activity from rest without increasing the general excitability may speak against a direct role of the Lhx9-derived cells in rhythm generation but could also be explained by insufficient expression of channelrhodopsin in recombined cells. We made a similar observation using Vglut2 as a (weak) promoter to directly induce ChR2 expression in all glutamatergic neurons. In that case, too, it was necessary to enhance overall excitability in the spinal cord to successfully induce rhythmic activity through optogenetics (Hägglund et al., 2010). Notably, previous studies have shown a similar-size reduction of the locomotor frequency as seen here with chronic blunting of the excitatory transmission in the Shox2 population (Dougherty et al., 2013) or in the broader excitatory Hb9 population (Caldeira et al., 2017) unlike specifically ablating the synaptic transmission in the canonical small Hb9 population which had no effect on the rhythm (Koronfel et al., 2021). Clearly, silencing any one of the broad excitatory Hb9, the Shox2 or the Lhx9 populations is not sufficient to abolish the rhythm. This contrasts with optogenetic inhibition of all glutamatergic neurons in the spinal cord which completely abolished the rhythm (Hägglund et al., 2013). Since there is no cellular overlap of these three populations, the findings suggest that the rhythm generation in the neonatal mouse spinal cord engage several neuronal populations that together contribute to setting or modulating the frequency of the locomotor-like activity. A distribution of rhythm generation capability among cell populations has also been described in zebrafish (McLean and Fetcho, 2009; Ampatzis et al., 2014). Recent experiments have suggested that the activity in ventral spinocerebellar tract (VSCT) neurons is necessary for locomotor generation as evoked by ventral and dorsal root stimulation in the neonatal mouse preparation. These findings are not easily aligned with the findings presented here and with our previous experiments on Shox2 and Hb9 neurons. However, the study also shows that VSCT neurons are electrically connected to motor neurons in neonatal mice. Therefore, inactivation of VSCT neuron activity may function as a sink and directly affect motor neuron activity, potentially blocking the output. Since the VSCTs are last-order neurons their role as rhythm generating circuits is not directly compatible with a model that separates rhythm and pattern, as suggested for the limbed locomotor network (Kiehn, 2016; Dougherty and Ha, 2019). Moreover, the VSCT neurons are connected to other spinal neurons and their activation may therefore promote locomotion through these neurons. Notably, the study on VSCT neurons did not investigate the frequency modulation of the on-going rhythm which is an essential condition for neurons to be rhythm generating. From the in vitro experiments, it is evident that VSCT neuron activity may influence locomotor-like activity, but based on the available evidence it is hard to assign an ultimate role of these neurons as being the only rhythm generating circuits, as suggested by the authors.

Interestingly, the influence on regulating the rhythm from the Lhx9 population is dependent on its rostro-caudal distribution in the lumbar spinal cord. It is only optogenetic activation of Lhx9-derived neurons in upper lumbar spinal cord that initiates locomotor-like activity or regulates the frequency. Activation of Lhx9-derived neurons in the lower lumbar spinal cord does not. This is unlike activating the entire glutamatergic population in the caudal lumbar spinal cord, which despite the well-known rostro-caudal rhythmogenic gradient shown in the spinal cord (Kjaerulff and Kiehn, 1996; Cowley and Schmidt, 1997; Kremer and Lev-Toy, 1997) is able to evoke a rhythmic activity (Hägglund et al., 2013). These findings point to a functional diversification of rostrally and caudally located Lhx9 cells, which may be reflected in the dominance of descending versus ascending projections in Lhx9 cells located in the upper lumbar cord as compared to the Lhx9 cells located in the lower lumbar cord. It is possible that this diversity is supported by a further molecular subdivision of the Lhx9 population (Russ et al., 2021). The functional diversification between rostrally and caudally located Lhx9 cells was also seen in the calcium imaging experiments. Only cells located in the upper lumbar cord showed rhythmicity drug-induced locomotor activity. This rhythmicity was expressed as rigorous alternation between the left and right side of the cord. In addition, there was a frequency dependent modulation of these neurons as higher frequency that led to activation of a larger number and/or stronger modulation of Lhx9-derived neurons. Notably, the activity was in phase with ipsilateral flexor related ventral root activity. The calcium signal did not precede the recorded ventral root signal, which could speak against a role for the Lhx9 cells in directly driving motor neuron activity. However, the apparent time delay could be because the recorded ventral root is at least a segment more rostral than the recorded calcium signal and that the calcium signal also has a slower time course than the ventral root recording. Therefore, the peak of cellular activity in the Lhx9 neurons might be closer to the peak motor neuron activity in the same segment than appreciated from the recordings presented here.

Removal or suppression of inhibitory V1 population activity also modulates the frequency of locomotor-like activity in the neonatal mice (Gosgnach et al., 2006; Falgairolle and O'Donovan, 2019, 2021) and the locomotor frequency in the adult mouse (Allodi et al., 2021). Does that mean that these V1 neurons should also be considered part of the rhythm generating circuit? We do not think so. In contrast to rhythm generating circuits that in all species investigated have been shown to be excitatory and be able to initiate or increase the frequency of the rhythm, V1 inhibitory neurons and their activity cannot evoke rhythmic activity or increase the frequency. The suggested role of the V1 population on the locomotor frequency must be indirect by modulating alternation between flexor and extensor rhythm generating circuitries in the spinal cord rather than being the core rhythm generating circuits (Shevtsova and Rybak, 2016; Falgairolle and O’Donovan, 2021) similar to what is found in the zebrafish spinal cords (Kimura and Higashijima, 2019). Therefore, the inhibitory V1 neurons appear to have a different role than the excitatory frequency modulating neurons.

### Conclusion

In summary, our study reveals additional molecular markers to those previously described for functional classes of excitatory subpopulations in the motor area of the rodent spinal cord. Of these the Lhx9 neurons encapsulate a distinct class of excitatory neurons which we show are involved in modulating or generating the locomotor rhythm. Our work together with previous work suggests that glutamatergic neurons in the rodent spinal cord spanning several classes of molecularly defined groups are involved in these functions.
