# Supplementary figures and images for: Excitatory Spinal Lhx9-Derived Interneurons Modulate Locomotor Frequency in Mice
Source: J Neurosci. 2024 Mar 4;44(18):e1607232024. doi: 10.1523/JNEUROSCI.1607-23.2024 (PMC11063822; doi:10.1523/JNEUROSCI.1607-23.2024)

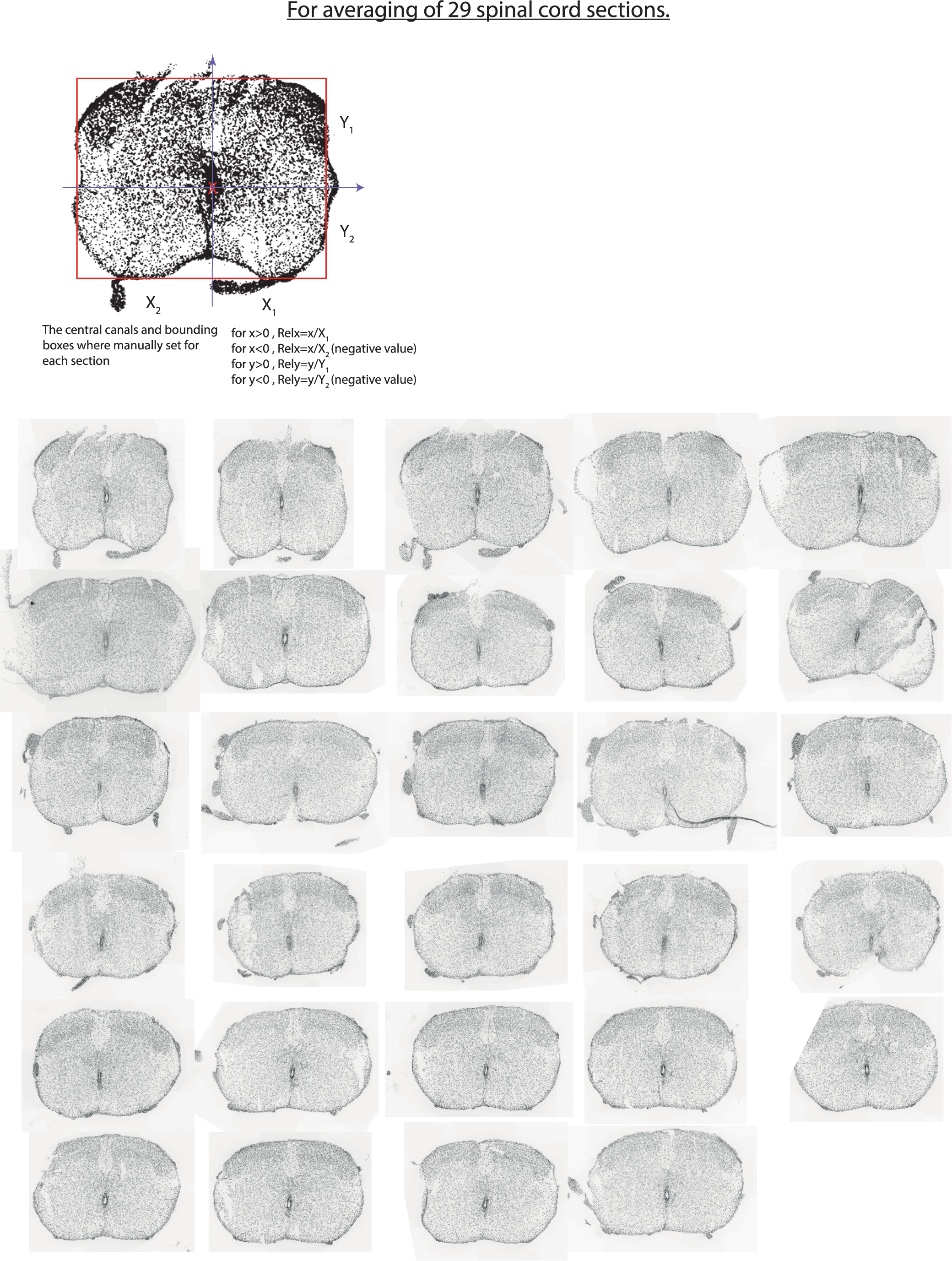

Supplement: Figure 1-1 — Section averaging for In Situ Sequencing (ISS) ISS on 29 sections from 5 spinal cords (P2). Upper panel: Description of spinal cord normalization. All spinal cords are normalized to the central canal (red X) and their maximal edge on each side (left, right, dorsal, ventral) to create a bounding box (square with red line) for each section for each spinal cord. This creates four areas, X1, X2 and Y1, Y2 which allows all cell positions to be normalized. This normalization allowed for an overlay of each section per transcript leading to the visualization of the local expression of each transcript within the spinal cord (see Figure 1-2). Lower panel: Sections from 5 different mice (5 to 4 sections for each animal) harvested at P2. Sections from the L2 spinal cord. Download Figure 1-1, TIF file. [file jneuro-44-e1607232024-s001.tif]

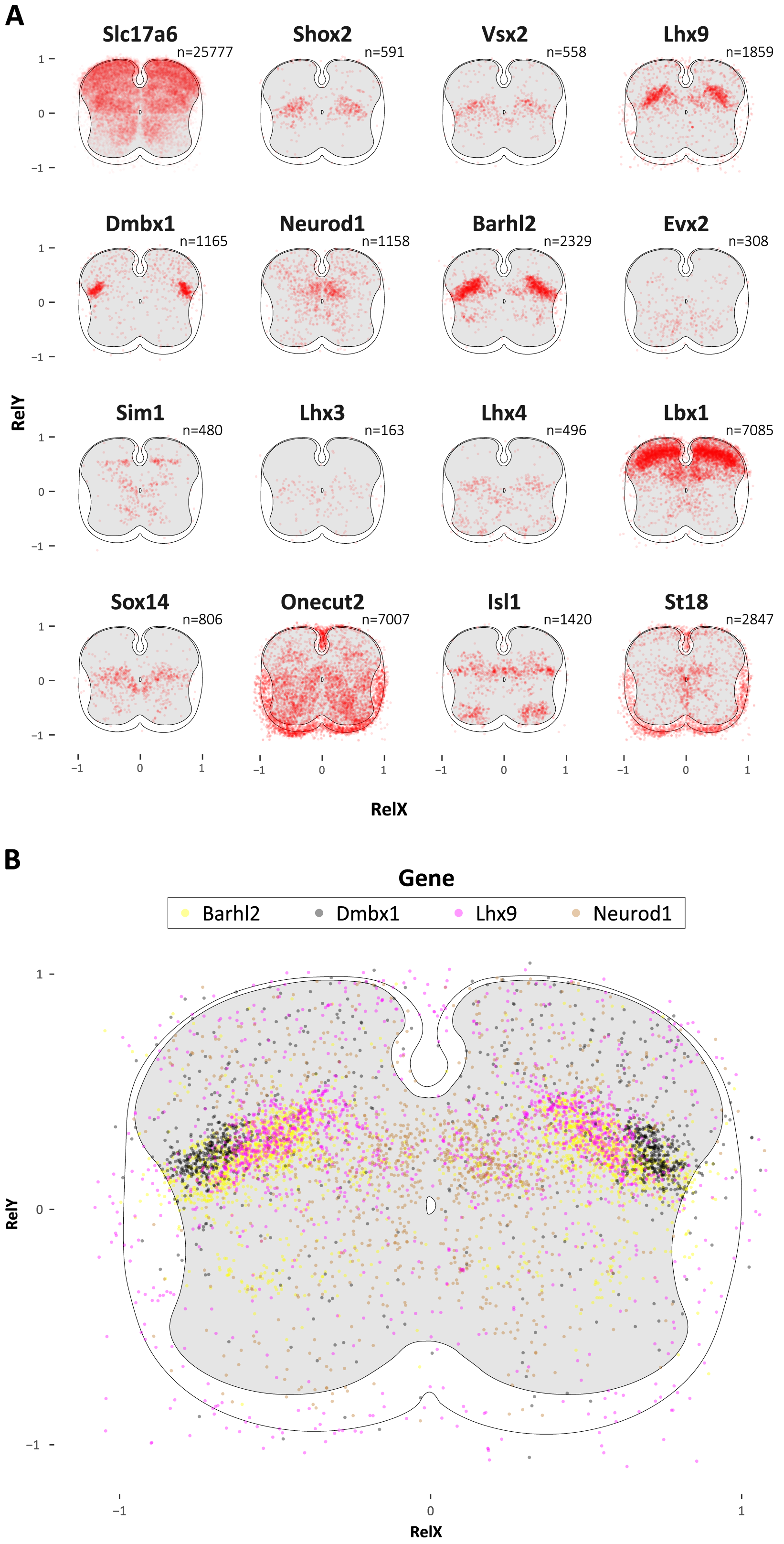

Supplement: Figure 1-2 — Spatial visualization of excitatory transcripts in the lumbar cord after ISS. A. Expression pattern of 16 excitatory transcripts in the lumbar cord (P2) after ISS. These transcripts have either a precise or a broad expression pattern. Shox2, Vsx2, Lhx3, Lhx4, Shox14, Islt1 and Zic2 represent the Shox2Cre;Rosa26-YFP+ population. The Vglut2-GFP+/Shox2Cre;Rosa26-YFP- population is represented by Lhx9, Barhl2, Dmbx1, Neurod1, Onecut2 and St18 transcripts. Sim1 and Lbx1 represent other excitatory populations. Alpha values for the dots were 0.12 except for Slc17a6 which was 0.03. The n number represents the total number of transcripts found in the 29 sections from 5 different mice. Note that for all probes there is some background that leads to a spurious expression pattern. B. Overlay of the expression pattern of 4 excitatory transcripts: Barhl2 (yellow), Dmbx1 (dark grey), Lhx9 (magenta) and Neurod1 (light brown). These represent the Vglut2-GFP+/Shox2Cre;Rosa26-YFP- population. All transcripts seem to have a unique expression pattern with little overlap with each other. The Lhx9 expression pattern of the transcript corresponds to the tdTomato expression seen in Lhx9CreERT2;Rosa26-tdTomato neonatal mice (see Figure 2). All probes present some background expression with outside the main expression area in the grey matter. Download Figure 1-2, TIF file. [file jneuro-44-e1607232024-s002.tif]
