# Supplementary material for: Excitatory Spinal Lhx9-Derived Interneurons Modulate Locomotor Frequency in Mice
Source: J Neurosci. 2024 Mar 4;44(18):e1607232024. doi: 10.1523/JNEUROSCI.1607-23.2024 (PMC11063822; doi:10.1523/JNEUROSCI.1607-23.2024)
Supplement: Table 1-1 — Differentially expressed transcription factors up-regulated in Vglut2-GFP+ cells (Vglut2-GFP+ vs. Vglut2-GFP- analysis) List of the differentially expressed transcription factors up-regulated in Vglut2-GFP+ cells. Download Table 1-1, DOCX file. [file jneuro-44-e1607232024-s004.docx]

Table 1-1. Differentially expressed transcription factors up-regulated in Vglut2-GFP^+^ cells (Vglut2-GFP^+^ *vs*. Vglut2-GFP^-^ analysis)

List of the differentially expressed transcription factors up-regulated in Vglut2-GFP^+^ cells.

|  | Symbol | Ensembl ID | Gene Name | log2FC | padj |
| --- | --- | --- | --- | --- | --- |
| 1 | Shox2 | ENSMUSG00000027833 | short stature homeobox 2 | 5,487 | 7,81E-23 |
| 2 | Lhx9 | ENSMUSG00000019230 | LIM homeobox 9 | 4,841 | 2,74E-20 |
| 3 | Ebf2 | ENSMUSG00000022053 | EBF transcription factor 2 | 4,624 | 2,46E-63 |
| 4 | Evx2 | ENSMUSG00000001815 | even-skipped homeobox 2 | 4,516 | 1,21E-07 |
| 5 | Sim1 | ENSMUSG00000019913 | SIM bHLH transcription factor 1 | 4,405 | 6,05E-11 |
| 6 | Onecut2 | ENSMUSG00000045991 | one cut homeobox 2 | 4,33 | 2,1E-104 |
| 7 | Bnc2 | ENSMUSG00000028487 | basonuclin 2 | 4,218 | 3,81E-42 |
| 8 | Skor2 | ENSMUSG00000091519 | SKI family transcriptional corepressor 2 | 3,922 | 2E-07 |
| 9 | Ebf3 | ENSMUSG00000010476 | EBF transcription factor 3 | 3,758 | 9,18E-38 |
| 10 | Olig3 | ENSMUSG00000045591 | oligodendrocyte transcription factor 3 | 3,706 | 1,58E-07 |
| 11 | Vsx2 | ENSMUSG00000021239 | visual system homeobox 2 | 3,576 | 4,57E-13 |
| 12 | Dlx2 | ENSMUSG00000023391 | distal-less homeobox 2 | 3,491 | 0,000135 |
| 13 | Sp7 | ENSMUSG00000060284 | Sp7 transcription factor | 3,452 | 1,97E-07 |
| 14 | Barhl2 | ENSMUSG00000034384 | BarH like homeobox 2 | 3,447 | 1,83E-05 |
| 15 | Isl1 | ENSMUSG00000042258 | ISL LIM homeobox 1 | 3,399 | 7,4E-08 |
| 16 | Pou4F1 | ENSMUSG00000048349 | POU class 4 homeobox 1 | 3,307 | 3,19E-25 |
| 17 | Lbx1 | ENSMUSG00000025216 | ladybird homeobox 1 | 3,167 | 2,01E-05 |
| 18 | Nfe2L3 | ENSMUSG00000029832 | nuclear factor, erythroid 2 like 3 | 3,143 | 7,86E-20 |
| 19 | Lhx3 | ENSMUSG00000026934 | LIM homeobox 3 | 3,104 | 0,00141 |
| 20 | Barx2 | ENSMUSG00000032033 | BARX homeobox 2 | 3,076 | 1,23E-12 |
| 21 | Lmx1B | ENSMUSG00000038765 | LIM homeobox transcription factor 1 beta | 3,044 | 0,000119 |
| 22 | En2 | ENSMUSG00000039095 | engrailed homeobox 2 | 2,988 | 1,64E-05 |
| 23 | Lhx1 | ENSMUSG00000018698 | LIM homeobox 1 | 2,969 | 7,49E-24 |
| 24 | Hoxb9 | ENSMUSG00000020875 | homeobox B9 | 2,838 | 1,06E-20 |
| 25 | Pou4F2 | ENSMUSG00000031688 | POU class 4 homeobox 2 | 2,754 | 0,0018 |
| 26 | Hes7 | ENSMUSG00000023781 | hes family bHLH transcription factor 7 | 2,749 | 0,00387 |
| 27 | Lhx2 | ENSMUSG00000000247 | LIM homeobox 2 | 2,698 | 0,000103 |
| 28 | Ebf1 | ENSMUSG00000057098 | EBF transcription factor 1 | 2,653 | 1,78E-18 |
| 29 | Nkx2-8 | ENSMUSG00000058669 | NK2 homeobox 8 | 2,604 | 0,00961 |
| 30 | Hoxd1 | ENSMUSG00000042448 | homeobox D1 | 2,579 | 0,00594 |
| 31 | Barhl1 | ENSMUSG00000026805 | BarH like homeobox 1 | 2,578 | 0,00597 |
| 32 | Npas4 | ENSMUSG00000045903 | neuronal PAS domain protein 4 | 2,553 | 0,000732 |
| 33 | Neurod6 | ENSMUSG00000037984 | neuronal differentiation 6 | 2,535 | 3,95E-38 |
| 34 | Nkx6-2 | ENSMUSG00000041309 | NK6 homeobox 2 | 2,533 | 0,00217 |
| 35 | Spdef | ENSMUSG00000024215 | SAM pointed domain containing ETS transcription factor | 2,464 | 2,7E-07 |
| 36 | Mitf | ENSMUSG00000035158 | melanocyte inducing transcription factor | 2,396 | 1,43E-12 |
| 37 | Myrf | ENSMUSG00000036098 | myelin regulatory factor | 2,353 | 2,25E-37 |
| 38 | Nhlh2 | ENSMUSG00000048540 | nescient helix-loop-helix 2 | 2,274 | 6,63E-07 |
| 39 | Smad7 | ENSMUSG00000025880 | SMAD family member 7 | 2,267 | 8,87E-25 |
| 40 | Bhlhe22 | ENSMUSG00000025128 | basic helix-loop-helix family member e22 | 2,177 | 6,21E-09 |
| 41 | Nhlh1 | ENSMUSG00000051251 | nescient helix-loop-helix 1 | 2,177 | 0,00596 |
| 42 | Hoxb8 | ENSMUSG00000056648 | homeobox B8 | 2,145 | 0,00117 |
| 43 | Hoxc13 | ENSMUSG00000001655 | homeobox C13 | 2,125 | 0,00231 |
| 44 | Ell2 | ENSMUSG00000001542 | elongation factor for RNA polymerase II 2 | 2,121 | 3,33E-43 |
| 45 | St18 | ENSMUSG00000033740 | ST18 C2H2C-type zinc finger transcription factor | 2,111 | 8,21E-52 |
| 46 | Runx1T1 | ENSMUSG00000006586 | RUNX1 partner transcriptional co-repressor 1 | 2,088 | 3,62E-08 |
| 47 | Pax2 | ENSMUSG00000004231 | paired box 2 | 2,085 | 6,24E-06 |
| 48 | Ankrd55 | ENSMUSG00000049985 | ankyrin repeat domain 55 | 2,06 | 3,38E-23 |
| 49 | Grhl1 | ENSMUSG00000020656 | grainyhead like transcription factor 1 | 2,057 | 1,64E-06 |
| 50 | Neurog2 | ENSMUSG00000027967 | neurogenin 2 | 2,04 | 0,0457 |
| 51 | Sox14 | ENSMUSG00000053747 | SRY-box transcription factor 14 | 2,003 | 0,0322 |
| 52 | Fhl2 | ENSMUSG00000008136 | four and a half LIM domains 2 | 2,001 | 7,09E-64 |
| 53 | Lhx5 | ENSMUSG00000029595 | LIM homeobox 5 | 1,929 | 0,00169 |
| 54 | Znf593 | ENSMUSG00000028840 | zinc finger protein 593 | 1,926 | 0,00327 |
| 55 | Hoxd10 | ENSMUSG00000050368 | homeobox D10 | 1,92 | 5,05E-24 |
| 56 | Tfeb | ENSMUSG00000023990 | transcription factor EB | 1,918 | 1,08E-05 |
| 57 | Ankrd34B | ENSMUSG00000045034 | ankyrin repeat domain 34B | 1,873 | 2,53E-13 |
| 58 | Zcchc12 | ENSMUSG00000036699 | zinc finger CCHC-type containing 12 | 1,86 | 5,9E-18 |
| 59 | Neurod1 | ENSMUSG00000034701 | neuronal differentiation 1 | 1,799 | 7,88E-10 |
| 60 | Hdac11 | ENSMUSG00000034245 | histone deacetylase 11 | 1,785 | 3,48E-14 |
| 61 | Zfp92 | ENSMUSG00000031374 | ZFP92 zinc finger protein | 1,77 | 0,000227 |
| 62 | Sirt2 | ENSMUSG00000015149 | sirtuin 2 | 1,706 | 2,73E-05 |
| 63 | Taf10 | ENSMUSG00000043866 | TATA-box binding protein associated factor 10 | 1,696 | 9,7E-07 |
| 64 | Hivep3 | ENSMUSG00000028634 | HIVEP zinc finger 3 | 1,688 | 8,82E-37 |
| 65 | Bend6 | ENSMUSG00000042182 | BEN domain containing 6 | 1,687 | 6,6E-12 |
| 66 | Klf14 | ENSMUSG00000073209 | Kruppel like factor 14 | 1,66 | 0,0227 |
| 67 | Lmo2 | ENSMUSG00000032698 | LIM domain only 2 | 1,614 | 5,83E-24 |
| 68 | Neurod2 | ENSMUSG00000038255 | neuronal differentiation 2 | 1,611 | 0,000398 |
| 69 | Trim66 | ENSMUSG00000031026 | tripartite motif containing 66 | 1,596 | 2,87E-07 |
| 70 | Rnf112 | ENSMUSG00000010086 | ring finger protein 112 | 1,572 | 7,9E-09 |
| 71 | Bcl11A | ENSMUSG00000000861 | BAF chromatin remodeling complex subunit BCL11A | 1,568 | 1,07E-09 |
| 72 | Cers2 | ENSMUSG00000015714 | ceramide synthase 2 | 1,55 | 2,78E-17 |
| 73 | Tfap2A | ENSMUSG00000021359 | transcription factor AP-2 alpha | 1,55 | 0,00929 |
| 74 | Zfhx2 | ENSMUSG00000040721 | zinc finger homeobox 2 | 1,547 | 1,6E-12 |
| 75 | Dip2A | ENSMUSG00000020231 | disco interacting protein 2 homolog A | 1,529 | 1,81E-14 |
| 76 | Phc1 | ENSMUSG00000040669 | polyhomeotic homolog 1 | 1,513 | 5,05E-59 |
| 77 | Zbtb8B | ENSMUSG00000048485 | zinc finger and BTB domain containing 8B | 1,505 | 1,55E-08 |
| 78 | Arid3B | ENSMUSG00000004661 | AT-rich interaction domain 3B | 1,5 | 1,04E-06 |
| 79 | Hr | ENSMUSG00000022096 | HR lysine demethylase and nuclear receptor corepressor | 1,489 | 7,88E-07 |
| 80 | Satb2 | ENSMUSG00000038331 | SATB homeobox 2 | 1,481 | 0,000329 |
| 81 | Lhx4 | ENSMUSG00000026468 | LIM homeobox 4 | 1,463 | 0,00428 |
| 82 | Myt1L | ENSMUSG00000061911 | myelin transcription factor 1 like | 1,444 | 3,07E-23 |
| 83 | Sall4 | ENSMUSG00000027547 | spalt like transcription factor 4 | 1,416 | 0,0375 |
| 84 | Hnf1B | ENSMUSG00000020679 | HNF1 homeobox B | 1,412 | 0,0334 |
| 85 | Cbfa2T3 | ENSMUSG00000006362 | CBFA2/RUNX1 partner transcriptional co-repressor 3 | 1,403 | 0,000384 |
| 86 | Ncor2 | ENSMUSG00000029478 | nuclear receptor corepressor 2 | 1,379 | 0,000264 |
| 87 | Hoxb3Os | ENSMUSG00000084844 | homeobox B3 and homeobox B2, opposite strand | 1,357 | 0,000271 |
| 88 | Bcl11B | ENSMUSG00000048251 | BAF chromatin remodeling complex subunit BCL11B | 1,354 | 7,91E-08 |
| 89 | Cited2 | ENSMUSG00000039910 | Cbp/p300 interacting transactivator with Glu/Asp rich carboxy-terminal domain 2 | 1,351 | 1,87E-06 |
| 90 | Lcorl | ENSMUSG00000015882 | ligand dependent nuclear receptor corepressor like | 1,306 | 2,09E-17 |
| 91 | Gbx1 | ENSMUSG00000067724 | gastrulation brain homeobox 1 | 1,303 | 0,0121 |
| 92 | Znf579 | ENSMUSG00000051550 | zinc finger protein 579 | 1,29 | 0,000836 |
| 93 | Jmy | ENSMUSG00000021690 | junction mediating and regulatory protein, p53 cofactor | 1,282 | 0,000212 |
| 94 | Nfil3 | ENSMUSG00000056749 | nuclear factor, interleukin 3 regulated | 1,274 | 3,65E-07 |
| 95 | Zbtb34 | ENSMUSG00000068966 | zinc finger and BTB domain containing 34 | 1,274 | 7,23E-12 |
| 96 | Ankrd45 | ENSMUSG00000044835 | ankyrin repeat domain 45 | 1,255 | 1,34E-15 |
| 97 | Tfap2B | ENSMUSG00000025927 | transcription factor AP-2 beta | 1,233 | 0,00216 |
| 98 | Pax8 | ENSMUSG00000026976 | paired box 8 | 1,23 | 0,0161 |
| 99 | Tcf7L1 | ENSMUSG00000055799 | transcription factor 7 like 1 | 1,225 | 1,64E-06 |
| 100 | Znf583 | ENSMUSG00000030443 | zinc finger protein 583 | 1,208 | 4,86E-10 |
| 101 | Tcf7L2 | ENSMUSG00000024985 | transcription factor 7 like 2 | 1,206 | 0,000412 |
| 102 | Tshz3 | ENSMUSG00000021217 | teashirt zinc finger homeobox 3 | 1,204 | 4,17E-10 |
| 103 | Ldb2 | ENSMUSG00000039706 | LIM domain binding 2 | 1,192 | 2,48E-13 |
| 104 | Ss18L1 | ENSMUSG00000039086 | SS18L1 subunit of BAF chromatin remodeling complex | 1,187 | 1,96E-10 |
| 105 | Med30 | ENSMUSG00000038622 | mediator complex subunit 30 | 1,181 | 5,11E-05 |
| 106 | Csrnp3 | ENSMUSG00000044647 | cysteine and serine rich nuclear protein 3 | 1,171 | 1,32E-18 |
| 107 | Ncoa7 | ENSMUSG00000039697 | nuclear receptor coactivator 7 | 1,171 | 1,22E-10 |
| 108 | Htatip2 | ENSMUSG00000039745 | HIV-1 Tat interactive protein 2 | 1,131 | 5,69E-09 |
| 109 | Zbtb38 | ENSMUSG00000040433 | zinc finger and BTB domain containing 38 | 1,112 | 0,0041 |
| 110 | Etv6 | ENSMUSG00000030199 | ETS variant transcription factor 6 | 1,11 | 1,19E-16 |
| 111 | Lcor | ENSMUSG00000025019 | ligand dependent nuclear receptor corepressor | 1,1 | 4,62E-18 |
| 112 | Zbtb43 | ENSMUSG00000026788 | zinc finger and BTB domain containing 43 | 1,1 | 1,94E-27 |
| 113 | Dach2 | ENSMUSG00000025592 | dachshund family transcription factor 2 | 1,091 | 8,5E-06 |
| 114 | Atxn7L3 | ENSMUSG00000059995 | ataxin 7 like 3 | 1,088 | 0,000019 |
| 115 | Klf9 | ENSMUSG00000033863 | Kruppel like factor 9 | 1,035 | 2,54E-14 |
| 116 | Hoxb6 | ENSMUSG00000000690 | homeobox B6 | 1,027 | 0,000903 |
| 117 | Actn2 | ENSMUSG00000052374 | actinin alpha 2 | 1,012 | 0,000754 |
| 118 | Maml3 | ENSMUSG00000061143 | mastermind like transcriptional coactivator 3 | 0,998 | 4,51E-05 |
| 119 | Rbfox2 | ENSMUSG00000033565 | RNA binding fox-1 homolog 2 | 0,992 | 4,76E-06 |
| 120 | Trank1 | ENSMUSG00000062296 | tetratricopeptide repeat and ankyrin repeat containing 1 | 0,988 | 0,00706 |
| 121 | Creb3L2 | ENSMUSG00000038648 | cAMP responsive element binding protein 3 like 2 | 0,985 | 1,64E-12 |
| 122 | Zfhx3 | ENSMUSG00000038872 | zinc finger homeobox 3 | 0,978 | 0,000101 |
| 123 | Zfhx4 | ENSMUSG00000025255 | zinc finger homeobox 4 | 0,977 | 0,004 |
| 124 | Npas2 | ENSMUSG00000026077 | neuronal PAS domain protein 2 | 0,972 | 0,0129 |
| 125 | Pknox1 | ENSMUSG00000006705 | PBX/knotted 1 homeobox 1 | 0,948 | 4,08E-07 |
| 126 | Bbx | ENSMUSG00000022641 | BBX high mobility group box domain containing | 0,939 | 5,3E-17 |
| 127 | Nfkbib | ENSMUSG00000030595 | NFKB inhibitor beta | 0,935 | 0,000765 |
| 128 | Kat2B | ENSMUSG00000000708 | lysine acetyltransferase 2B | 0,934 | 0,00252 |
| 129 | Pawr | ENSMUSG00000035873 | pro-apoptotic WT1 regulator | 0,921 | 0,0111 |
| 130 | Zbtb1 | ENSMUSG00000033454 | zinc finger and BTB domain containing 1 | 0,92 | 3,45E-06 |
| 131 | Noct | ENSMUSG00000023087 | nocturnin | 0,919 | 6,18E-05 |
| 132 | Klf13 | ENSMUSG00000052040 | Kruppel like factor 13 | 0,908 | 0,00287 |
| 133 | Siah2 | ENSMUSG00000036432 | siah E3 ubiquitin protein ligase 2 | 0,908 | 8,62E-06 |
| 134 | Tle5 | ENSMUSG00000054452 | TLE family member 5, transcriptional modulator | 0,907 | 0,000112 |
| 135 | Taf13 | ENSMUSG00000048100 | TATA-box binding protein associated factor 13 | 0,902 | 8,31E-16 |
| 136 | Zic1 | ENSMUSG00000032368 | Zic family member 1 | 0,899 | 0,00434 |
| 137 | Znf239 | ENSMUSG00000042097 | zinc finger protein 239 | 0,898 | 6,41E-10 |
| 138 | Zeb2 | ENSMUSG00000026872 | zinc finger E-box binding homeobox 2 | 0,89 | 3,62E-27 |
| 139 | Eid1 | ENSMUSG00000091337 | EP300 interacting inhibitor of differentiation 1 | 0,882 | 1,06E-06 |
| 140 | Kank2 | ENSMUSG00000032194 | KN motif and ankyrin repeat domains 2 | 0,87 | 0,00284 |
| 141 | Tsc22D4 | ENSMUSG00000029723 | TSC22 domain family member 4 | 0,852 | 0,000279 |
| 142 | Zic3 | ENSMUSG00000067860 | Zic family member 3 | 0,849 | 0,000976 |
| 143 | Crebzf | ENSMUSG00000051451 | CREB/ATF bZIP transcription factor | 0,842 | 0,00506 |
| 144 | Cdyl | ENSMUSG00000059288 | chromodomain Y like | 0,824 | 2,98E-05 |
| 145 | Tcf25 | ENSMUSG00000001472 | transcription factor 25 | 0,824 | 1,01E-10 |
| 146 | Asb1 | ENSMUSG00000026311 | ankyrin repeat and SOCS box containing 1 | 0,819 | 1,12E-09 |
| 147 | Smarca2 | ENSMUSG00000024921 | SWI/SNF related, matrix associated, actin dependent regulator of chromatin, subfamily a, member 2 | 0,796 | 1,58E-06 |
| 148 | Zbed5 | ENSMUSG00000034173 | zinc finger BED-type containing 5 | 0,783 | 0,0291 |
| 149 | Znf169 | ENSMUSG00000050954 | zinc finger protein 169 | 0,781 | 0,00298 |
| 150 | Basp1 | ENSMUSG00000045763 | brain abundant membrane attached signal protein 1 | 0,779 | 0,000068 |
| 151 | Rybp | ENSMUSG00000055763 | RING1 and YY1 binding protein | 0,773 | 0,00968 |
| 152 | Mecp2 | ENSMUSG00000031393 | methyl-CpG binding protein 2 | 0,77 | 8,61E-06 |
| 153 | Zbtb7C | ENSMUSG00000044646 | zinc finger and BTB domain containing 7C | 0,766 | 0,0165 |
| 154 | Znf605 | ENSMUSG00000023284 | zinc finger protein 605 | 0,761 | 0,000202 |
| 155 | Fem1B | ENSMUSG00000032244 | fem-1 homolog B | 0,756 | 8,69E-06 |
| 156 | Tshz1 | ENSMUSG00000046982 | teashirt zinc finger homeobox 1 | 0,747 | 0,000056 |
| 157 | Nkap | ENSMUSG00000016409 | NFKB activating protein | 0,745 | 1,88E-10 |
| 158 | Hoxd11 | ENSMUSG00000042499 | homeobox D11 | 0,737 | 0,00285 |
| 159 | Hoxb7 | ENSMUSG00000038721 | homeobox B7 | 0,733 | 0,000624 |
| 160 | Hdac2 | ENSMUSG00000019777 | histone deacetylase 2 | 0,728 | 0,000227 |
| 161 | Znf502 | ENSMUSG00000069184 | zinc finger protein 502 | 0,728 | 0,00079 |
| 162 | Tsc22D3 | ENSMUSG00000031431 | TSC22 domain family member 3 | 0,723 | 3,76E-13 |
| 163 | Brf1 | ENSMUSG00000011158 | BRF1 RNA polymerase III transcription initiation factor subunit | 0,722 | 2,95E-05 |
| 164 | Znf471 | ENSMUSG00000055150 | zinc finger protein 471 | 0,698 | 0,0222 |
| 165 | Zfp57 | ENSMUSG00000036036 | ZFP57 zinc finger protein | 0,695 | 0,0342 |
| 166 | Mycbp | ENSMUSG00000028647 | MYC binding protein | 0,682 | 1,11E-05 |
| 167 | Yaf2 | ENSMUSG00000022634 | YY1 associated factor 2 | 0,682 | 0,00908 |
| 168 | Mtf1 | ENSMUSG00000028890 | metal regulatory transcription factor 1 | 0,676 | 3,28E-11 |
| 169 | Ankrd46 | ENSMUSG00000048307 | ankyrin repeat domain 46 | 0,674 | 3,49E-09 |
| 170 | Elmsan1 | ENSMUSG00000042507 | ELM2 and Myb/SANT domain containing 1 | 0,671 | 0,00282 |
| 171 | Stat2 | ENSMUSG00000040033 | signal transducer and activator of transcription 2 | 0,671 | 0,00339 |
| 172 | Arid4B | ENSMUSG00000039219 | AT-rich interaction domain 4B | 0,667 | 0,000854 |
| 173 | Cnot7 | ENSMUSG00000031601 | CCR4-NOT transcription complex subunit 7 | 0,667 | 1,4E-08 |
| 174 | Taf11 | ENSMUSG00000024218 | TATA-box binding protein associated factor 11 | 0,658 | 4,5E-08 |
| 175 | Zbtb46 | ENSMUSG00000027583 | zinc finger and BTB domain containing 46 | 0,657 | 0,0108 |
| 176 | Jazf1 | ENSMUSG00000063568 | JAZF zinc finger 1 | 0,641 | 0,000171 |
| 177 | Pspc1 | ENSMUSG00000021938 | paraspeckle component 1 | 0,64 | 0,0181 |
| 178 | Tle1 | ENSMUSG00000008305 | TLE family member 1, transcriptional corepressor | 0,632 | 0,00453 |
| 179 | Polr3K | ENSMUSG00000038628 | RNA polymerase III subunit K | 0,628 | 6,18E-11 |
| 180 | Ssbp2 | ENSMUSG00000003992 | single stranded DNA binding protein 2 | 0,627 | 1,46E-05 |
| 181 | Dmrta1 | ENSMUSG00000043753 | DMRT like family A1 | 0,62 | 0,0367 |
| 182 | Znf18 | ENSMUSG00000018347 | zinc finger protein 18 | 0,619 | 0,0201 |
| 183 | Plagl1 | ENSMUSG00000019817 | PLAG1 like zinc finger 1 | 0,608 | 0,0206 |
| 184 | Kdm5B | ENSMUSG00000042207 | lysine demethylase 5B | 0,606 | 0,0285 |
| 185 | Smarca5 | ENSMUSG00000031715 | SWI/SNF related, matrix associated, actin dependent regulator of chromatin, subfamily a, member 5 | 0,598 | 0,0378 |
