# Supplementary material for: Excitatory Spinal Lhx9-Derived Interneurons Modulate Locomotor Frequency in Mice
Source: J Neurosci. 2024 Mar 4;44(18):e1607232024. doi: 10.1523/JNEUROSCI.1607-23.2024 (PMC11063822; doi:10.1523/JNEUROSCI.1607-23.2024)
Supplement: Table 1-2 — Differentially expressed transcription factors down-regulated in Vglut2-GFP+ cells (Vglut2-GFP+ vs. Vglut2-GFP- analysis) List of the differentially expressed transcription factors down-regulated in Vglut2-GFP+ cells. Download Table 1-2, DOCX file. [file jneuro-44-e1607232024-s005.docx]

Table 1-2. Differentially expressed transcription factors down-regulated in Vglut2-GFP^+^ cells (Vglut2-GFP^+^ vs. Vglut2-GFP^-^ analysis)

List of the differentially expressed transcription factors down-regulated in Vglut2-GFP^+^ cells.

|  | Symbol | Ensembl ID | Gene Name | log2FC | padj |
| --- | --- | --- | --- | --- | --- |
| 1 | Gsx1 | ENSMUSG00000053129 | GS homeobox 1 | -4,456 | 5,41E-09 |
| 2 | Irx6 | ENSMUSG00000031738 | iroquois homeobox 6 | -3,945 | 1,16E-05 |
| 3 | Foxd1 | ENSMUSG00000078302 | forkhead box D1 | -3,884 | 7,74E-09 |
| 4 | Tbx18 | ENSMUSG00000032419 | T-box transcription factor 18 | -3,653 | 3,09E-12 |
| 5 | Osr2 | ENSMUSG00000022330 | odd-skipped related transciption factor 2 | -3,536 | 0,000138 |
| 6 | Nupr1 | ENSMUSG00000030717 | nuclear protein 1, transcriptional regulator | -3,479 | 4E-44 |
| 7 | Ccnd1 | ENSMUSG00000070348 | cyclin D1 | -3,251 | 6,4E-43 |
| 8 | Osr1 | ENSMUSG00000048387 | odd-skipped related transcription factor 1 | -3,247 | 7,66E-07 |
| 9 | Sox3 | ENSMUSG00000045179 | SRY-box transcription factor 3 | -3,203 | 5,06E-14 |
| 10 | Ascl1 | ENSMUSG00000020052 | achaete-scute family bHLH transcription factor 1 | -3,186 | 3,27E-38 |
| 11 | Foxa1 | ENSMUSG00000035451 | forkhead box A1 | -3,103 | 0,000946 |
| 12 | Meox2 | ENSMUSG00000036144 | mesenchyme homeobox 2 | -3,055 | 5,5E-06 |
| 13 | Twist1 | ENSMUSG00000035799 | twist family bHLH transcription factor 1 | -3,047 | 3,77E-05 |
| 14 | Uhrf1 | ENSMUSG00000001228 | ubiquitin like with PHD and ring finger domains 1 | -2,968 | 2,63E-49 |
| 15 | Depdc1 | ENSMUSG00000028175 | DEP domain containing 1 | -2,821 | 3,56E-29 |
| 16 | Etv1 | ENSMUSG00000004151 | ETS variant transcription factor 1 | -2,78 | 1,32E-61 |
| 17 | Lef1 | ENSMUSG00000027985 | lymphoid enhancer binding factor 1 | -2,707 | 1,27E-27 |
| 18 | Prrx1 | ENSMUSG00000026586 | paired related homeobox 1 | -2,691 | 4,15E-52 |
| 19 | Mis18Bp1 | ENSMUSG00000047534 | MIS18 binding protein 1 | -2,658 | 3,4E-37 |
| 20 | Mxd3 | ENSMUSG00000021485 | MAX dimerization protein 3 | -2,641 | 3,81E-09 |
| 21 | Etv5 | ENSMUSG00000013089 | ETS variant transcription factor 5 | -2,611 | 5,14E-43 |
| 22 | Nab2 | ENSMUSG00000025402 | NGFI-A binding protein 2 | -2,55 | 0,000133 |
| 23 | Foxc1 | ENSMUSG00000050295 | forkhead box C1 | -2,542 | 2,91E-07 |
| 24 | Kcnip3 | ENSMUSG00000079056 | potassium voltage-gated channel interacting protein 3 | -2,518 | 1,31E-26 |
| 25 | Sox6 | ENSMUSG00000051910 | SRY-box transcription factor 6 | -2,515 | 1,55E-54 |
| 26 | Vav1 | ENSMUSG00000034116 | vav guanine nucleotide exchange factor 1 | -2,466 | 0,000005 |
| 27 | Mybl1 | ENSMUSG00000025912 | MYB proto-oncogene like 1 | -2,436 | 1,27E-09 |
| 28 | Dmrtb1 | ENSMUSG00000028610 | DMRT like family B with proline rich C-terminal 1 | -2,43 | 3,94E-11 |
| 29 | Tgfb1I1 | ENSMUSG00000030782 | transforming growth factor beta 1 induced transcript 1 | -2,422 | 2,01E-25 |
| 30 | Arx | ENSMUSG00000035277 | aristaless related homeobox | -2,377 | 0,00481 |
| 31 | Znf616 | ENSMUSG00000062518 | zinc finger protein 616 | -2,351 | 0,0218 |
| 32 | Myt1 | ENSMUSG00000010505 | myelin transcription factor 1 | -2,328 | 1,09E-55 |
| 33 | Irx1 | ENSMUSG00000060969 | iroquois homeobox 1 | -2,327 | 0,000709 |
| 34 | Tcf19 | ENSMUSG00000050410 | transcription factor 19 | -2,295 | 2,43E-09 |
| 35 | Gata3 | ENSMUSG00000015619 | GATA binding protein 3 | -2,274 | 1,18E-07 |
| 36 | Lmo1 | ENSMUSG00000036111 | LIM domain only 1 | -2,272 | 8,16E-25 |
| 37 | Rbpjl | ENSMUSG00000017007 | recombination signal binding protein for immunoglobulin kappa J region like | -2,242 | 4,74E-06 |
| 38 | Zeb1 | ENSMUSG00000024238 | zinc finger E-box binding homeobox 1 | -2,235 | 1,29E-42 |
| 39 | Ets1 | ENSMUSG00000032035 | ETS proto-oncogene 1, transcription factor | -2,218 | 1,07E-31 |
| 40 | Foxd2 | ENSMUSG00000055210 | forkhead box D2 | -2,217 | 0,0257 |
| 41 | Egr2 | ENSMUSG00000037868 | early growth response 2 | -2,21 | 1,24E-23 |
| 42 | Creb3L1 | ENSMUSG00000027230 | cAMP responsive element binding protein 3 like 1 | -2,209 | 6,58E-09 |
| 43 | Nkx6-1 | ENSMUSG00000035187 | NK6 homeobox 1 | -2,196 | 1,44E-14 |
| 44 | Rfx4 | ENSMUSG00000020037 | regulatory factor X4 | -2,191 | 1,46E-18 |
| 45 | Gfi1B | ENSMUSG00000026815 | growth factor independent 1B transcriptional repressor | -2,153 | 0,0327 |
| 46 | Runx2 | ENSMUSG00000039153 | RUNX family transcription factor 2 | -2,136 | 1,39E-08 |
| 47 | Zfp36L1 | ENSMUSG00000021127 | ZFP36 ring finger protein like 1 | -2,125 | 1,59E-20 |
| 48 | Snai1 | ENSMUSG00000042821 | snail family transcriptional repressor 1 | -2,096 | 0,000466 |
| 49 | Fhl5 | ENSMUSG00000028259 | four and a half LIM domains 5 | -2,089 | 0,0413 |
| 50 | Tbx3 | ENSMUSG00000018604 | T-box transcription factor 3 | -2,088 | 0,000397 |
| 51 | Hic1 | ENSMUSG00000043099 | HIC ZBTB transcriptional repressor 1 | -2,087 | 0,0103 |
| 52 | Foxc2 | ENSMUSG00000046714 | forkhead box C2 | -2,085 | 0,0033 |
| 53 | Foxm1 | ENSMUSG00000001517 | forkhead box M1 | -2,054 | 3,82E-21 |
| 54 | Tcf23 | ENSMUSG00000006642 | transcription factor 23 | -2,048 | 0,0418 |
| 55 | Cdkn2C | ENSMUSG00000028551 | cyclin dependent kinase inhibitor 2C | -2,03 | 4,56E-20 |
| 56 | Foxl1 | ENSMUSG00000097084 | forkhead box L1 | -2,028 | 0,0441 |
| 57 | Hmgb2 | ENSMUSG00000054717 | high mobility group box 2 | -2,018 | 4,43E-13 |
| 58 | Mybl2 | ENSMUSG00000017861 | MYB proto-oncogene like 2 | -2,01 | 5,68E-09 |
| 59 | Ostf1 | ENSMUSG00000024725 | osteoclast stimulating factor 1 | -1,941 | 1,84E-21 |
| 60 | Msx1 | ENSMUSG00000048450 | msh homeobox 1 | -1,931 | 4,94E-05 |
| 61 | Grhl2 | ENSMUSG00000022286 | grainyhead like transcription factor 2 | -1,879 | 1,99E-17 |
| 62 | Gata2 | ENSMUSG00000015053 | GATA binding protein 2 | -1,876 | 0,000352 |
| 63 | Rbl1 | ENSMUSG00000027641 | RB transcriptional corepressor like 1 | -1,869 | 8,35E-19 |
| 64 | Irf8 | ENSMUSG00000041515 | interferon regulatory factor 8 | -1,867 | 0,00156 |
| 65 | Sp140 | ENSMUSG00000070031 | SP140 nuclear body protein | -1,865 | 0,000155 |
| 66 | Sp110 | ENSMUSG00000070034 | SP110 nuclear body protein | -1,854 | 3,72E-06 |
| 67 | Zfp36L2 | ENSMUSG00000045817 | ZFP36 ring finger protein like 2 | -1,847 | 5,92E-15 |
| 68 | Wdhd1 | ENSMUSG00000037572 | WD repeat and HMG-box DNA binding protein 1 | -1,817 | 5,29E-15 |
| 69 | Cavin1 | ENSMUSG00000004044 | caveolae associated protein 1 | -1,815 | 1,16E-17 |
| 70 | Npas1 | ENSMUSG00000001988 | neuronal PAS domain protein 1 | -1,812 | 0,00636 |
| 71 | Irf1 | ENSMUSG00000018899 | interferon regulatory factor 1 | -1,811 | 6,74E-13 |
| 72 | Nkx2-2 | ENSMUSG00000027434 | NK2 homeobox 2 | -1,81 | 2,86E-22 |
| 73 | Six4 | ENSMUSG00000034460 | SIX homeobox 4 | -1,808 | 2,11E-07 |
| 74 | Bcl6 | ENSMUSG00000022508 | BCL6 transcription repressor | -1,796 | 5,21E-06 |
| 75 | Cdkn2B | ENSMUSG00000073802 | cyclin dependent kinase inhibitor 2B | -1,773 | 0,0146 |
| 76 | Nrarp | ENSMUSG00000078202 | NOTCH regulated ankyrin repeat protein | -1,765 | 1,39E-07 |
| 77 | Pml | ENSMUSG00000036986 | promyelocytic leukemia | -1,765 | 1,64E-12 |
| 78 | Irx2 | ENSMUSG00000001504 | iroquois homeobox 2 | -1,749 | 7,03E-09 |
| 79 | Pou3F2 | ENSMUSG00000095139 | POU class 3 homeobox 2 | -1,736 | 3,14E-07 |
| 80 | Tcea3 | ENSMUSG00000001604 | transcription elongation factor A3 | -1,735 | 0,000131 |
| 81 | Foxo4 | ENSMUSG00000042903 | forkhead box O4 | -1,677 | 0,0108 |
| 82 | Neurod4 | ENSMUSG00000048015 | neuronal differentiation 4 | -1,661 | 0,0342 |
| 83 | Nfatc1 | ENSMUSG00000033016 | nuclear factor of activated T cells 1 | -1,657 | 9,07E-19 |
| 84 | Lmx1A | ENSMUSG00000026686 | LIM homeobox transcription factor 1 alpha | -1,637 | 0,031 |
| 85 | Znf469 | ENSMUSG00000043903 | zinc finger protein 469 | -1,637 | 0,00499 |
| 86 | Zfp69 | ENSMUSG00000064141 | ZFP69 zinc finger protein | -1,631 | 7,35E-06 |
| 87 | Etv4 | ENSMUSG00000017724 | ETS variant transcription factor 4 | -1,616 | 8,43E-05 |
| 88 | Irf5 | ENSMUSG00000029771 | interferon regulatory factor 5 | -1,586 | 0,000358 |
| 89 | Sp100 | ENSMUSG00000026222 | nuclear antigen Sp100 | -1,574 | 0,00385 |
| 90 | Pmf1/Pmf1-Bglap | ENSMUSG00000028066 | polyamine modulated factor 1 | -1,559 | 0,000113 |
| 91 | Vdr | ENSMUSG00000022479 | vitamin D receptor | -1,529 | 0,00135 |
| 92 | E2F7 | ENSMUSG00000020185 | E2F transcription factor 7 | -1,523 | 2,81E-11 |
| 93 | Lbh | ENSMUSG00000024063 | LBH regulator of WNT signaling pathway | -1,519 | 0,00105 |
| 94 | Nfatc2 | ENSMUSG00000027544 | nuclear factor of activated T cells 2 | -1,516 | 2,92E-20 |
| 95 | Akna | ENSMUSG00000039158 | AT-hook transcription factor | -1,514 | 0,0122 |
| 96 | Psmc3Ip | ENSMUSG00000019303 | PSMC3 interacting protein | -1,513 | 5,36E-07 |
| 97 | Batf3 | ENSMUSG00000026630 | basic leucine zipper ATF-like transcription factor 3 | -1,505 | 0,00128 |
| 98 | Egr1 | ENSMUSG00000038418 | early growth response 1 | -1,503 | 2,98E-22 |
| 99 | Prdm5 | ENSMUSG00000029913 | PR/SET domain 5 | -1,503 | 7,61E-16 |
| 100 | Hes1 | ENSMUSG00000022528 | hes family bHLH transcription factor 1 | -1,501 | 2,43E-05 |
| 101 | Nfix | ENSMUSG00000001911 | nuclear factor I X | -1,493 | 1,83E-12 |
| 102 | Tal1 | ENSMUSG00000028717 | TAL bHLH transcription factor 1, erythroid differentiation factor | -1,49 | 0,0141 |
| 103 | Gli1 | ENSMUSG00000025407 | GLI family zinc finger 1 | -1,484 | 0,0406 |
| 104 | Irf6 | ENSMUSG00000026638 | interferon regulatory factor 6 | -1,48 | 0,00765 |
| 105 | Hes5 | ENSMUSG00000048001 | hes family bHLH transcription factor 5 | -1,478 | 0,00116 |
| 106 | Stat6 | ENSMUSG00000002147 | signal transducer and activator of transcription 6 | -1,476 | 1,01E-08 |
| 107 | Cebpd | ENSMUSG00000071637 | CCAAT enhancer binding protein delta | -1,468 | 0,00525 |
| 108 | Vezf1 | ENSMUSG00000018377 | vascular endothelial zinc finger 1 | -1,46 | 3,84E-20 |
| 109 | Bcl6B | ENSMUSG00000000317 | BCL6B transcription repressor | -1,457 | 0,000136 |
| 110 | Heyl | ENSMUSG00000032744 | hes related family bHLH transcription factor with YRPW motif like | -1,439 | 0,000274 |
| 111 | Glis1 | ENSMUSG00000034762 | GLIS family zinc finger 1 | -1,422 | 4,03E-07 |
| 112 | Sox5 | ENSMUSG00000041540 | SRY-box transcription factor 5 | -1,403 | 3,97E-21 |
| 113 | Nfia | ENSMUSG00000028565 | nuclear factor I A | -1,402 | 0,000823 |
| 114 | Elk3 | ENSMUSG00000008398 | ETS transcription factor ELK3 | -1,397 | 1,4E-10 |
| 115 | Gli2 | ENSMUSG00000048402 | GLI family zinc finger 2 | -1,383 | 0,0119 |
| 116 | Pou3F4 | ENSMUSG00000056854 | POU class 3 homeobox 4 | -1,37 | 7,46E-05 |
| 117 | Arid5A | ENSMUSG00000037447 | AT-rich interaction domain 5A | -1,367 | 0,00177 |
| 118 | Myb | ENSMUSG00000019982 | MYB proto-oncogene, transcription factor | -1,351 | 0,00799 |
| 119 | Sox1 | ENSMUSG00000096014 | SRY-box transcription factor 1 | -1,351 | 0,00583 |
| 120 | Irx3 | ENSMUSG00000031734 | iroquois homeobox 3 | -1,341 | 0,0244 |
| 121 | Tcf7 | ENSMUSG00000000782 | transcription factor 7, T cell specific | -1,331 | 1,11E-05 |
| 122 | Rfx2 | ENSMUSG00000024206 | regulatory factor X2 | -1,318 | 0,0163 |
| 123 | Fli1 | ENSMUSG00000016087 | Fli-1 proto-oncogene, ETS transcription factor | -1,31 | 1,1E-09 |
| 124 | Prdm1 | ENSMUSG00000038151 | PR/SET domain 1 | -1,301 | 0,0264 |
| 125 | Znf367 | ENSMUSG00000044934 | zinc finger protein 367 | -1,289 | 9,39E-14 |
| 126 | Sall3 | ENSMUSG00000024565 | spalt like transcription factor 3 | -1,285 | 3,44E-17 |
| 127 | Bard1 | ENSMUSG00000026196 | BRCA1 associated RING domain 1 | -1,279 | 3,98E-05 |
| 128 | Rai14 | ENSMUSG00000022246 | retinoic acid induced 14 | -1,276 | 4,4E-08 |
| 129 | Cand2 | ENSMUSG00000030319 | cullin associated and neddylation dissociated 2 (putative) | -1,271 | 5,18E-10 |
| 130 | Tgif1 | ENSMUSG00000047407 | TGFB induced factor homeobox 1 | -1,267 | 2,56E-08 |
| 131 | Ajuba | ENSMUSG00000022178 | ajuba LIM protein | -1,261 | 0,00183 |
| 132 | Tox | ENSMUSG00000041272 | thymocyte selection associated high mobility group box | -1,261 | 4,72E-07 |
| 133 | Bhlhe40 | ENSMUSG00000030103 | basic helix-loop-helix family member e40 | -1,25 | 8,45E-11 |
| 134 | Znf423 | ENSMUSG00000045333 | zinc finger protein 423 | -1,242 | 0,000695 |
| 135 | Hlf | ENSMUSG00000003949 | HLF transcription factor, PAR bZIP family member | -1,239 | 2,13E-06 |
| 136 | Klf12 | ENSMUSG00000072294 | Kruppel like factor 12 | -1,238 | 4,41E-08 |
| 137 | Hhex | ENSMUSG00000024986 | hematopoietically expressed homeobox | -1,232 | 0,0104 |
| 138 | Insm1 | ENSMUSG00000068154 | INSM transcriptional repressor 1 | -1,23 | 0,0322 |
| 139 | Znf14 | ENSMUSG00000074158 | zinc finger protein 14 | -1,215 | 0,00135 |
| 140 | Hoxc5 | ENSMUSG00000022485 | homeobox C5 | -1,212 | 4,18E-05 |
| 141 | Tp63 | ENSMUSG00000022510 | tumor protein p63 | -1,212 | 0,0386 |
| 142 | Yap1 | ENSMUSG00000053110 | Yes associated protein 1 | -1,206 | 2,82E-05 |
| 143 | Spi1 | ENSMUSG00000002111 | Spi-1 proto-oncogene | -1,193 | 0,0116 |
| 144 | Ccne1 | ENSMUSG00000002068 | cyclin E1 | -1,192 | 2,4E-06 |
| 145 | Myc | ENSMUSG00000022346 | MYC proto-oncogene, bHLH transcription factor | -1,183 | 2,79E-07 |
| 146 | Glis3 | ENSMUSG00000052942 | GLIS family zinc finger 3 | -1,177 | 6,53E-07 |
| 147 | Nfib | ENSMUSG00000008575 | nuclear factor I B | -1,176 | 4,25E-21 |
| 148 | Tp53 | ENSMUSG00000059552 | tumor protein p53 | -1,176 | 3,26E-18 |
| 149 | Tcf4 | ENSMUSG00000053477 | transcription factor 4 | -1,173 | 1,81E-12 |
| 150 | Znf462 | ENSMUSG00000060206 | zinc finger protein 462 | -1,156 | 3,99E-12 |
| 151 | Zc3H3 | ENSMUSG00000075600 | zinc finger CCCH-type containing 3 | -1,141 | 0,000229 |
| 152 | Lmo4 | ENSMUSG00000028266 | LIM domain only 4 | -1,138 | 7,31E-09 |
| 153 | Ppp1R13L | ENSMUSG00000040734 | protein phosphatase 1 regulatory subunit 13 like | -1,135 | 0,0274 |
| 154 | Tead2 | ENSMUSG00000030796 | TEA domain transcription factor 2 | -1,134 | 0,00107 |
| 155 | Ifi16 | ENSMUSG00000073489 | interferon gamma inducible protein 16 | -1,124 | 0,011 |
| 156 | Znf226 | ENSMUSG00000087598 | zinc finger protein 226 | -1,124 | 4,1E-06 |
| 157 | Helz2 | ENSMUSG00000027580 | helicase with zinc finger 2 | -1,117 | 0,0155 |
| 158 | Tcf12 | ENSMUSG00000032228 | transcription factor 12 | -1,108 | 9,06E-12 |
| 159 | Znf496 | ENSMUSG00000020472 | zinc finger protein 496 | -1,105 | 1,8E-10 |
| 160 | Supt3H | ENSMUSG00000038954 | SPT3 homolog, SAGA and STAGA complex component | -1,102 | 1,07E-06 |
| 161 | Nacc2 | ENSMUSG00000026932 | NACC family member 2 | -1,088 | 1,87E-07 |
| 162 | Gli3 | ENSMUSG00000021318 | GLI family zinc finger 3 | -1,075 | 9,42E-05 |
| 163 | Srf | ENSMUSG00000015605 | serum response factor | -1,059 | 1,88E-08 |
| 164 | Gabpa | ENSMUSG00000008976 | GA binding protein transcription factor subunit alpha | -1,057 | 3,05E-13 |
| 165 | Med7 | ENSMUSG00000020397 | mediator complex subunit 7 | -1,054 | 1,9E-06 |
| 166 | Klf2 | ENSMUSG00000055148 | Kruppel like factor 2 | -1,053 | 0,000677 |
| 167 | Pou6F1 | ENSMUSG00000009739 | POU class 6 homeobox 1 | -1,04 | 1,04E-08 |
| 168 | Hdac8 | ENSMUSG00000067567 | histone deacetylase 8 | -1,038 | 0,00157 |
| 169 | Cbx2 | ENSMUSG00000025577 | chromobox 2 | -1,026 | 0,0301 |
| 170 | Sap30 | ENSMUSG00000031609 | Sin3A associated protein 30 | -1,015 | 0,00135 |
| 171 | E2F6 | ENSMUSG00000057469 | E2F transcription factor 6 | -1,012 | 1,33E-05 |
| 172 | Med12 | ENSMUSG00000079487 | mediator complex subunit 12 | -1,012 | 9,67E-10 |
| 173 | Ikzf1 | ENSMUSG00000018654 | IKAROS family zinc finger 1 | -1,011 | 0,0215 |
| 174 | Cited1 | ENSMUSG00000051159 | Cbp/p300 interacting transactivator with Glu/Asp rich carboxy-terminal domain 1 | -1,006 | 0,00131 |
| 175 | E2F2 | ENSMUSG00000018983 | E2F transcription factor 2 | -1,003 | 0,00264 |
| 176 | Grhl3 | ENSMUSG00000037188 | grainyhead like transcription factor 3 | -1,001 | 0,00475 |
| 177 | Sertad3 | ENSMUSG00000055200 | SERTA domain containing 3 | -0,988 | 2,59E-08 |
| 178 | Per2 | ENSMUSG00000055866 | period circadian regulator 2 | -0,985 | 0,00035 |
| 179 | Calcoco1 | ENSMUSG00000023055 | calcium binding and coiled-coil domain 1 | -0,983 | 0,015 |
| 180 | Wtip | ENSMUSG00000036459 | WT1 interacting protein | -0,98 | 0,00452 |
| 181 | Brca2 | ENSMUSG00000041147 | BRCA2 DNA repair associated | -0,978 | 0,000163 |
| 182 | Dek | ENSMUSG00000021377 | DEK proto-oncogene | -0,977 | 3,23E-08 |
| 183 | Maml2 | ENSMUSG00000031925 | mastermind like transcriptional coactivator 2 | -0,975 | 0,00409 |
| 184 | Gmnn | ENSMUSG00000006715 | geminin DNA replication inhibitor | -0,972 | 3,39E-12 |
| 185 | Znf22 | ENSMUSG00000059878 | zinc finger protein 22 | -0,963 | 5,25E-14 |
| 186 | Zfp36 | ENSMUSG00000044786 | ZFP36 ring finger protein | -0,955 | 0,0289 |
| 187 | Olig1 | ENSMUSG00000046160 | oligodendrocyte transcription factor 1 | -0,945 | 0,014 |
| 188 | Sox21 | ENSMUSG00000061517 | SRY-box transcription factor 21 | -0,944 | 0,00496 |
| 189 | Tcf3 | ENSMUSG00000020167 | transcription factor 3 | -0,943 | 0,000632 |
| 190 | Six5 | ENSMUSG00000040841 | SIX homeobox 5 | -0,94 | 0,0373 |
| 191 | Ahrr | ENSMUSG00000021575 | aryl-hydrocarbon receptor repressor | -0,938 | 0,0156 |
| 192 | Hoxa11 | ENSMUSG00000038210 | homeobox A11 | -0,903 | 0,00307 |
| 193 | Mycn | ENSMUSG00000037169 | MYCN proto-oncogene, bHLH transcription factor | -0,897 | 4,78E-06 |
| 194 | Pold3 | ENSMUSG00000030726 | DNA polymerase delta 3, accessory subunit | -0,895 | 3,21E-12 |
| 195 | Epas1 | ENSMUSG00000024140 | endothelial PAS domain protein 1 | -0,893 | 0,00344 |
| 196 | Taf6 | ENSMUSG00000036980 | TATA-box binding protein associated factor 6 | -0,89 | 5,36E-14 |
| 197 | Pax6 | ENSMUSG00000027168 | paired box 6 | -0,886 | 0,00101 |
| 198 | Nfkb1 | ENSMUSG00000028163 | nuclear factor kappa B subunit 1 | -0,885 | 1,56E-07 |
| 199 | Atf3 | ENSMUSG00000026628 | activating transcription factor 3 | -0,882 | 0,000127 |
| 200 | E2F8 | ENSMUSG00000046179 | E2F transcription factor 8 | -0,881 | 4,2E-07 |
| 201 | Hdac9 | ENSMUSG00000004698 | histone deacetylase 9 | -0,879 | 0,00113 |
| 202 | Zbtb16 | ENSMUSG00000066687 | zinc finger and BTB domain containing 16 | -0,876 | 0,00509 |
| 203 | Sp1 | ENSMUSG00000001280 | Sp1 transcription factor | -0,875 | 0,00289 |
| 204 | Olig2 | ENSMUSG00000039830 | oligodendrocyte transcription factor 2 | -0,872 | 0,00131 |
| 205 | Elf4 | ENSMUSG00000031103 | E74 like ETS transcription factor 4 | -0,869 | 0,0262 |
| 206 | Znf143 | ENSMUSG00000061079 | zinc finger protein 143 | -0,864 | 1,01E-05 |
| 207 | Atf1 | ENSMUSG00000080968 | activating transcription factor 1 | -0,856 | 0,00266 |
| 208 | Pprc1 | ENSMUSG00000055491 | PPARG related coactivator 1 | -0,848 | 3,81E-05 |
| 209 | Pbx1 | ENSMUSG00000052534 | PBX homeobox 1 | -0,843 | 9,68E-07 |
| 210 | Pbxip1 | ENSMUSG00000042613 | PBX homeobox interacting protein 1 | -0,835 | 3,29E-05 |
| 211 | Zhx3 | ENSMUSG00000035877 | zinc fingers and homeoboxes 3 | -0,832 | 4,17E-05 |
| 212 | Ruvbl2 | ENSMUSG00000003868 | RuvB like AAA ATPase 2 | -0,829 | 0,0396 |
| 213 | Nfkbiz | ENSMUSG00000035356 | NFKB inhibitor zeta | -0,827 | 0,000686 |
| 214 | Tead1 | ENSMUSG00000055320 | TEA domain transcription factor 1 | -0,826 | 0,00693 |
| 215 | Cenpj | ENSMUSG00000064128 | centromere protein J | -0,817 | 0,00221 |
| 216 | Pcbd1 | ENSMUSG00000020098 | pterin-4 alpha-carbinolamine dehydratase 1 | -0,816 | 0,0189 |
| 217 | Smad5 | ENSMUSG00000021540 | SMAD family member 5 | -0,816 | 5,77E-07 |
| 218 | Nfic | ENSMUSG00000055053 | nuclear factor I C | -0,813 | 0,016 |
| 219 | Tgif2 | ENSMUSG00000062175 | TGFB induced factor homeobox 2 | -0,808 | 0,000316 |
| 220 | Znf454 | ENSMUSG00000048728 | zinc finger protein 454 | -0,807 | 0,000464 |
| 221 | Aff1 | ENSMUSG00000029313 | AF4/FMR2 family member 1 | -0,801 | 0,00439 |
| 222 | Rfc1 | ENSMUSG00000029191 | replication factor C subunit 1 | -0,793 | 2,83E-06 |
| 223 | Hey1 | ENSMUSG00000040289 | hes related family bHLH transcription factor with YRPW motif 1 | -0,792 | 0,000635 |
| 224 | Wwtr1 | ENSMUSG00000027803 | WW domain containing transcription regulator 1 | -0,791 | 0,0299 |
| 225 | Tsc22D1 | ENSMUSG00000022010 | TSC22 domain family member 1 | -0,786 | 2,34E-06 |
| 226 | Pax3 | ENSMUSG00000004872 | paired box 3 | -0,783 | 0,00766 |
| 227 | Foxo1 | ENSMUSG00000044167 | forkhead box O1 | -0,78 | 8,24E-06 |
| 228 | Zscan12 | ENSMUSG00000036721 | zinc finger and SCAN domain containing 12 | -0,761 | 0,00451 |
| 229 | Mybbp1A | ENSMUSG00000040463 | MYB binding protein 1a | -0,758 | 0,00368 |
| 230 | Kdm3A | ENSMUSG00000053470 | lysine demethylase 3A | -0,751 | 2,38E-23 |
| 231 | Zbtb40 | ENSMUSG00000060862 | zinc finger and BTB domain containing 40 | -0,751 | 0,0467 |
| 232 | Zfp30 | ENSMUSG00000047473 | ZFP30 zinc finger protein | -0,75 | 0,000705 |
| 233 | Hey2 | ENSMUSG00000019789 | hes related family bHLH transcription factor with YRPW motif 2 | -0,749 | 0,00216 |
| 234 | Znf646 | ENSMUSG00000049739 | zinc finger protein 646 | -0,746 | 0,0315 |
| 235 | Zscan22 | ENSMUSG00000054715 | zinc finger and SCAN domain containing 22 | -0,728 | 0,00306 |
| 236 | Gabpb2 | ENSMUSG00000038766 | GA binding protein transcription factor subunit beta 2 | -0,715 | 0,00185 |
| 237 | Meis2 | ENSMUSG00000027210 | Meis homeobox 2 | -0,708 | 0,000226 |
| 238 | Ebf4 | ENSMUSG00000053552 | EBF family member 4 | -0,704 | 0,0306 |
| 239 | Pou3F3 | ENSMUSG00000045515 | POU class 3 homeobox 3 | -0,703 | 0,00703 |
| 240 | Hmgn1 | ENSMUSG00000040681 | high mobility group nucleosome binding domain 1 | -0,702 | 0,0163 |
| 241 | Tcfl5 | ENSMUSG00000038932 | transcription factor like 5 | -0,702 | 0,0376 |
| 242 | Cxxc1 | ENSMUSG00000024560 | CXXC finger protein 1 | -0,7 | 0,00872 |
| 243 | Sertad1 | ENSMUSG00000008384 | SERTA domain containing 1 | -0,698 | 0,048 |
| 244 | Sin3A | ENSMUSG00000042557 | SIN3 transcription regulator family member A | -0,692 | 1,38E-06 |
| 245 | Tox3 | ENSMUSG00000043668 | TOX high mobility group box family member 3 | -0,692 | 0,00125 |
| 246 | Msl3 | ENSMUSG00000031358 | MSL complex subunit 3 | -0,681 | 0,000693 |
| 247 | Pir | ENSMUSG00000031379 | pirin | -0,679 | 0,00538 |
| 248 | Taf9B | ENSMUSG00000047242 | TATA-box binding protein associated factor 9b | -0,674 | 6,67E-06 |
| 249 | Ilf3 | ENSMUSG00000032178 | interleukin enhancer binding factor 3 | -0,668 | 0,0207 |
| 250 | Eed | ENSMUSG00000030619 | embryonic ectoderm development | -0,659 | 7,76E-05 |
| 251 | Creb5 | ENSMUSG00000053007 | cAMP responsive element binding protein 5 | -0,655 | 0,00407 |
| 252 | Sox8 | ENSMUSG00000024176 | SRY-box transcription factor 8 | -0,654 | 1,14E-06 |
| 253 | Stat3 | ENSMUSG00000004040 | signal transducer and activator of transcription 3 | -0,652 | 0,0156 |
| 254 | Gm21596/Hmgb1 | ENSMUSG00000096006 | high mobility group box 1 | -0,649 | 0,000119 |
| 255 | Fos | ENSMUSG00000021250 | Fos proto-oncogene, AP-1 transcription factor subunit | -0,64 | 0,00134 |
| 256 | Cntrl | ENSMUSG00000057110 | centriolin | -0,639 | 0,00026 |
| 257 | Pknox2 | ENSMUSG00000035934 | PBX/knotted 1 homeobox 2 | -0,625 | 6,57E-09 |
| 258 | Znf566 | ENSMUSG00000078768 | zinc finger protein 566 | -0,622 | 0,0024 |
| 259 | Prdm15 | ENSMUSG00000014039 | PR/SET domain 15 | -0,617 | 1,56E-05 |
| 260 | Znf438 | ENSMUSG00000050945 | zinc finger protein 438 | -0,614 | 0,00835 |
| 261 | Irf9 | ENSMUSG00000002325 | interferon regulatory factor 9 | -0,607 | 0,000019 |
| 262 | Znf595 | ENSMUSG00000057842 | zinc finger protein 595 | -0,604 | 0,0326 |
| 263 | Btg2 | ENSMUSG00000020423 | BTG anti-proliferation factor 2 | -0,601 | 0,00835 |
| 264 | Cbx5 | ENSMUSG00000009575 | chromobox 5 | -0,596 | 0,00107 |
| 265 | Zfat | ENSMUSG00000022335 | zinc finger and AT-hook domain containing | -0,596 | 0,0401 |
