# Supplementary material for: Excitatory Spinal Lhx9-Derived Interneurons Modulate Locomotor Frequency in Mice
Source: J Neurosci. 2024 Mar 4;44(18):e1607232024. doi: 10.1523/JNEUROSCI.1607-23.2024 (PMC11063822; doi:10.1523/JNEUROSCI.1607-23.2024)
Supplement: Table 1-3 — Differentially expressed transcription factors up-regulated in Shox2Cre;Rosa26-YFP+ cells (Vglut2-GFP+ vs. Shox2Cre;Rosa26-YFP+ analysis) List of the differentially expressed transcription factors up-regulated in Shox2Cre;Rosa26-YFP+ cells. Download Table 1-3, DOCX file. [file jneuro-44-e1607232024-s006.docx]

Table 1-3. Differentially expressed transcription factors up-regulated in Shox2Cre;Rosa26-YFP^+^ cells (Vglut2-GFP^+^ *vs.* Shox2Cre;Rosa26-YFP^+^ analysis)

List of the differentially expressed transcription factors up-regulated in Shox2Cre;Rosa26-YFP^+^ cells.

|  | Symbol | Ensembl ID | Gene Name | log2FC | padj |
| --- | --- | --- | --- | --- | --- |
| 1 | Shox2 | ENSMUSG00000027833 | short stature homeobox 2 | -3,485 | 2,51E-38 |
| 2 | Foxd3 | ENSMUSG00000067261 | forkhead box D3 | -2,768 | 3,86E-11 |
| 3 | Erg | ENSMUSG00000040732 | ERG, ETS transcription factor | -2,664 | 8,49E-11 |
| 4 | Asb1 | ENSMUSG00000026311 | ankyrin repeat and SOCS box containing 1 | -2,576 | 1,2E-154 |
| 5 | Zic2 | ENSMUSG00000061524 | Zic family member 2 | -2,242 | 4,22E-07 |
| 6 | Pou3F1 | ENSMUSG00000090125 | POU domain, class 3, transcription factor 1 | -2,154 | 7,58E-20 |
| 7 | Lhx4 | ENSMUSG00000026468 | LIM homeobox 4 | -2,096 | 3,47E-15 |
| 8 | Isl1 | ENSMUSG00000042258 | ISL LIM homeobox 1 | -2,051 | 5,44E-06 |
| 9 | Foxf2 | ENSMUSG00000038402 | forkhead box F2 | -1,989 | 4,87E-06 |
| 10 | Lhx3 | ENSMUSG00000026934 | LIM homeobox 3 | -1,953 | 5,5E-07 |
| 11 | Sox7 | ENSMUSG00000063060 | SRY-box 7 | -1,85 | 0,000026 |
| 12 | Vsx2 | ENSMUSG00000021239 | visual system homeobox 2 | -1,849 | 1,39E-12 |
| 13 | Maf | ENSMUSG00000055435 | MAF bZIP transcription factor | -1,761 | 1,45E-14 |
| 14 | Fosl2 | ENSMUSG00000029135 | FOS like 2, AP-1 transcription factor subunit | -1,715 | 8,38E-09 |
| 15 | Sox14 | ENSMUSG00000053747 | SRY-box 14 | -1,643 | 1,39E-05 |
| 16 | Smyd1 | ENSMUSG00000055027 | SET and MYND domain containing 1 | -1,537 | 0,000736 |
| 17 | Prdm8 | ENSMUSG00000035456 | PR/SET domain 8 | -1,509 | 2,13E-05 |
| 18 | Tbx2 | ENSMUSG00000000093 | T-box 2 | -1,478 | 0,00231 |
| 19 | Pou4F2 | ENSMUSG00000031688 | POU class 4 homeobox 2 | -1,459 | 0,00195 |
| 20 | Foxq1 | ENSMUSG00000038415 | forkhead box Q1 | -1,444 | 0,00308 |
| 21 | Mafb | ENSMUSG00000074622 | MAF bZIP transcription factor B | -1,389 | 4,06E-05 |
| 22 | Klf4 | ENSMUSG00000003032 | Kruppel like factor 4 | -1,318 | 2,22E-14 |
| 23 | Fosb | ENSMUSG00000003545 | FosB proto-oncogene, AP-1 transcription factor subunit | -1,259 | 2,5E-09 |
| 24 | Hexim1 | ENSMUSG00000048878 | hexamethylene bisacetamide inducible 1 | -1,246 | 4,07E-05 |
| 25 | Klf5 | ENSMUSG00000005148 | Kruppel like factor 5 | -1,217 | 0,00912 |
| 26 | Tead4 | ENSMUSG00000030353 | TEA domain transcription factor 4 | -1,208 | 0,0158 |
| 27 | Hoxd11 | ENSMUSG00000042499 | homeobox D11 | -1,119 | 0,000263 |
| 28 | Atxn1 | ENSMUSG00000046876 | ataxin 1 | -1,113 | 5,4E-13 |
| 29 | Foxo3 | ENSMUSG00000048756 | forkhead box O3 | -1,094 | 2,77E-09 |
| 30 | Klf16 | ENSMUSG00000035397 | Kruppel-like factor 16 | -1,084 | 0,00697 |
| 31 | Cbfa2T3 | ENSMUSG00000006362 | CBFA2/RUNX1 translocation partner 3 | -1,081 | 0,000862 |
| 32 | Asb4 | ENSMUSG00000042607 | ankyrin repeat and SOCS box containing 4 | -1,073 | 0,00136 |
| 33 | Tfcp2L1 | ENSMUSG00000026380 | transcription factor CP2 like 1 | -1,048 | 0,0102 |
| 34 | E2F1 | ENSMUSG00000027490 | E2F transcription factor 1 | -1,039 | 0,00122 |
| 35 | Mllt1 | ENSMUSG00000024212 | MLLT1, super elongation complex subunit | -1,011 | 2,01E-05 |
| 36 | Baz2A | ENSMUSG00000040054 | bromodomain adjacent to zinc finger domain 2A | -0,928 | 0,000014 |
| 37 | Zfp57 | ENSMUSG00000036036 | ZFP57 zinc finger protein | -0,927 | 7,52E-07 |
| 38 | Prdm16 | ENSMUSG00000039410 | PR/SET domain 16 | -0,923 | 0,0123 |
| 39 | Cers6 | ENSMUSG00000027035 | ceramide synthase 6 | -0,901 | 0,000041 |
| 40 | Ski | ENSMUSG00000029050 | SKI proto-oncogene | -0,889 | 0,000313 |
| 41 | Tox2 | ENSMUSG00000074607 | TOX high mobility group box family member 2 | -0,888 | 0,0191 |
| 42 | Zic3 | ENSMUSG00000067860 | Zic family member 3 | -0,877 | 0,0145 |
| 43 | Plag1 | ENSMUSG00000003282 | PLAG1 zinc finger | -0,833 | 0,0115 |
| 44 | Zfhx3 | ENSMUSG00000038872 | zinc finger homeobox 3 | -0,791 | 0,0014 |
| 45 | Mnt | ENSMUSG00000000282 | MAX network transcriptional repressor | -0,778 | 0,00169 |
| 46 | Kmt2D | ENSMUSG00000048154 | lysine methyltransferase 2D | -0,777 | 1,79E-06 |
| 47 | Xbp1 | ENSMUSG00000020484 | X-box binding protein 1 | -0,771 | 0,000332 |
| 48 | Maml3 | ENSMUSG00000061143 | mastermind like transcriptional coactivator 3 | -0,743 | 0,0389 |
| 49 | Crebl2 | ENSMUSG00000032652 | cAMP responsive element binding protein like 2 | -0,74 | 0,00217 |
| 50 | Foxk1 | ENSMUSG00000056493 | forkhead box K1 | -0,711 | 0,0145 |
| 51 | Mkl2 | ENSMUSG00000009569 | MKL1/myocardin like 2 | -0,683 | 0,00157 |
| 52 | Arid5B | ENSMUSG00000019947 | AT-rich interaction domain 5B | -0,67 | 0,000163 |
| 53 | Jun | ENSMUSG00000052684 | Jun proto-oncogene, AP-1 transcription factor subunit | -0,65 | 0,00775 |
| 54 | Asxl1 | ENSMUSG00000042548 | additional sex combs like 1, transcriptional regulator | -0,648 | 0,00248 |
| 55 | Pcgf2 | ENSMUSG00000018537 | polycomb group ring finger 2 | -0,634 | 0,0465 |
| 56 | Foxp1 | ENSMUSG00000030067 | forkhead box P1 | -0,632 | 1,83E-07 |
| 57 | Kctd1 | ENSMUSG00000036225 | potassium channel tetramerization domain containing 1 | -0,631 | 0,000466 |
| 58 | Mef2C | ENSMUSG00000005583 | myocyte enhancer factor 2C | -0,617 | 0,0298 |
| 59 | Rcan1 | ENSMUSG00000022951 | regulator of calcineurin 1 | -0,606 | 0,0215 |
| 60 | Ssbp3 | ENSMUSG00000061887 | single stranded DNA binding protein 3 | -0,605 | 3,11E-06 |
| 61 | Klf13 | ENSMUSG00000052040 | Kruppel like factor 13 | -0,603 | 0,0127 |
| 62 | Ybx3 | ENSMUSG00000030189 | Y-box binding protein 3 | -0,596 | 4,47E-05 |
