# Supplementary material for: Excitatory Spinal Lhx9-Derived Interneurons Modulate Locomotor Frequency in Mice
Source: J Neurosci. 2024 Mar 4;44(18):e1607232024. doi: 10.1523/JNEUROSCI.1607-23.2024 (PMC11063822; doi:10.1523/JNEUROSCI.1607-23.2024)
Supplement: Table 1-4 — Differentially expressed transcription factors up-regulated in Vglut2-GFP+/ Shox2Cre;Rosa26-YFP- cells (Vglut2-GFP+ vs. Shox2Cre;Rosa26-YFP+ analysis) List of the differentially expressed transcription factors up-regulated in Vglut2-GFP+/ Shox2Cre;Rosa26-YFP- cells. Download Table 1-4, DOCX file. [file jneuro-44-e1607232024-s007.docx]

Table 1-4. Differentially expressed transcription factors up-regulated in Vglut2-GFP^+^/ Shox2Cre;Rosa26-YFP^-^ cells (Vglut2-GFP^+^ *vs.* Shox2Cre;Rosa26-YFP^+^ analysis)

List of the differentially expressed transcription factors up-regulated in Vglut2-GFP^+^/ Shox2Cre;Rosa26-YFP^-^ cells.

|  | Symbol | Ensembl ID | Gene Name | log2FC | padj |
| --- | --- | --- | --- | --- | --- |
| 1 | Lhx9 | ENSMUSG00000019230 | LIM homeobox 9 | 2,871 | 6,96E-16 |
| 2 | Ebf2 | ENSMUSG00000022053 | early B-cell factor 2 | 2,459 | 9,15E-18 |
| 3 | Ebf1 | ENSMUSG00000057098 | early B-cell factor 1 | 1,756 | 0,000022 |
| 4 | Barhl2 | ENSMUSG00000034384 | BarH like homeobox 2 | 1,51 | 0,0015 |
| 5 | Dmbx1 | ENSMUSG00000028707 | diencephalon/mesencephalon homeobox 1 | 1,365 | 0,00485 |
| 6 | Suv39H2 | ENSMUSG00000026646 | suppressor of variegation 3-9 homolog 2 | 1,281 | 0,00185 |
| 7 | Ebf3 | ENSMUSG00000010476 | early B-cell factor 3 | 1,268 | 6,33E-05 |
| 8 | Sp7 | ENSMUSG00000060284 | Sp7 transcription factor | 1,161 | 0,0154 |
| 9 | Rbpj | ENSMUSG00000039191 | recombination signal binding protein for immunoglobulin kappa J region | 1,135 | 1,34E-10 |
| 10 | Meox1 | ENSMUSG00000001493 | mesenchyme homeobox 1 | 1,134 | 0,0213 |
| 11 | St18 | ENSMUSG00000033740 | ST18, C2H2C-type zinc finger | 1,08 | 2,09E-05 |
| 12 | Nkx2-8 | ENSMUSG00000058669 | NK2 homeobox 8 | 1,071 | 0,0326 |
| 13 | Hmga1 | ENSMUSG00000046711 | high mobility group AT-hook 1 | 1,036 | 0,0118 |
| 14 | Foxb1 | ENSMUSG00000059246 | forkhead box B1 | 1,03 | 0,0432 |
| 15 | Kank1 | ENSMUSG00000032702 | KN motif and ankyrin repeat domains 1 | 0,958 | 2,41E-05 |
| 16 | Onecut2 | ENSMUSG00000045991 | one cut homeobox 2 | 0,943 | 0,00023 |
| 17 | Id3 | ENSMUSG00000007872 | inhibitor of DNA binding 3, HLH protein | 0,933 | 0,000107 |
| 18 | Nfkbib | ENSMUSG00000030595 | NFKB inhibitor beta | 0,924 | 3,11E-06 |
| 19 | Atrx | ENSMUSG00000031229 | ATRX, chromatin remodeler | 0,897 | 6,52E-07 |
| 20 | Neurod1 | ENSMUSG00000034701 | neuronal differentiation 1 | 0,88 | 0,00928 |
| 21 | Casp8Ap2 | ENSMUSG00000028282 | caspase 8 associated protein 2 | 0,863 | 7,35E-05 |
| 22 | Pttg1 | ENSMUSG00000020415 | pituitary tumor-transforming 1 | 0,853 | 0,00201 |
| 23 | Ankrd49 | ENSMUSG00000031931 | ankyrin repeat domain 49 | 0,845 | 0,000278 |
| 24 | Ncor2 | ENSMUSG00000029478 | nuclear receptor corepressor 2 | 0,803 | 0,0126 |
| 25 | Zfp37 | ENSMUSG00000028389 | ZFP37 zinc finger protein | 0,793 | 6,56E-06 |
| 26 | Tshz3 | ENSMUSG00000021217 | teashirt zinc finger homeobox 3 | 0,79 | 0,0207 |
| 27 | Hoxa7 | ENSMUSG00000038236 | homeobox A7 | 0,785 | 0,00637 |
| 28 | Polr3E | ENSMUSG00000030880 | RNA polymerase III subunit E | 0,784 | 6,55E-07 |
| 29 | Cops2 | ENSMUSG00000027206 | COP9 signalosome subunit 2 | 0,763 | 3,71E-06 |
| 30 | Sirt2 | ENSMUSG00000015149 | sirtuin 2 | 0,754 | 0,00775 |
| 31 | Ccar1 | ENSMUSG00000020074 | cell division cycle and apoptosis regulator 1 | 0,75 | 0,000282 |
| 32 | Foxp2 | ENSMUSG00000029563 | forkhead box P2 | 0,741 | 0,00709 |
| 33 | Znf292 | ENSMUSG00000039967 | zinc finger protein 292 | 0,725 | 1,34E-06 |
| 34 | Fhl2 | ENSMUSG00000008136 | four and a half LIM domains 2 | 0,724 | 0,000144 |
| 35 | Neurod6 | ENSMUSG00000037984 | neuronal differentiation 6 | 0,724 | 0,000702 |
| 36 | Nfkbie | ENSMUSG00000023947 | NFKB inhibitor epsilon | 0,721 | 0,0415 |
| 37 | Hoxc8 | ENSMUSG00000001657 | homeobox C8 | 0,7 | 0,0166 |
| 38 | Ncoa4 | ENSMUSG00000056234 | nuclear receptor coactivator 4 | 0,698 | 0,00101 |
| 39 | Id1 | ENSMUSG00000042745 | inhibitor of DNA binding 1, HLH protein | 0,697 | 0,00758 |
| 40 | Npat | ENSMUSG00000033054 | nuclear protein, coactivator of histone transcription | 0,679 | 0,0143 |
| 41 | Bclaf1 | ENSMUSG00000037608 | BCL2 associated transcription factor 1 | 0,651 | 2,64E-05 |
| 42 | Zeb2 | ENSMUSG00000026872 | zinc finger E-box binding homeobox 2 | 0,651 | 0,000718 |
| 43 | Tcf7L2 | ENSMUSG00000024985 | transcription factor 7 like 2 | 1 | 0,0376 |
| 44 | Gtf2H4 | ENSMUSG00000001524 | general transcription factor IIH subunit 4 | 0,645 | 0,000346 |
| 45 | Hltf | ENSMUSG00000002428 | helicase like transcription factor | 0,638 | 0,00317 |
| 46 | Nipbl | ENSMUSG00000022141 | NIPBL, cohesin loading factor | 0,615 | 0,00211 |
| 47 | Stag2 | ENSMUSG00000025862 | stromal antigen 2 | 0,609 | 0,000757 |
